# Influence of Stochastic Gene Expression on the Cell Survival Rheostat after Traumatic Brain Injury

**DOI:** 10.1371/journal.pone.0023111

**Published:** 2011-08-11

**Authors:** Daniel R. Rojo, Donald S. Prough, Michael T. Falduto, Deborah R. Boone, Maria-Adelaide Micci, Kristen M. Kahrig, Jeanna M. Crookshanks, Arnaldo Jimenez, Tatsuo Uchida, Jeremy C. Cowart, Bridget E. Hawkins, Marcela Avila, Douglas S. DeWitt, Helen L. Hellmich

**Affiliations:** 1 Department of Anesthesiology, University of Texas Medical Branch, Galveston, Texas, United States of America; 2 GenUs BioSystems, Northbrook, Illinois, United States of America; 3 Vel-Lab Research, Missouri City, Texas, United States of America; National Institutes of Health, United States of America

## Abstract

Experimental evidence suggests that random, spontaneous (stochastic) fluctuations in gene expression have important biological consequences, including determination of cell fate and phenotypic variation within isogenic populations. We propose that fluctuations in gene expression represent a valuable tool to explore therapeutic strategies for patients who have suffered traumatic brain injury (TBI), for which there is no effective drug therapy. We have studied the effects of TBI on the hippocampus because TBI survivors commonly suffer cognitive problems that are associated with hippocampal damage. In our previous studies we separated dying and surviving hippocampal neurons by laser capture microdissection and observed unexplainable variations in post-TBI gene expression, even though dying and surviving neurons were adjacent and morphologically identical. We hypothesized that, in hippocampal neurons that subsequently are subjected to TBI, randomly increased pre-TBI expression of genes that are associated with neuroprotection predisposes neurons to survival; conversely, randomly decreased expression of these genes predisposes neurons to death. Thus, to identify genes that are associated with endogenous neuroprotection, we performed a comparative, high-resolution transcriptome analysis of dying and surviving hippocampal neurons in rats subjected to TBI. We found that surviving hippocampal neurons express a distinct molecular signature — increased expression of networks of genes that are associated with regeneration, cellular reprogramming, development, and synaptic plasticity. In dying neurons we found decreased expression of genes in those networks. Based on these data, we propose a hypothetical model in which hippocampal neuronal survival is determined by a rheostat that adds injury-induced genomic signals to expression of pro-survival genes, which pre-TBI varies randomly and spontaneously from neuron to neuron. We suggest that pharmacotherapeutic strategies that co-activate multiple survival signals and enhance self-repair mechanisms have the potential to shift the cell survival rheostat to favor survival and therefore improve functional outcome after TBI.

## Introduction

After traumatic brain injury (TBI), long-term cognitive disability is associated with injury-induced neurodegeneration in the hippocampus, a region in the medial temporal lobe that is critical to learning, memory and executive function [1], [2]. One year after mild, moderate or severe TBI, 43%, 49%, and 76%, respectively, of surviving patients who had suffered TBI had residual cognitive disability [3]. Substantial increases in numbers of brain-injured soldiers returning from the Iraq and Afghanistan wars have further heightened awareness of long-term cognitive disability associated with TBI [4], [5]. Presently, there are no pharmacotherapeutic options that improve outcome in TBI patients. Unfortunately, treatments that successfully mitigated neurodegenerative signals and reduced neuronal loss in animal models of brain injury have failed to improve outcome in clinical trials [6]. Moreover, because programmed and necrotic cell death processes are essential components of normal development and basic function of all tissues, therapeutic strategies based on inhibition of these signals may have unforeseen consequences.

Based on our observation that hippocampal neurons that survive after TBI express significantly higher levels of neuroprotective genes than adjacent dying neurons [7], we speculated that a more effective therapeutic strategy would be to promote endogenous self-repair and regenerative processes in the injured brain. But how to efficiently identify the survival signals used by the brain to repair itself? The answer to this question came out of our studies using a fluid-percussion TBI model. This model is appropriate for examining endogenous protective signals in the hippocampus because in this region neuronal death after TBI is unpredictable, i.e., morphologically similar pyramidal neurons die or survive in an apparently random pattern after suffering presumably similar insults [8]. Furthermore, we found that the death or survival of hippocampal neurons correlated with differential expression of protective genes [7].

We speculated that differential neuronal vulnerability in morphologically identical neurons could result from stochasticity in gene and protein expression – unexplained fluctuations in transcription and translation of genes in individual neurons. Depending on the cellular context, stochastic fluctuations in gene expression can be beneficial or harmful and are postulated to be the underlying mechanism of phenotypic variation in genetically identical organisms [9], [10]. In cell culture, stochasticity of gene expression in clonal populations of progenitor cells can determine the choice of cell lineage [11]. *In vitro* reprogramming to induce pluripotent cells is a stochastic process that can be enhanced to increase the chances of reprogramming [12]. Moreover, stochastic mechanisms may play a fundamental role in survival strategies of various organisms [13], [14].

Therefore, we reasoned that we could identify endogenous survival signals by studying the transcriptome of neurons that are able to mount a protective response sufficient to survive an injury that leads to the death of adjacent neurons. We identified dying neurons by Fluoro-Jade staining. Although Fluoro-Jade does not distinguish between apoptotic and necrotic cell death, all types of degenerating neurons can be detected by this stain [15], [16]. Because it is impossible to study the temporal sequence of injury-induced gene expression in the same animal, we reasoned that directly comparing the transcriptome of Fluoro-Jade-negative, surviving neurons to the transcriptome of Fluoro-Jade-positive, dying neurons 24 hours post-injury would provide a snapshot of cellular signals that are activated in neurons that survive TBI.

In this study we tested the hypothesis that random fluctuation in expression of protective genes, essential for survival, would determine the fate of individual neurons when exposed to TBI. Thus, we predicted that high levels of genes involved in the brain's intrinsic survival and repair functions, would be associated with surviving neurons. We further hypothesized that differential vulnerability to TBI in a homogeneous population of hippocampal neurons reflects a cell-survival rheostat that integrates injury-induced gene expression with stochastically elevated pre-injury expression of survival-associated genes. To test this hypothesis, we directly compared the transcriptomes of dying and surviving hippocampal neurons 24 h after experimental TBI to identify differences between these two groups of neurons that could explain their differential survival.

## Results

### Impetus for transcriptional profiling study: Random sampling of stochastic gene expression

Interpretation of gene expression studies of brain injury in animal models is confounded by the brain's complexity-the mammalian brain contains 2500–5000 different cell types [17]. Neuronal heterogeneity is reflected at the genome level [18] and likely contributes to the unsuccessful translation of experimental treatments in clinical trials. For *in vivo* brain injury studies, one solution is to perform gene expression analysis of pure populations of identified neurons obtained by laser capture microdissection (LCM). Previously, in our clinically relevant rat model of moderate fluid percussion TBI [19], we used LCM to capture individual hippocampal neurons and demonstrated age-dependent and region-specific differences in the transcriptional profile of distinct subpopulations of hippocampal neurons [20]. Subsequently, we demonstrated that TBI and superimposed hemorrhagic shock suppressed expression of protective genes in dying hippocampal neurons obtained by LCM [7]. Thus, with the unprecedented single-cell resolution afforded by laser microdissection of individual neurons, we were able to study and compare the transcriptional profile of dying and surviving neurons from the hippocampal CA3 subfield, which is particularly vulnerable to fluid-percussion TBI, and gain valuable insight into the critical elements influencing neuronal survival.

**Figure 1:**
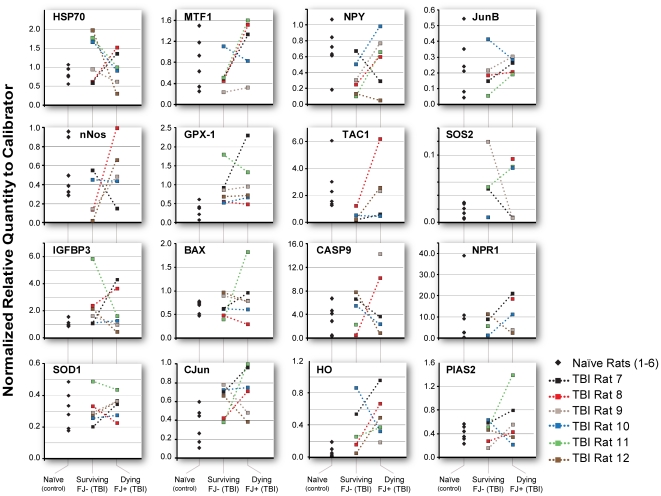
Random sampling of injury-induced gene expression. Quantitative real-time PCR analysis of injury-induced gene expression in 10 cell pools of CA3 hippocampal neurons from naïve control (to determine the normal levels in control animals of genes known to be induced by TBI) and traumatic brain injured (TBI) rats. Individual TBI rats are indicated by color. Surviving, Fluoro-Jade negative and dying Fluoro-Jade positive neurons from the same rat are connected by a dotted line. No statistically significant differences are detectable in injury-induced gene expression between dying and surviving cells. Variability in the dying cells and surviving cells was very similar for most of the genes. Gene expression was significantly larger in surviving cells than naïve cells for GPx-1 and c-Jun and significantly lower for BDNF, NPY and TAC1. Though not significant (0.05≤p<0.20) borderline differences were observed for the following genes: HSP70, HO, nNOS, SOS2, IGFBP3, MMP9: gene expression was greater in surviving cells than naïve cells for all except nNOS. Variability of gene expression in surviving cells was significantly larger than in naïve cells for HSP70, HO, SOS2, IGFBP3 and smaller for TAC1. See Table S9 for complete gene names.

We first analyzed gene expression in a small pool (10 cells) of dying and surviving hippocampal neurons 24 hours after TBI. We had previously determined empirically that quantities of total RNA isolated from smaller pools (1, 3 or 5 cells) were so variable that in many cases we could not reliably detect expression of even moderate to highly expressed genes by real-time PCR but the majority of genes that are known to be expressed in low (less than 10 copies/cell) or higher copy numbers are detectable in 10 cell samples. Based on results from our previous studies [7], [20], we expected to see significant differences between dying and surviving hippocampal neurons. Surprisingly, we found that were no statistically significant differences in injury-induced gene expression between dying and surviving cells and variability in the dying cells and surviving cells was very similar for most of the genes (Fig. 1). For some of the genes analyzed, large differences in variability could be attributed to one extreme observation. Moreover, we found that for many genes, both the variability and the magnitude of gene expression were significantly larger in surviving cells from TBI rats than in cells from naïve rats.

In all, two observations emerge from these results. First, the variability and lack of significant differences between dying and surviving neurons suggests that random noise in gene expression in a small (10 cells) pool of neurons may obscure the global effects of TBI which have been documented using larger pools of neurons. Second, the significant increases in both variability and magnitude of gene expression changes in surviving neurons versus naïve neurons suggest that surviving cells, despite being “uninjured”, nonetheless responded at a genomic level to TBI as reflected in the expression of injury-induced genes. Because in the small sampling size of 10 neurons, as reported here, the weight of the single cell on the group is higher than when assessing a larger group of cells, these observations strongly suggest that gene expression is a stochastic process at the single cell level and support our hypothesis that stochastic fluctuations in pre-injury gene expression play an important role in determining cell death or survival after TBI.

Based on these data we speculate that a cell that has high expression of genes associated with cell survival would survive after TBI, while a cell that has low expression of the same genes will die because it is more vulnerable to the effects of deleterious injury-induced genes. Moreover, we would predict that by increasing the sampling size, the probability of finding cells that have high expression of genes associated with survival is higher in surviving neurons versus dying neurons.

### Comparative transcriptome analysis of dying and adjacent surviving hippocampal neurons

To gain support for our hypothesis, we compared the whole-genome transcriptional profile of approximately 600 dying and 600 surviving hippocampal neurons after TBI. Since our objective is to understand the brain's own protective responses to injury, we decided to focus only on comparison of the two groups, dying and surviving neurons from injured rats with particular emphasis on survival and regenerative signals found in surviving neurons.

For whole-genome profiling, we laser microdissected individual pyramidal neurons from the hippocampal CA3 subfield of frozen brain sections stained with Fluoro-Jade. All Fluoro-Jade-positive neurons were presumed to be injured and dying; adjacent Fluoro-Jade-negative neurons were presumed to be uninjured and surviving. RNA samples from separate pools of Fluoro-Jade-positive and Fluoro-Jade-negative CA3 pyramidal neurons were screened for integrity and quantity. The quality of total RNA from pooled hippocampal RNA samples was assessed using the ultrasensitive Agilent Bioanalyzer Pico Chip assay (Fig. S1). The quality of double linearly amplified LCM RNA was equivalent to that of doubly amplified human RNA control samples. Furthermore, scatterplot analysis of array biological replicates (dying and surviving neurons pooled from at least three separate TBI rat brains for each replicate sample) demonstrated high concordance (Fig. S1). Following verification of RNA integrity, pooled hippocampal neuron samples were linearly amplified, labeled and hybridized to rat Agilent whole-genome arrays. Gene expression data were analyzed using Genespring GX 7.3.1 and Ingenuity Pathway Analysis software.

**Figure 2:**
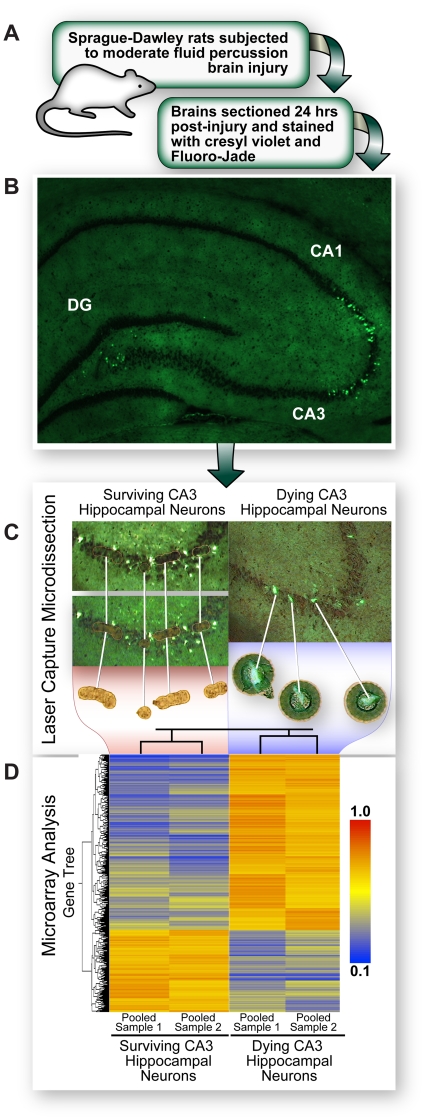
Dying and surviving neurons have distinctive gene expression signatures in rat hippocampus. Moderate TBI results in distinctive patterns of neurodegeneration in selectively vulnerable regions of the rat hippocampus. Dying, Fluoro-Jade-positive and surviving, Fluoro-Jade-negative pyramidal neurons from the CA3 subfield of the rat hippocampus were obtained by laser capture microdissection and subjected to microarray analysis. The gene tree of 2,163 genes differentially expressed greater than two-fold in dying relative to surviving neurons shows that biological replicates of pooled samples are highly concordant. The expression bar, red (1.0) to blue (0.1) indicates increased and decreased expression, respectively, compared with the median intensity of each gene in the array.

**Figure 3:**
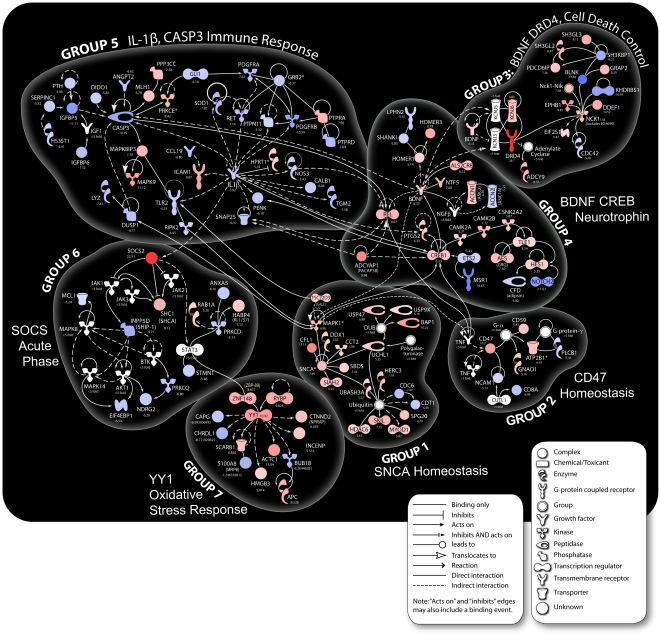
Ingenuity pathway analysis of genes that are differentially expressed >5-fold in dying vs. surviving rat hippocampal neurons. A complete network of these genes was clustered into seven custom pathways based on proximity in the network. Co-expression of functionally connected genes suggests coordinated roles in cell death or survival. Unlike standard convention, red indicates genes expressed >5-fold in surviving neurons relative to dying neurons. Blue indicates genes expressed >5-fold in dying neurons relative to surviving neurons.

Hierarchical clustering analysis confirmed that gene expression in biological replicate samples of dying neurons was concordant as was gene expression in replicates of surviving neurons; however, gene expression profiles of dying and surviving neurons were strikingly distinct (Fig. 2). The 2,163 genes in which differential expression between dying and adjacent surviving neurons exceeded two-fold involved an extraordinary range of biological processes (Gene Ontology Table S1). Gene-by-gene analysis confirmed much of what we knew about the pathophysiology of TBI but provided few insights into the critical determinants of neuronal survival. We reasoned that analysis of functionally interacting gene networks could be more informative. Therefore, we performed Ingenuity Pathway analysis with selected genes whose expression showed five-fold or greater differences between dying and surviving neurons. We generated a complete network of these genes which we then clustered into seven custom pathways based on proximity in the network and direction of the changes in expression (Fig. 3; shown enlarged in Figs. S2, S3, S4, S5, S6, S7, S8). Although the seven gene clusters are hypothetical, the groups are based strictly on known or predicted interactions of each gene with others within the network.

### Gene expression in dying or surviving neurons mirrors cell fate as defined by Fluoro-Jade staining

We observed a strong and expected correlation between the histopathology (dying or surviving neurons) and the transcriptional profiles (Table 1, complete lists in Tables S2, S3, S4, S5, S6, S7, S8), i.e., dying, Fluoro-Jade-positive neurons expressed a preponderance of genes associated with cell cycle progression (e.g., CDC6, CDT1, INPP5D), cell death (e.g., CD8A, BLNK, KHDRBS1), inflammation (e.g., CFD, CCL19, IL1β), and immune response (e.g., RIPK2, TLR2, PRKCQ). Conversely, surviving, Fluoro-Jade-negative neurons expressed high levels of a diverse array of protective, survival-associated genes involved in neuronal differentiation and growth (e.g., DDX1, DDEF1, TLE1), cell morphology (e.g., ACTC1, CTNND2), axon guidance (e.g., CFL1, SKIL, EPHB1), neurogenesis (e.g., BDNF, CREB), long-term potentiation (e.g., CAMK2A), glutamate receptor and kinase signaling (e.g., HOMER1), plasticity (e.g., MAPK1, CD47, BDNF), stress responses (e.g., MAPK9, YY1), and brain homeostasis (e.g., CCT2, ATP2B). The biological relevance of these results underscores the precise accuracy and specificity of the laser microdissection procedure in selecting dying and surviving neurons for our study.

**Table 1:** Selected Genes Found Highly Expressed in Dying or Surviving Hippocampal Neurons.

Gene	Description	Cellular Function	Ratio
BDNF	Brain-derived neurotrophic factor	Growth, development, survival, plasticity	6.35
BLNK	B-cell linker	Apoptosis, inflammatory response	**−15.82**
CAMK2A	Calcium/calmodulin-dependent protein kinase (CaM kinase) II alpha	Long-term potentiation, spatial learning, brain plasticity, development	5.31
CASP3	Caspase 3, apoptosis-related cysteine peptidase	Apoptosis, synaptic plasticity	**−10.19**
CD59	CD59 molecule, complement regulatory protein	Immune response, defense response	6.50
CDC42	Cell division cycle 42 (GTP binding protein, 25 kDa)	Apoptosis, cell cycle progression	**−5.38**
CFL1	Cofilin 1 (non-muscle)	Cytoskeletal organization and remodeling	13.15
CREB 1	cAMP responsive element binding protein 1	Survival, development, transcription regulation, synaptic plasticity	6.33
EPHB1	EPH receptor B1	Proliferation, morphogenesis, CNS development, synaptic plasticity	9.85
IL1β	Interleukin 1, beta	Inflammatory & immune response, apoptosis, embryonic dev	**−5.92**
INPP5D	(SHIP-1) Inositol polyphosphate-5-phosphatase	Apoptosis, cell cycle progression	**−9.71**
MAPK1	Mitogen-activated protein kinase 1	Cell growth, development, survival, signal transduction	6.98
MAPK9	(SAPK) Mitogen-activated protein kinase 9	Immune and stress response, growth	11.12
NCAM1	Neural cell adhesion molecule 1	Migration, growth, development	**−5.12**
NOTCH2	Notch homolog 2 (Drosophila)	Apoptosis, cell fate determination, development	**−11.07**
PDCD6IP (ALIX, AIP1)	Programmed cell death 6 interacting protein	Apoptosis, cell death regulator, cytokinesis	7.26
PTPN11	(SHP-2) Protein tyrosine phosphatase, non-receptor type 11 (Noonan syndrome 1)	Differentiation, mitogenesis, development, apoptosis	**−5.32**
REL	V-rel reticuloendotheliosis viral oncogene homolog (avian)	Development, survival, immune modulation, synaptic plasticity	8.07
RET	Ret proto-oncogene	Survival, growth, differentiation, development, functional plasticity, apoptosis	**−7.14**
RYBP	RING1 and YY1 binding protein	CNS development, transcriptional repressor, apoptosis	9.83
SKIL	SKI-like oncogene	Axonal morphogenesis, proliferation	7.75
SNCA	Synuclein, alpha (nonA4 component of amyloid precursor)	Molecular chaperone, membrane trafficking, cell viability, synaptogenesis, synaptic plasticity	7.49
TLR2	Toll-like receptor 2	Inflammatory immune response, apoptosis	**−5.75**
UCHL1	Ubiquitin carboxyl-terminal esterase L1 (ubiquitin thiolesterase)	Synaptic plasticity, cell homeostasis, development	5.35
YY1	YY1 transcription factor	Transcription regulator, stress response, proliferation, development	10.92

Positive uninjured∶injured ratios indicate increased expression in surviving neurons relative to dying neurons. Negative uninjured∶injured ratios (bold) indicate increased expression in dying neurons relative to surviving neurons.

Ingenuity pathway analysis distinguished seven prominently defined groups of interacting genes with common features that included similar biological functions (Fig. 3; Figs. S2, S3, S4, S5, S6, S7, S8; descriptions in Tables S2, S3, S4, S5, S6, S7, S8). Since functionally interacting genes are likely co-regulated and involved in similar biological processes, the co-expression of so many genes that are differentially expressed in dying and surviving neurons imply a coordinated role in cell death or cell survival. For instance, most of the genes in Groups 1, 3 and 4 that are highly expressed in surviving neurons are associated with critical cell metabolic functions and cell growth, survival and developmental pathways (References S1). Likewise, the clustering of genes associated with cell death, cell cycle regulation and stress responses in Groups 5 and 6 suggests that a cascade of functionally linked degenerative events is occurring in dying neurons.

### Unexpected gene expression associated with neuronal survival or death suggests injury-induced genes have pleiotropic functions

We were not surprised to find high expression of cell cycle regulatory genes (e.g., CDC6, CDT1, CDC42) in dying neurons. Increased expression of cell cycle genes in post-mitotic neurons is associated with apoptosis [21], [22]. However, we were surprised to find in surviving neurons high expression of several other genes that are associated with regulation of the cell cycle (e.g., HDAC6, SIAH2) and of canonical cell death-associated genes such as PDCD6IP (programmed cell death 6 interacting protein). Interestingly, for every gene associated with cell death that showed unexpectedly high expression in surviving neurons, we found published reports that suggested a pro-survival function for these genes (References S1). Indeed, several studies have shown that many pro-apoptotic genes, such as BAD and caspase-3, have dual roles in cell death or survival that depend on the cellular context [23], [24].

We also found that cell death-associated genes that were highly upregulated in dying neurons have pleiotropic pro-survival functions associated with development, neuronal remodeling and memory. For instance, the cell division cycle 42 (CDC42) gene is implicated in apoptosis but also regulates cytoskeletal dynamics and neuronal remodeling [25] and was reported to be essential for tubulogenesis and cell lineage determination in the developing mouse pancreas [26]. Dying neurons also had increased levels of the PTPN11 gene, which encodes protein tyrosine phosphatase SHP2 which is known to be involved in mitogenesis and apoptosis [27]. However, a recent study in Drosophila found that SHP2, a positive regulator of the Ras/MAPK signaling pathway, also regulates the spacing effect for long-term memory induction [28]. Genes that have previously been associated only with pro-apoptotic functions, such as Ring1 and YY1 binding protein (RYBP), showed exceptionally high expression in surviving neurons. Given the dynamic expression of this gene during embryogenesis and nervous system development [29], increased RYBP expression apparently reflects activation of the regenerative response of the injured brain. Interestingly, the alpha synuclein (SNCA) gene, which has been implicated in Parkinson's disease pathology, and found to be highly upregulated in surviving neurons, has recently been shown to act in pro-survival processes (i.e., SNCA acts as a molecular chaperone in synapses and protects against oxidative stress) [30].

### Genes involved in neural and synaptic plasticity are key to neuronal survival

**Figure 4:**
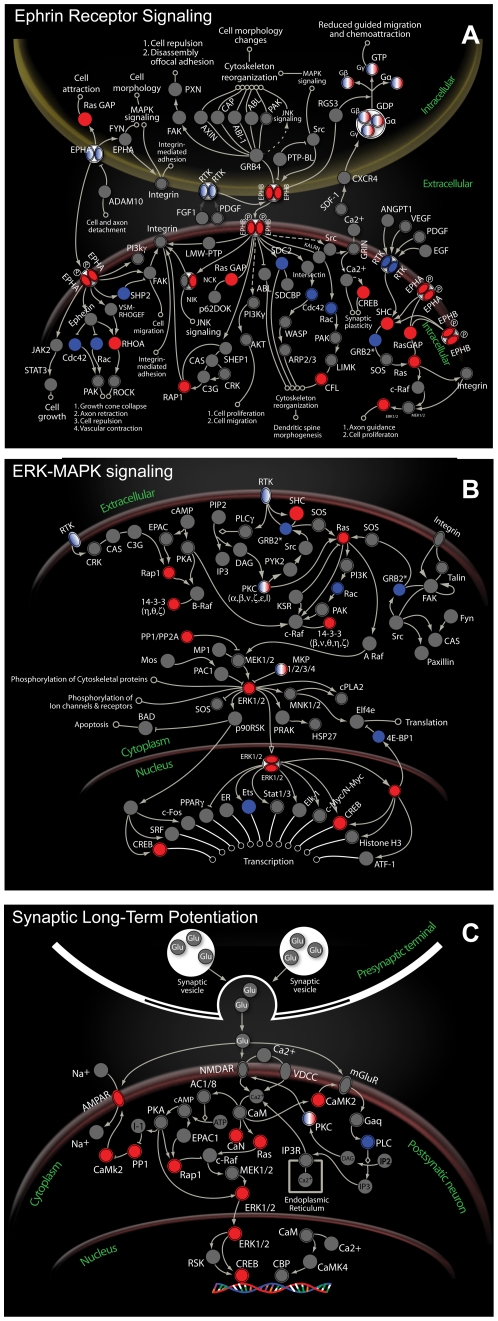
Genes in surviving neurons prominently represented in canonical pathways associated with development, survival and synaptic plasticity. ***A,*** Canonical ephrin receptor pathway. ***B,*** Canonical neurotrophin//TRK signaling pathway. ***C,*** Canonical synaptic long-term potentiation pathway. Red indicates genes expressed >5-fold in surviving neurons relative to dying neurons. Blue indicates genes expressed >5-fold in dying neurons relative to surviving neurons.

**Figure 5:**
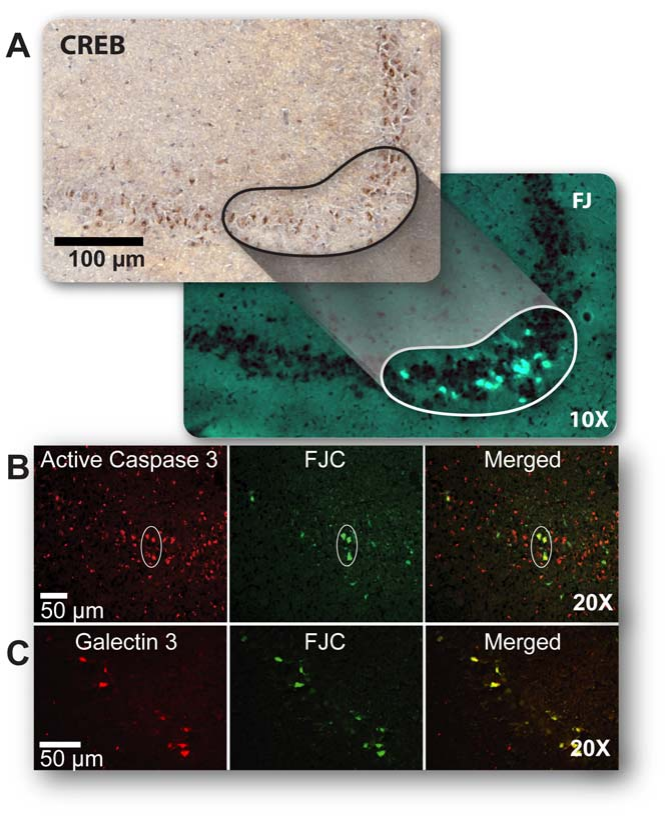
Validation of gene expression data. ***A,*** Adjacent rat hippocampal brain sections labeled with an antibody to cAMP-response element binding protein (CREB), lower panel stained with Fluoro-Jade. CREB protein expression, prominent in surviving neurons, is undetectable in dying neurons. ***B,*** Active Caspase-3 is consistently found in FJC-positive dying neurons (circled) but also is expressed in surviving neurons. ***C,*** Galectin-3, a known marker of activated microglia, is also a marker of dying neurons. It consistently co-localized with FJC positive neurons. Note, Fluoro-Jade C was used for co-localization studies. Results in (A–C) replicated in 3–4 animals for each antibody.

Consistent with previous reports, surviving hippocampal neurons expressed high levels of numerous genes associated with neural plasticity and regenerative processes such as BDNF, NTF5 and CREB. Increases in neurotrophin levels after TBI have long been associated with neuronal survival [31]. Importantly, genes that mediate synaptic plasticity such as BDNF [32] and CREB, an essential regulator of activity-dependent synaptogenesis and plasticity [33], have essential roles in neuronal development and regulate multiple cell survival genes [34]. These plasticity genes and other genes that were highly expressed in surviving neurons were also prominently represented in canonical pathways associated with development, survival and plasticity (Fig. 4A–C). The single cell resolution that facilitated our comparison of gene expression in dying and surviving neurons also allowed us to show, for the first time, that genes such as BDNF and CREB are highly expressed primarily in surviving, Fluoro-Jade-negative neurons. Immunolabeling strikingly confirmed the gene expression data, demonstrating that CREB protein expression is prominent in surviving hippocampal neurons but virtually undetectable in dying, Fluoro-Jade-positive neurons (Fig. 5A).

Clearly, one way that the brain deals with the pathological consequences of brain trauma is to reset gene expression to an earlier, embryological state at a time when developing cells were more plastic. This is a common theme that is found in other regenerating tissues; for example, mechanisms of bone repair in adults closely resemble bone formation during embryogenesis [35]. Numerous genes that have been implicated in brain development and that appear to be reactivated in the injured brain (Table 1, Tables S2, S3, S4, S5, S6, S7, S8) are also implicated in brain plasticity [36]. Moreover, multiple genes that regulate synaptic plasticity and are upregulated in surviving neurons appear to be functionally connected to these developmental genes (Fig. 3). The unexpected expression of multiple immune regulatory genes (e.g., CD59, REL, TSC22D3, MAPK9) in surviving neurons is consistent with recent studies demonstrating that many immune proteins have pleiotropic functional roles in brain development and plasticity [37].

Also consistent with our results, a recent study of recovery after stroke found that increased plasticity in surviving neurons allows previously hard-wired neurons to adopt wider functional roles to compensate for loss of stroke-damaged neurons [38]. Furthermore, a review of numerous studies of mutant mice with enhanced cognition suggests that a key common underlying mechanism is activation of signaling pathways that affect synaptic plasticity [39]. Genome-wide profiling of human brains has also implicated changes in brain plasticity as a key mechanism contributing to recovery after brain injury and to age-associated declines in cognitive function [40]. Since many lines of evidence suggest that brain plasticity mechanisms are reactivated by pharmacological drug treatment or by injury, our data indicate that increased expression of brain plasticity genes in individual neurons is a key determinant of survival after injury.

### Neuronal survival involves reactivation and reprogramming of genes involved in cell growth and development

A review of the evolution of regeneration in the CNS found that regeneration in diverse organisms recapitulates the paths taken during early embryonic CNS development, suggesting that successful regeneration and repair is facilitated by a regeneration-competent environment [41]. In surviving neurons, we identified a strong mobilization of developmental genes and a favorable environment created by concurrent increases in multiple survival-associated genes. Genes such as MAPK1 and EPHB1, which have pleiotropic roles in signal transduction, cell growth and development, were more highly expressed in surviving neurons. In addition, the majority of genes, such as CDC42 and RYBP, that have pleiotropic functions and that showed increased expression in surviving neurons, also have been shown to contribute to cellular or embryological development (Table 1; Tables S2, S3, S4, S5, S6, S7, S8). We were surprised to find that dying neurons expressed higher levels than adjacent surviving neurons of several genes known primarily for their roles in development and cell growth (e.g., NCAM1, NOTCH2 and RET). The strong mobilization of developmental genes in both surviving and dying neurons suggests that reprogramming and reactivation of transcriptional pathways involved in embryological development of the CNS may be an essential element of neuronal survival response, although in dying neurons the response is insufficient to permit recovery.

Importantly, evidence of increased expression both of genes that promote neuronal survival and those that contribute to embryological development, suggest additional therapeutic targets to improve recovery after injury. For example, Andrews et al. recently showed that functional recovery of the adult CNS was improved after nerve injury by re-expression of α9 integrin, a gene that induces neurite outgrowth during embryogenesis and is subsequently developmentally downregulated [42].

### Neuroprotection involves activation of key regulators of neuronal homeostasis

Surviving neurons expressed high levels of genes that coordinate cellular pathways critical to maintenance of cell homeostasis (i.e., stress responses), anti-inflammatory responses and mitochondrial function (e.g., SHC1, CREB, MAPK1, ATP2B1 and UCHL1). Increased expression of genes associated with regulation of cellular and neuroanatomical plasticity in surviving neurons is a clear indication that the activation of these homeostatic processes is crucial to the brain's self repair mechanisms. Examination of the gene networks associated with these regulatory genes suggest that they regulate a coordinated response to injury (i.e., counteract inflammatory signaling), maintain ionic and membrane homeostasis and key metabolic functions, all of which influence cell survival.

### Biological Relevance and Validation of Gene Expression Data

**Figure 6:**
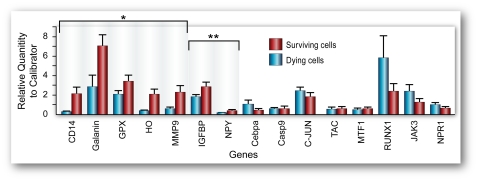
Quantitative, real-time PCR confirmation of differential gene expression in pools of dying and surviving hippocampal neurons. qPCR values represent mean and SEM (n = 7 pools for all genes, n = 6 pools for MMP-9) *p<0.05, **0.5<p<0.10, remaining genes were increased in surviving or dying neurons, supporting the trends in the Agilent arrays but were not statistically significant. Scale bars: A, 100 µm. B and C, 50 µm.

Extensive *in silico* verification that correlated gene expression data with cell fate gave us confidence in the biological relevance of our array data (Table 1; Tables S2, S3, S4, S5, S6, S7, S8, References S1). Genes highly expressed in surviving neurons were found in numerous studies to be involved in biological processes associated with survival, proliferation, regeneration and development. Conversely, genes highly expressed in dying neurons have been shown to be associated with cell death or cellular dysfunction. However, we found that genes such as Caspase 3, that were prominently expressed in dying, Fluoro-Jade-positive neurons were also expressed in many surviving neurons (Fig. 5B). Indeed our data shows that both pro-survival and pro-death genes are expressed in surviving and dying neurons; other than their differential staining with Fluoro-Jade, only the relative numbers of these genes with many diverse functions distinguish the dying from surviving neurons. This suggests that we should be cautious in interpreting the expression of biomarkers of cell death as indicative of cell fate at the level of individual neurons. Because we hypothesized that stochastic expression of neuroprotective genes would play a significant role in determining cell fate and because it is known that multiple factors such as posttranscriptional and posttranslational regulatory mechanisms and differences in half-lives of mRNA and their respective proteins may result in poor or uncertain correlation of mRNA and protein expression, [43]–[45], we did not expect that expression of all selected genes would be validated at the protein level. Nonetheless, we did observe significant correlation of mRNA and protein levels in independent brain samples; significantly, confirmation experiments (quantitative real-time PCR and immunohistochemical analysis) were performed on multiple experimental RNA samples of dying and surviving neurons (collected from minimally 6–8 brain injured rats) one to two years after the array experiments (Fig. 5A–C, Fig. 6, Table 2). Of greater import, we identified unexpected markers of cell death or cell survival in neurons; high levels of galectin-3—a known marker of activated microglia—were consistently detected primarily in dying neurons (Fig. 5C), suggesting that this gene could be also used as a marker of neuronal injury. We also found that a gene recently identified as a potential marker for brain injury, UCHL1, is highly expressed in surviving neurons and has previously been associated with synaptic plasticity and homeostasis [46].

**Table 2:** Immunohistochemical validation of gene array data.

Antibody	Support Agilent Array			
ANGIOTENSIN	•	a	Ab	+
BDNF	†	o	Ab	−
BLNK	•	o	SC	−
CASP3	†	d	IG	+
CD47	•	e	LB	+
CDC42	‡	o	GT	−
CDC6	•	c	SC	+
CREB	†	c	Ab	+
DR4	†	e	Ab	+
GALANIN	†	b	SC	+
GALECTIN-3	•	c	Ab	+
GAPDH	•	g	Ab	+
GPX1	†	o	Ab	−
HAX-1	†	o	SC	+
IL1-β	†	c	Ab	+
IFNγ	†	o	Ab	−
MMP9	•	o	NB	−
NCAM1	•	c	Ab	+
NGF	†	f	M	+
SHIP	†	c	Ab	+
SOCS2	†	d	SC	+
UCHL1	†	o	LB	−
YY1	•	o	SC	−

Results replicated in 3–4 animals for each antibody. **Antibody host:** •, mouse; †, rabbit; ‡, chicken. **Antibody dilution:** a, 1∶20; b, 1∶50; c, 1∶100; d, 1∶200; e, 1∶500; f, 1∶1000; g, 1∶5000; o, antibody did not work. **Antibody company:** Ab, Abcam; SC, Santa Cruz Biotechnology; IG, IMGenex; LB, Lifespan Biosciences; GT, Gene Tex; NB, Novus Biologicals; M, Millipore.

## Discussion

Which molecular signals, whether present before or activated by TBI, promote recovery and survival versus neuronal death? To address this question, comparison between neurons from specific brain regions in uninjured control rats and comparable regions in TBI rats is confounded by the fact that only a fraction of the neurons in any brain region die after TBI—the remainder survive. Our alternative approach is the first to investigate injury-induced gene expression in pure populations of adjacent dying and surviving hippocampal neurons, all of which have apparently suffered a similar insult. Of course, pre-insult gene expression in differentially vulnerable neurons cannot be determined. Neither can we monitor the temporal changes in injury-induced cell signals in any one group of neurons. However, analysis of networks of interacting genes that are differentially expressed in dying or surviving neurons 24 hours post-injury permitted inferences regarding neuronal protective responses that persist beyond the initial response to trauma and that involve long-term survival and regenerative signals. The results of our random sampling experiment that showed evidence of stochasticity in gene expression in neurons from uninjured rats and both dying and surviving neurons from TBI rats suggest unpredictable fluctuations in gene expression could influence the effects of TBI on vulnerable brain cells, i.e., determine whether a neuron dies or survives following TBI.

### Cell survival and cell death represent the interactions of multiple signaling networks

Because of the unexpectedly high expression in surviving neurons of cell death-associated genes and in dying neurons of protective genes, we conclude that increased expression of any one factor is unlikely to explain cell survival or death. The novel inference supported by this study is that neuronal survival is a function of the interactions of multiple signaling networks and has a distinct molecular signature (i.e., increased expression of a broad network of neuroprotective cellular signals), probably relating both to baseline, pre-injury gene expression and gene expression induced by TBI. This molecular signature is associated with cellular reprogramming processes that lead to regeneration, cell growth and plasticity.

In other complex human disorders such as common neuropsychiatric phenotypes [47], hundreds of proteins are altered, reflecting a global disruption of neuronal homeostasis. Several neurodegenerative diseases progress along functionally connected large-scale neuronal networks [48]. An analysis of common human diseases concluded that the complex phenotypes represent modulation of molecular networks by genetic and environmental factors [49]. Likewise, studying the puzzling opposing effects of NMDA receptor activation, Zhang et al. found that NMDA receptor subunits, expressed synaptically or extrasynaptically, activated distinct, multigene cell survival or cell death programs [50]. This, together with analysis of our data, is consistent with our hypothesis that survival or death of individual neurons represents the net result of an integrated network of injury-induced signals superimposed on pre-injury stochastic gene expression.

### A rheostat model of neuronal survival after TBI

**Figure 7:**
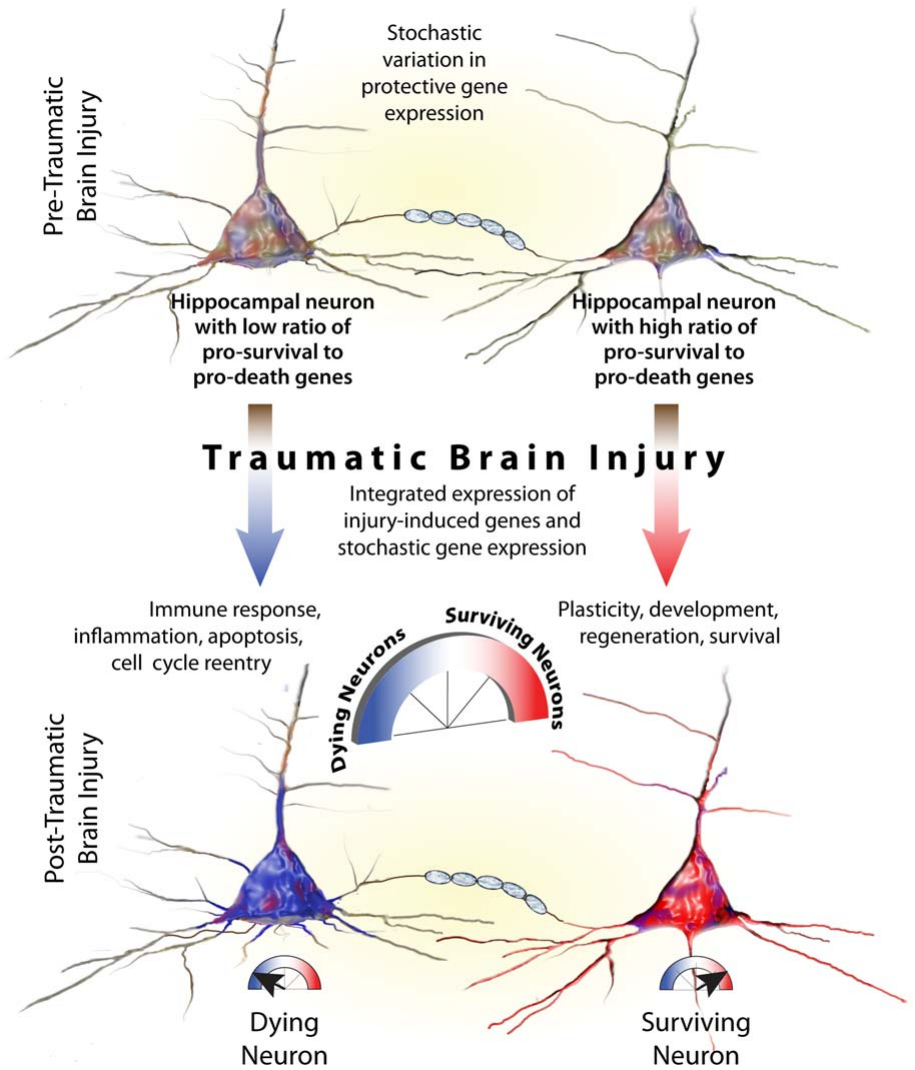
Rheostat model of neuronal survival after traumatic brain injury.

Korsmeyer et al. [51] suggested that the balance of pro- and anti-apoptotic factors, working as a cell survival rheostat, controlled the fate of cancer cells. Similarly, our data suggest that neuronal survival after TBI is regulated by a cell survival/death rheostat (Fig. 7). In all gene expression systems, specific genes show randomly fluctuating levels of expression, perhaps insuring that some proportion of cells will adapt and respond to sudden stressors [52], [53]. We suggest that, in hippocampal neurons subjected to TBI, neuronal survival is promoted by pro-survival gene networks that are upregulated either stochastically pre-injury or induced post-injury as part of the brain's protective response, and most importantly, increase the ratio of pro-survival to pro-death genes. Conversely, in dying neurons, the ratio is reversed. The cell survival rheostat has no single switch, but rather is a continuously variable function that interacts with TBI or with other injuries as they act upon hundreds of signaling pathways induced by TBI. To support of our model, we speculate that analysis of degenerating and surviving neurons in animal models of human neurological diseases such as Alzheimer's, Huntington's and Parkinson's may show similar increases in pro-death and pro-survival genes, respectively.

A cellular rheostat appears to be an essential element of ischemic preconditioning (IP), which stimulates fundamental genomic reprogramming that promotes cytoprotection and survival [54]. In a study exploring the modulatory effect of activated microglia on hippocampal neurogenesis, Battista et al. suggest that the balance between pro- and anti-inflammatory molecules provides a rheostat-like regulation [55]. The discovery of signaling networks that regulate cell death also supports the existence of a cell death/survival rheostat [56].

Molecular rheostats are ubiquitous and central to transcriptional regulation [57], metabolism [58], [59] and maintenance of homeostasis. Regulatory microRNAs fine-tune protein synthesis according to the needs of cells [43] and their dysregulation contributes to several neurodegenerative disorders [60]. MAPK-regulated gene transcription has been described as a continuously variable, rheostat-like switch [61]. Small molecule metabolites act as dynamic rheostats to fine-tune defined gene circuits by binding to chromatin-modifying enzymes and regulating the activity of transcriptional corepressors [62]. Individual genes have been shown to act as molecular rheostats. The histone deacetylase, SIRT 1, is thought to act as a rheostat to modulate the functional link between energy metabolism and circadian clock machinery [63]. The transcription factor, Sox-2, functions as a molecular rheostat for stem cell differentiation [64]. Memory formation is determined by a cellular rheostat in which the dynamic expression of CREB at the time of learning determines which neurons encode memories [65].

Interestingly, treatments that coordinately upregulate or downregulate key cell signals appear to modulate the cell-survival rheostat. For example, the anti-aging intervention, caloric restriction (CR), appears to shift metabolic rheostats that increase antioxidant defenses [66] and resemble the molecular mechanisms associated with ischemic preconditioning [67]. CR has also been shown to induce recovery of spatial memory deficits in rats subjected to global cerebral ischemia [68]. Indeed, the reported effects of both CR and IP on global neuroprotective gene expression networks directly support the concept of a cellular rheostat that integrates external and endogenous signals to increase the possibility of a beneficial functional outcome.

### Modulating the cell survival rheostat to promote neural repair after TBI

If cell survival is an emergent property of complex cell signaling networks, then polygenic therapeutic strategies may be necessary to effectively shift the neuronal death rheostat, rather than strategies targeting individual genes. In neurons that survive TBI, increased expression of genes associated with neuronal development suggests that therapeutic strategies could target networks of genes associated with development and cellular reprogramming. Indeed, our study reflects the recent observation that clinical management could be linked to molecular biology by identifying core subnetworks associated with diseases. Therapeutic strategies could be designed to influence the net output of these molecular rheostats [69]. Therapeutic application of this model to disease states other than TBI will require identification of common signaling networks associated with cell survival in surviving neurons from other neurological disorders.

In terms of prompt application of these concepts to therapy of TBI patients, several drugs that are used clinically for other purposes have the potential to alter the cell survival rheostat, perhaps by influencing the expression of gatekeeper genes that act on other genes involved in cell survival. Several therapies are currently used to treat the common sequelae of TBI, such as depression, anxiety and cognitive dysfunction. Some of the drugs used to modify these disorders activate pro-survival genes. Indeed, the beneficial effects of these drugs could be interpreted as a positive effect on the cell survival rheostat. Donepezil (Aricept) appears to improve cognition in TBI and Alzheimer's Disease patients, in part, by influencing CREB expression and CREB-mediated enhancement of neurogenesis [70]. Rolipram, an inhibitor of phosphodiesterase type IV, also activates CREB pathways and enhances neurogenesis [71] and could be therapeutic for TBI patients. In transgenic Alzheimer's mice, treatment with sildenafil, a phosphodiesterase 5 inhibitor that enhances phosphorylation of CREB, improved memory and reduced Aβ levels [72]. Several FDA-approved antidepressants exert pleiotropic effects on multiple prosurvival pathways—anti-inflammatory properties and promotion of synaptic structural plasticity [73]–[75]. Some of the effects of the antidepressant lithium on mood stabilization and neurogenesis are mediated by its inhibitory effects on a key protein, glycogen synthase kinase-3β, thus influencing a cascade of pro-survival cell signaling networks [76]. Rasagiline acts on multiple targets and may be neuroprotective in several neurodegenerative disorders [77].

Several drugs used for non-neurologic disorders also exert pleiotropic effects. Rapamycin, which inhibits microglial activation, was neuroprotective in TBI models [78], [79]. In aging mice, rapamycin's lifespan-extending effects appeared to be mediated by inhibition of the mTOR pathway, which has pleiotropic effects on autophagy, cell growth, cell-cycle progression, mitochondrial metabolism and insulin-like signaling [80]. BDNF increased expression of AMPK, which, like rapamycin, negatively regulates the mTOR pathways [81]. Perhaps drugs that activate AMPK, such as AMPK-mimetics or the antidiabetic drug metformin, could be neuroprotective after TBI [82]. These drugs all appear to work on key cell signaling cascades that result in a favorable shift in the cell survival rheostat. To support of our rheostat model of neuronal survival, in future experiments we will compare the effects of potentially neuroprotective drugs on the transcriptome of drug-treated TBI rats. We speculate that drugs which increase expression of multiple cell signaling networks associated with cell survival and regeneration will have the greatest translational potential.

Increasingly, it is accepted that understanding the function and regulation of molecular networks and how they are perturbed or affected by disease is critical to understanding the complex etiology of human disorders. Our study provides only a brief snapshot of the effects of TBI on some cell signaling networks that are involved in cell survival or cell death but our data supports our conclusion that cell fate after TBI is not only an emergent property of multiple interacting cell-survival signals but that stochastic variations in cell survival signals that exist pre-injury may influence cell fate. Although this idea is not novel, our hypothesis that cell fate is determined by the integration of injury-induced gene expression with pre-injury stochastic expression of cell death and cell survival genes in neurons subjected to brain trauma has not hitherto been considered in studies of TBI. Thus, we suggest that therapeutic interventions that mobilize multiple, endogenous regenerative cell signaling networks have the potential to significantly improve treatment and functional recovery in TBI patients. Moreover, if stochastic fluctuations in pre-injury expression of pro-survival genes can affect cell fate, there is an important implication from this hypothesis. Not unlike a preconditioning effect, some studies have shown that consumption of omega-3 fatty acids – the components in fish oil that have proven anti-inflammatory and antioxidative properties – may improve outcome after brain injury and neurodegenerative disorders suggesting that dietary interventions could influence recovery from clinical brain injury [83], [84]. Finally, instead of specific biomarkers, the global mobilization of a constellation of cell survival and regenerative signals could be utilized as a useful screening tool, a transcriptional cell survival signature, to evaluate the therapeutic potential of neuroprotective drugs for TBI and other neurodegenerative disorders.

## Materials and Methods

### Fluid percussion traumatic brain injury

The Institutional Animal Care and Use Committee of the University of Texas Medical Branch approved all animal procedures (Approval ID # 0105015 through 30 April 2012). Fluid percussion TBI was performed on 400–500 g male Sprague-Dawley rats as previously described [20].

### Sample preparation and laser capture microdissection (LCM) for real-time PCR and microarray analysis

24 h post-injury, rat brains were removed, immediately frozen in dry ice and stored at −80°C until they were prepared for cryostat sectioning and laser capture. Once the hippocampal region was reached (Bregma –3.15 mm to Bregma −4.15 mm), 10 µm coronal serial sections were collected and mounted on uncoated, precleaned superfrost glass slides (Fisher Scientific, Pittsburgh, PA). The slides were kept at −20°C until sectioning was complete. Immediately after sectioning, the frozen sections were thawed at room temperature for 30 sec and fixed for 1 min with 75% ethanol, briefly rinsed in RNase-free water (1 min), stained with 1% cresyl violet (10–15 sec), rinsed in RNase-free water (30 sec×2), stained with 0.001% Fluoro-Jade (4 min), rinsed in RNase-free water (1 min×3), then dehydrated in 95% ethanol (30 sec), 100% ethanol (30 sec) and xylene (3 min×2), air-dried for 10 to 15 min in a hood, and stored desiccated for no more than 1 hr at room temperature before LCM. All solutions were prepared with RNase-free water; and the cresyl violet and the Fluoro-Jade solutions were sterile filtered just before use. Fluoro-Jade staining clearly distinguishes dying, degenerating neurons from surviving neurons in the rat brain [8], [15], [85], and we have adapted the conventional staining protocol for maximum preservation of RNA integrity during the laser microdissection procedures.

LCM was performed using a PixCell IIe laser capture microscope with an infrared diode laser (MDS Analytical Technologies, Sunnyvale, CA). To minimize contamination from adjacent surviving Fluoro-Jade negative hippocampal CA3 pyramidal neurons, dying, Fluoro-Jade-positive CA3 neurons from the ipsilateral (directly under the injury site) rat hippocampus were captured using the smallest laser spot size (7.5 micron) with a power setting of 75–100 mW and pulse duration of 0.45–0.85 ms, but these last two settings were adjusted, as necessary, for optimum capture of the cells. Surviving, Fluoro-Jade-negative neurons were only selected when found adjacent to dying neurons. Pools of dying and surviving neurons were captured on the thermoplastic films of separate CapSure Macro LCM Caps (MDS Analytical Technologies, Sunnyvale, CA). The caps with captured cells were placed in 0.5 ml tubes with 100 µl lysis solution from the RNAquous-Micro RNA isolation kit (Ambion, Austin, TX) and vortexed for 15 sec immediately after capture, stored at −20°C and then vortexed for 30 sec before the RNA isolation procedure. Unless specified, individual neurons were pooled from 3–6 rat brains for microarray studies and pooled dying and surviving neurons from 6 or 7 separate rats were used for PCR confirmation studies.

### RNA preparation for microarray analysis

Dying and surviving neurons (approximately 600 neurons each) from at least three rats were pooled for each biological replicate sample during the RNA isolation procedure by running the RNA/lysis solution mixtures of multiple LCM caps sequentially through the same spin columns and total RNA was isolated using the RNAqueous-Micro kit according to the manufacturer's protocols. Following spin column purification, post-elution DNase1 treatment was performed according to protocols in the RNAqueous Micro kit to remove trace amounts of genomic DNA. The concentration and quality of total RNA was assessed using an Agilent Bioanalyzer with the RNA6000 Pico Lab Chip (Agilent Technologies). Only pooled RNA samples of high quality and sufficient quantity (>25 pg/µl) were used for Agilent array analysis. RNA preparation for real-time PCR analysis of array data and stochastic gene expression analysis was identical to that for microarray analysis except for the numbers of dying and surviving neurons used in these procedures (see below).

### Agilent whole-genome rat microarrays

Labeled cRNA was prepared from total RNA samples. Briefly, the Poly(A)+ RNA population within total RNA was amplified using MessageAMP II (Applied Biosystems, Foster City, CA). After a second round of reverse transcription, second-strand cDNA synthesis, and purification of double-stranded cDNA, in vitro transcription was performed using T7 RNA polymerase in the presence of Biotin-11-UTP. The quantity and quality of the cRNA was assayed by spectrophotometry and on the Agilent Bioanalyzer.

Biological replicate samples of dying and surviving hippocampal neuron total RNA were labeled and hybridized in duplicate to Agilent whole-genome rat arrays (Agilent Technologies, Santa Clara, CA). 1 µg of purified cRNA was fragmented to uniform size and applied to the microarrays in hybridization buffer. Agilent Whole-Genome rat microarrays are comprised of approximately 41,000 60-mer probes designed to conserved exons across the transcripts of targeted genes. These probes represent well annotated, full length, and partial rat gene sequences from major public databases. Arrays were hybridized at 65°C for 17 hrs in a rotating incubator and washed at 37°C for 1 min. After staining with Streptavidin-Alexa555, rinsed and dried arrays were scanned with an Agilent G2565 Microarray Scanner (Agilent Technologies, Santa Clara, CA) at 5 µm resolution. Agilent Feature Extraction software was used to process the scanned images from arrays (gridding and feature intensity extraction) and the data generated for each probe on the array was analyzed with GeneSpring GX v7.3.1 software (Agilent Technologies, Santa Clara, CA).

### Gene and Ingenuity Pathway analysis

To compare individual gene expression values across arrays, raw data from Agilent arrays was imported into GeneSpring GX 7.3.1 (Agilent Technologies, Santa Clara, CA) and then expression of individual genes normalized to the median value of each array (per chip normalization to the 50th percentile) as well as the median of each gene across all samples. Genes were removed for further analysis if at least one replicate sample was not above background intensity. Further filtering was performed to only include genes whose values were within 50% for biological replicate samples. The filtered gene list was queried for genes that have ratios greater than 2.0 and less than 0.5 (2-fold changes) in surviving or dying neurons relative to sham treatment. The filtered genes were uploaded into Ingenuity Pathway Analysis (IPA) software (Ingenuity Systems, Redwood City, CA) and mapped to the functional networks available in the Ingenuity Pathway Knowledge Base. Genes displaying 5-fold or greater changes (between dying, Fluoro-Jade-positive and surviving, Fluoro-Jade-negative neurons) in pathways and networks with highly significant enrichments based on P values were further analyzed.

Biological function analysis showed that genes associated with behavior were significantly overrepresented. Then, the behavior gene list was used as a base and expanded using the “expand” and “explore” functions from Ingenuity, to generate a complete network of genes with 5-fold difference in expression between dying and surviving neurons. The network was then clustered into seven pathways based on proximity in the network and direction of the changes in expression.

### Accession Numbers

Microarray data is MIAME compliant and raw data has been deposited in the Gene Expression Omnibus under the accession number **GSE16735**.

### Real-time quantitative PCR validation of Agilent array data

Quantitative real-time PCR was performed on a MX3000 multiplex Quantitative PCR System from Strategene (La Jolla, CA) with Taqman reagents from Applied Biosystems (Foster City, CA). Reverse transcriptase (RT) reactions were performed with reagents from the Taqman Reverse Transcriptase Reagents kit; and the AmpliTaq Gold polymerase with Gene Amp kit was used for qPCR sequences of the probe (Table S9), forward primers and reverse primers for all genes were designed using BioRad (Hercules, CA) Beacon Designer Software. The probes were labeled by Integrated DNA Technologies (Coraville, IA) with the recommended 5′dyes (FAM, Cy5, HEX or ROX) and the 3′ quenchers (Tamra NHS Ester-Sp or Iowa Black). One 50 µl RT reaction was completed for each DNase-treated RNA sample as follows. Total RNA, from approximately 300 neurons acquired by LCM, in a volume of 10 µl was added to 5 µl of 10×buffer, 11 µl of 25 mM MgCl2, 10 µl of dNTP (10 mM each dNTP), 2.5 µl of random hexamers (50 µM, 5 nmoles random hexamers), 1 µl of RNase inhibitor enzyme (20 U/ul), 1.25 µl of Multiscribe Reverse Transcriptase enzyme (50 U/µl) and 9.25 µl of nuclease-free water. The RT reactions were incubated for 10 min at 25°C, then 30 min at 48°C, and 5 min at 95°C in a Robocycler PCR machine (Stratagene). The PCR reactions were performed using 1 µl of the RT produced from the above procedure for each 25 µl PCR reaction. The PCR was performed as follows: 2.5 µl of the 10×buffer (containing 15 mM MgCl2 from the AmpliTaq Gold with Gene Amp kit), 4 µl of 25 mM MgCl2, 2 µl of 10 mM dNTPs (2.2 µl were used for 2- and 3-gene multiplexing sets and 2.5 µl were used for a four-gene set), 0.1 µl forward and reverse primers at 25–75 uM (100–300 nM in final concentration), 0.1 µl of Taqman dual labeled probe from IDT at 12.5–25 uM (50–100 nM in final concentration), and 0.125 µl of AmpliTaq gold (0.25 µl were used for a two-gene multiplexing set, 0.375 µl for a three-gene set and 0.5 µl for a fourgene set). The final volume of the reaction was brought to 25 µl with nuclease-free water. This reaction is used for a single well in the 96-well plate. The Thermal Profile setup used for the PCR reaction was one cycle for 2 min at 50°C, then one cycle for 10 min at 95°C, and a two-step PCR with 50 cycles each for 15 sec at 95°C and 1 min at 60°C.

### Validation of muliplexed real-time PCR

Each PCR reaction was performed in triplicate and standard curves were performed in triplicate for each primer and probe set and every multiplexing set using 1 µl of a 1∶10 serial dilution of a 1 µg RT reaction. Standard curve points were tested with 5 points from 20 ng to 2 pg of RT per PCR reaction. Every multiplexing set has a standard curve containing at least four of the five points (detects down to 20 pg) with an efficiency of 90%–110% and an RSq value above 0.98. Every experiment contained a no-template-negative control and a no-RT-negative control. Valid controls are those in which Ct values are always at least 5 Ct values below the lowest experimental value for that specific gene. We used GAPDH as our normalizing gene for all of our PCRs. All data from the PCR was collected and analyzed using MXPro software (Stratagene).

### Statistical analysis of gene expression in dying vs. surviving neurons

Significant differences in levels of mRNA for 27 different genes in dying and surviving neurons, as determined by quantitative real-time PCR, were assessed using the two-sample t test. Data analysis was conducted using statistical software, SAS®, Release 9.1 (SAS Institute Inc., SAS/STAT® 9.1 User's Guide, Cary, NC: SAS Institute Inc., 2004).

### Real-time PCR analysis of stochastic gene expression in naïve, surviving and dying neurons

Tissue was frozen in OCT mounting medium from six naïve and severe TBI brains, 10 µm sections were cut on a cryostat and mounted on superfrost clean slides. Sections from the TBI brain were stained with 0.001% Fluoro-Jade (Histo-chem) and counterstained with 1% cresyl violet and prepared for laser capture as described above. The naïve sections were stained only with 1% cresyl violet. LCM was performed as described above. 10 Fluoro-Jade-positive neurons (dying) and the adjacent 10 Fluoro-Jade-negative neurons (surviving) in the CA3 region of the hippocampus were collected from the same section in each TBI brain on separate CapSure Macro LCM Caps (Arcturus Engineering, Mountain View, CA). Ten neurons were also collected from the same section of each naïve brain on separate Macro caps. The caps were vortexed with a 100 ul of lysis buffer and stored at −80C until RNA isolation. RNA was isolated using the RNA Aqueous kit (Ambion) and then DNase treated at 37°C to remove any traces of genomic DNA. Total RNA was reverse transcribed using the Taqman Reverse Transcriptase Reagents kit (Applied Biosystems cat# N808-0234). Real-Time PCR was performed using a MX3000P Quantitative PCR system (Stratagene) as described previously.

### Statistical Methods for random sampling of stochastic gene expression

Due to complexity in the statistical design of this experiment, expression data were analyzed in 2 folds for each gene. Dying and surviving cells of TBI brains were from adjacent areas in the same brain. Therefore, brain (or animal) was playing a roll of “block” and death/survival was fixed effect. Those data were analyzed using analysis of variance for the randomized block design. Effects of TBI on gene expression were assessed using the mean differences of surviving cells in TBI animals and normal cells from naïve animals with the two-sample t-test. Effect of TBI on variability of gene expression among animals was assessed using the variance ratio of surviving cells to naïve cells with the F test. All tests were assessed at the 0.05 level of significance. Statistical computations were carried out using statistical software, the SAS® system, release 9.1 [86].

### Immunofluorescence and Fluoro-Jade C protocol for perfused sections

24 h after fluid percussion injury, rats were anesthetized, transcardially perfused, and fixed with freshly prepared, ice-cold, 4% paraformaldehyde in PBS (pH 7.4). Brains were removed, postfixed in 4% paraformaldehyde 1 hr at room temperature and embedded in 20% sucrose solution in PBS overnight at 4°C. Brains were frozen in O.C.T. embedding medium (Tissue Tek; Sakura, Tokyo, Japan), 16 µm thick frozen serial sections were cut on a cryostat , placed on precleaned superfrost plus slides (VWR, West Chester, PA), and stored at −80°C until needed. Frozen sections were allowed to equilibrate to room temperature and then incubated in NAOH+80% Ethanol for 5 min. Sections were then rinsed in 70% ethanol for 2 min, rinsed in de-ionized water for 2 min, incubated in 0.06% potassium permanganate for 3 min and rinsed in de-ionized water for 2 min. Sections were then blocked and permeabilized in 3% BSA with 0.3% Trition X-100 in PBS at room temperature for 30 min and then incubated in primary antibody diluted in PBS overnight at 4°C. Sections were rinsed for 5 min in PBS, and then incubated in ALEXA-conjugated secondary antibody diluted in PBS for 1 hr at room temperature (Alexa-594, 1∶250 dilution; Molecular Probes, Eugene, OR). The sections were then rinsed once in PBS for 5 min and incubated in 0.0001% Fluoro-Jade C+0.1% acetic acid for 10 min (Histochem Inc, Jefferson, Arkansas). Sections were rinsed 3 times 1 min in water, dehydrated in xylene for 1 min and coverslipped with Permount (Fisher Scientific, Houston, Texas). Staining controls were produced by omitting the primary antibodies.

### Immunofluorescence and Fluoro-Jade C protocol for fresh-frozen sections

24 h after fluid percussion TBI, rats were anesthetized and sacrificed. Brains were removed, frozen on dry ice for 10 min and then stored at −80°C until sectioned. Frozen 10 µm thick serial sections were cut on a cryostat, placed on pre-cleaned superfrost plus slides, and stored at −80°C until needed. Prior to immunolabeling, frozen sections were allowed to equilibrate to room temperature, fixed for 15 min in ice-cold, 4% paraformaldehyde and rinsed 3 times 5 min in PBS. They were then blocked in 5% normal serum in PBS for 30 min at room temperature and incubated in primary antibody diluted in PBS overnight at 4°C. The sections were rinsed 3 times 10 min in PBS, then incubated in ALEXA-conjugated secondary antibody diluted 1∶400 in PBS for 1 hr at room temperature (Alexa-594, Molecular Probes, Eugene OR). The sections were rinsed twice in PBS for 10 min, rinsed in tap water for 5 min, incubated in 0.06% potassium permanganate in tap water for 1 min and rinsed in tap water for 2 min. They were incubated in 0.0001% Fluoro-Jade C+0.1% acetic acid for 10 min (Histochem Inc., Jefferson, AR), rinsed 3 times, 1 min in tap water and finally mounted with acidic mounting media (0.1% acetic acid-80% glycerol). Staining controls were produced by omitting the primary antibodies.

### Immunohistochemistry protocol for diaminobenzidine (DAB) detection

Sections were washed in dH2O three times 5 min. Sections were then fixed in either acetone or methanol for 10 min. Sections were then washed in PBS for 5 min and incubated in 0.3% hydrogen peroxide in methanol for 20 min, rinsed in water twice for 5 min and then washed in PBS for 5 min. Sections were then incubated with a blocking solution (kit from Vector Biolabs) for 1 hr and then incubated in primary antibody that was diluted in blocking solution overnight at 4°C. Sections were rinsed in PBS three times 5 min, incubated for 30 min with diluted biotinylated secondary antibody (Vector Biolabs) at room temperature, rinsed in PBS three times 5 min and then incubated with Vectastain ABC reagent at room temperature for 45 min. Sections were rinsed in PBS three times 5 min and then incubated with DAB substrate for 2–10 min. Sections were immersed in dH20 for 5 min and then counterstained with hematoxylin and dehydrated in 95% ETOH 2×10 sec, then 100% ETOH 2×10 sec, followed by xylene 2×10 sec. Sections were air dried and then mounted with Permount.

Following immunohistochemistry, the sections were visualized using an Olympus BX51 microscope.

## Supporting Information

Figure S1 **Microarray analysis.**
(TIF)

Figure S2 **Group 1.**
(TIF)

Figure S3 **Group 2.**
(TIF)

Figure S4 **Group 3.**
(TIF)

Figure S5 **Group 4.**
(TIF)

Figure S6 **Group 5.**
(TIF)

Figure S7 **Group 6.**
(TIF)

Figure S8 **Group 7.**
(TIF)

Table S1 **Gene ontology annotations of genes differentially expressed 2-fold or greater between dying and surviving neurons.**
(DOC)

Table S2 **Group 1: SNCA Homeostasis genes differentially expressed in dying and surviving neurons.**
(DOC)

Table S3 **Group 2: CD47 homeostasis differentially expressed in dying and surviving neurons.**
(DOC)

Table S4 **Group 3: BDNF, DRD4, PDCD6IP, cell death control genes differentially expressed in dying and surviving neurons.**
(DOC)

Table S5 **Group 4: BDNF and CREB genes differentially expressed in dying and surviving neurons.**
(DOC)

Table S6 **Group 5: IL1β, CASP3 immune response genes differentially expressed in dying and surviving neurons.**
(DOC)

Table S7 **Group 6: SOCS Acute Phase genes differentially expressed in dying and surviving neurons.**
(DOC)

Table S8 **Group 7: YY1 Oxidative Stress Response genes differentially expressed in dying and surviving neurons.**
(DOC)

Table S9 **Probe and primer sequences for qPCR.**
(DOC)

References S1 **Supplementary References.**
(DOC)

## References

[1] Squire LR, Stark CE, Clark RE (2004). The medial temporal lobe.. Annu Rev Neurosci.

[2] Bast T (2007). Toward an integrative perspective on hippocampal function: from the rapid encoding of experience to adaptive behavior.. Rev Neurosci.

[3] Thornhill S, Teasdale GM, Murray GD, McEwen J, Roy CW (2000). Disability in young people and adults one year after head injury: prospective cohort study.. BMJ.

[4] French LM, Parkinson GW (2008). Assessing and treating veterans with traumatic brain injury.. J Clin Psychol.

[5] Warden D (2006). Military TBI During the Iraq and Afghanistan Wars.. J Head Trauma Rehabil.

[6] Schouten JW (2007). Neuroprotection in traumatic brain injury: a complex struggle against the biology of nature.. Curr Opin Crit Care.

[7] Hellmich HL, Garcia JM, Shimamura M, Shah SA, Avila MA (2005). Traumatic brain injury and hemorrhagic hypotension suppress neuroprotective gene expression in injured hippocampal neurons.. Anesthesiology.

[8] Hellmich HL, Eidson KA, Capra BA, Garcia JM, Boone DR (2006). Injured Fluoro-Jade-positive hippocampal neurons contain high levels of zinc after traumatic brain injury.. Brain Res.

[9] Raj A, Rifkin SA, Andersen E, van Oudenaarden A (2010). Variability in gene expression underlies incomplete penetrance.. Nature.

[10] Raj A, van Oudenaarden A (2008). Nature, nurture, or chance: stochastic gene expression and its consequences.. Cell.

[11] Chang HH, Hemberg M, Barahona M, Ingber DE, Huang S (2008). Transcriptome-wide noise controls lineage choice in mammalian progenitor cells.. Nature.

[12] Hanna J, Saha K, Pando B, van ZJ, Lengner CJ (2009). Direct cell reprogramming is a stochastic process amenable to acceleration.. Nature.

[13] Fraser D, Kaern M (2009). A chance at survival: gene expression noise and phenotypic diversification strategies.. Mol Microbiol.

[14] Losick R, Desplan C (2008). Stochasticity and cell fate.. Science.

[15] Schmued LC, Albertson C, Slikker W (1997). Fluoro-Jade: a novel fluorochrome for the sensitive and reliable histochemical localization of neuronal degeneration.. Brain Res.

[16] Ye X, Carp RI, Schmued LC, Scallet AC (2001). Fluoro-Jade and silver methods: application to the neuropathology of scrapie, a transmissible spongiform encephalopathy.. Brain Res Brain Res Protoc.

[17] Bota M, Dong HW, Swanson LW (2003). From gene networks to brain networks.. Nat Neurosci.

[18] Lein ES, Hawrylycz MJ, Ao N, Ayres M, Bensinger A (2007). Genome-wide atlas of gene expression in the adult mouse brain.. Nature.

[19] Hoge CW, McGurk D, Thomas JL, Cox AL, Engel CC (2008). Mild traumatic brain injury in U.S. Soldiers returning from Iraq.. N Engl J Med.

[20] Shimamura M, Garcia JM, Prough DS, Hellmich HL (2004). Laser capture microdissection and analysis of amplified antisense RNA from distinct cell populations of the young and aged rat brain: effect of traumatic brain injury on hippocampal gene expression.. Mol Brain Res.

[21] Herrup K, Busser JC (1995). The induction of multiple cell cycle events precedes target-related neuronal death.. Development.

[22] Nagy Z (2000). Cell cycle regulatory failure in neurones: causes and consequences.. Neurobiol Aging.

[23] McLaughlin B, Hartnett KA, Erhardt JA, Legos JJ, White RF (2003). Caspase 3 activation is essential for neuroprotection in preconditioning.. Proc Natl Acad Sci U S A.

[24] Seo SY, Chen YB, Ivanovska I, Ranger AM, Hong SJ (2004). BAD is a pro-survival factor prior to activation of its pro-apoptotic function.. J Biol Chem.

[25] Alberts P, Rudge R, Irinopoulou T, Danglot L, Gauthier-Rouviere C (2006). Cdc42 and actin control polarized expression of TI-VAMP vesicles to neuronal growth cones and their fusion with the plasma membrane.. Mol Biol Cell.

[26] Kesavan G, Sand FW, Greiner TU, Johansson JK, Kobberup S (2009). Cdc42-mediated tubulogenesis controls cell specification.. Cell.

[27] Grossmann KS, Wende H, Paul FE, Cheret C, Garratt AN (2009). The tyrosine phosphatase Shp2 (PTPN11) directs Neuregulin-1/ErbB signaling throughout Schwann cell development.. Proc Natl Acad Sci U S A.

[28] Pagani MR, Oishi K, Gelb BD, Zhong Y (2009). The phosphatase SHP2 regulates the spacing effect for long-term memory induction.. Cell.

[29] Pirity MK, Locker J, Schreiber-Agus N (2005). Rybp/DEDAF is required for early postimplantation and for central nervous system development.. Mol Cell Biol.

[30] Chandra S, Gallardo G, Fernandez-Chacon R, Schluter OM, Sudhof TC (2005). Alpha-synuclein cooperates with CSPalpha in preventing neurodegeneration.. Cell.

[31] Almeida RD, Manadas BJ, Melo CV, Gomes JR, Mendes CS (2005). Neuroprotection by BDNF against glutamate-induced apoptotic cell death is mediated by ERK and PI3-kinase pathways.. Cell Death Differ.

[32] Lynch G, Kramar EA, Rex CS, Jia Y, Chappas D (2007). Brain-derived neurotrophic factor restores synaptic plasticity in a knock-in mouse model of Huntington's disease.. J Neurosci.

[33] Josselyn SA, Nguyen PV (2005). CREB, synapses and memory disorders: past progress and future challenges.. Curr Drug Targets CNS Neurol Disord.

[34] Mayr B, Montminy M (2001). Transcriptional regulation by the phosphorylation-dependent factor CREB.. Nat Rev Mol Cell Biol.

[35] Deschaseaux F, Sensebe L, Heymann D (2009). Mechanisms of bone repair and regeneration.. Trends Mol Med.

[36] Johnston MV (2009). Plasticity in the developing brain: implications for rehabilitation.. Dev Disabil Res Rev.

[37] Boulanger LM (2009). Immune proteins in brain development and synaptic plasticity.. Neuron.

[38] Winship IR, Murphy TH (2008). In vivo calcium imaging reveals functional rewiring of single somatosensory neurons after stroke.. J Neurosci.

[39] Lee YS, Silva AJ (2009). The molecular and cellular biology of enhanced cognition.. Nat Rev Neurosci.

[40] Lu T, Pan Y, Kao SY, Li C, Kohane I (2004). Gene regulation and DNA damage in the ageing human brain.. Nature.

[41] Tanaka EM, Ferretti P (2009). Considering the evolution of regeneration in the central nervous system.. Nat Rev Neurosci.

[42] Andrews MR, Czvitkovich S, Dassie E, Vogelaar CF, Faissner A (2009). Alpha9 integrin promotes neurite outgrowth on tenascin-C and enhances sensory axon regeneration.. J Neurosci.

[43] Baek D, Villen J, Shin C, Camargo FD, Gygi SP (2008). The impact of microRNAs on protein output.. Nature.

[44] Fortini ME (2009). Notch signaling: the core pathway and its posttranslational regulation.. Dev Cell.

[45] Gygi SP, Rochon Y, Franza BR, Aebersold R (1999). Correlation between protein and mRNA abundance in yeast.. Mol Cell Biol.

[46] Papa L, Akinyi L, Liu MC, Pineda J, Tepas J (2009). Ubiquitin C-terminal hydrolase is a novel biomarker in humans for severe traumatic brain injury*.. Crit Care Med.

[47] Ramocki MB, Zoghbi HY (2008). Failure of neuronal homeostasis results in common neuropsychiatric phenotypes.. Nature.

[48] Seeley WW, Crawford RK, Zhou J, Miller BL, Greicius MD (2009). Neurodegenerative diseases target large-scale human brain networks.. Neuron.

[49] Chen Y, Zhu J, Lum PY, Yang X, Pinto S (2008). Variations in DNA elucidate molecular networks that cause disease.. Nature.

[50] Zhang SJ, Steijaert MN, Lau D, Schutz G, Delucinge-Vivier C (2007). Decoding NMDA receptor signaling: identification of genomic programs specifying neuronal survival and death.. Neuron.

[51] Korsmeyer SJ, Shutter JR, Veis DJ, Merry DE, Oltvai ZN (1993). Bcl-2/Bax: a rheostat that regulates an anti-oxidant pathway and cell death.. Semin Cancer Biol.

[52] Thattai M, van OA (2004). Stochastic gene expression in fluctuating environments.. Genetics.

[53] Kaern M, Elston TC, Blake WJ, Collins JJ (2005). Stochasticity in gene expression: from theories to phenotypes.. Nat Rev Genet.

[54] Dirnagl U, Becker K, Meisel A (2009). Preconditioning and tolerance against cerebral ischaemia: from experimental strategies to clinical use.. Lancet Neurol.

[55] Battista D, Ferrari CC, Gage FH, Pitossi FJ (2006). Neurogenic niche modulation by activated microglia: transforming growth factor beta increases neurogenesis in the adult dentate gyrus.. Eur J Neurosci.

[56] Hitomi J, Christofferson DE, Ng A, Yao J, Degterev A (2008). Identification of a molecular signaling network that regulates a cellular necrotic cell death pathway.. Cell.

[57] Han JS, Szak ST, Boeke JD (2004). Transcriptional disruption by the L1 retrotransposon and implications for mammalian transcriptomes.. Nature.

[58] Armstrong JS, Whiteman M, Yang H, Jones DP (2004). The redox regulation of intermediary metabolism by a superoxide-aconitase rheostat.. Bioessays.

[59] Bigelow DJ, Squier TC (2005). Redox modulation of cellular signaling and metabolism through reversible oxidation of methionine sensors in calcium regulatory proteins.. Biochim Biophys Acta.

[60] Hebert SS, De SB (2009). Alterations of the microRNA network cause neurodegenerative disease.. Trends Neurosci.

[61] Hazzalin CA, Mahadevan LC (2002). MAPK-regulated transcription: a continuously variable gene switch?. Nat Rev Mol Cell Biol.

[62] Ladurner AG (2006). Rheostat control of gene expression by metabolites.. Mol Cell.

[63] Grimaldi B, Nakahata Y, Kaluzova M, Masubuchi S, Sassone-Corsi P (2009). Chromatin remodeling, metabolism and circadian clocks: the interplay of CLOCK and SIRT1.. Int J Biochem Cell Biol.

[64] Kopp JL, Ormsbee BD, Desler M, Rizzino A (2008). Small increases in the level of Sox2 trigger the differentiation of mouse embryonic stem cells.. Stem cells.

[65] Han JH, Kushner SA, Yiu AP, Cole CJ, Matynia A (2007). Neuronal competition and selection during memory formation.. Science.

[66] Masoro EJ (2000). Caloric restriction and aging: an update.. Exp Gerontol.

[67] Fontan-Lozano A, Lopez-Lluch G, Delgado-Garcia JM, Navas P, Carrion AM (2008). Molecular Bases of Caloric Restriction Regulation of Neuronal Synaptic Plasticity.. Mol Neurobiol.

[68] Roberge MC, Messier C, Staines WA, Plamondon H (2008). Food restriction induces long-lasting recovery of spatial memory deficits following global ischemia in delayed matching and non-matching-to-sample radial arm maze tasks.. Neuroscience.

[69] Schadt EE (2009). Molecular networks as sensors and drivers of common human diseases.. Nature.

[70] Kotani S, Yamauchi T, Teramoto T, Ogura H (2006). Pharmacological evidence of cholinergic involvement in adult hippocampal neurogenesis in rats.. Neuroscience.

[71] Nakagawa S, Kim JE, Lee R, Malberg JE, Chen J (2002). Regulation of neurogenesis in adult mouse hippocampus by cAMP and the cAMP response element-binding protein.. J Neurosci.

[72] Puzzo D, Staniszewski A, Deng SX, Privitera L, Leznik E (2009). Phosphodiesterase 5 inhibition improves synaptic function, memory, and amyloid-beta load in an Alzheimer's disease mouse model.. J Neurosci.

[73] Benekareddy M, Mehrotra P, Kulkarni VA, Ramakrishnan P, Dias BG (2008). Antidepressant treatments regulate matrix metalloproteinases-2 and -9 (MMP-2/MMP-9) and tissue inhibitors of the metalloproteinases (TIMPS 1–4) in the adult rat hippocampus.. Synapse.

[74] Lim CM, Kim SW, Park JY, Kim C, Yoon SH (2008). Fluoxetine affords robust neuroprotection in the postischemic brain via its anti-inflammatory effect.. J Neurosci Res.

[75] Paizanis E, Kelai S, Renoir T, Hamon M, Lanfumey L (2007). Life-long hippocampal neurogenesis: environmental, pharmacological and neurochemical modulations.. Neurochem Res.

[76] Wada A (2009). Lithium and neuropsychiatric therapeutics: neuroplasticity via glycogen synthase kinase-3beta, beta-catenin, and neurotrophin cascades.. J Pharmacol Sci.

[77] Weinreb O, Mandel S, Bar-Am O, Yogev-Falach M, vramovich-Tirosh Y (2009). Multifunctional neuroprotective derivatives of rasagiline as anti-Alzheimer's disease drugs.. Neurotherapeutics.

[78] Erlich S, Alexandrovich A, Shohami E, Pinkas-Kramarski R (2007). Rapamycin is a neuroprotective treatment for traumatic brain injury.. Neurobiol Dis.

[79] Hailer NP (2008). Immunosuppression after traumatic or ischemic CNS damage: it is neuroprotective and illuminates the role of microglial cells.. Prog Neurobiol.

[80] Harrison DE, Strong R, Sharp ZD, Nelson JF, Astle CM (2009). Rapamycin fed late in life extends lifespan in genetically heterogeneous mice.. Nature.

[81] Gomez-Pinilla F, Vaynman S, Ying Z (2008). Brain-derived neurotrophic factor functions as a metabotrophin to mediate the effects of exercise on cognition.. Eur J Neurosci.

[82] Steinberg GR, Kemp BE (2009). AMPK in Health and Disease.. Physiol Rev.

[83] Mills JD, Bailes JE, Sedney CL, Hutchins H, Sears B (2011). Omega-3 fatty acid supplementation and reduction of traumatic axonal injury in a rodent head injury model.. J Neurosurg.

[84] Palacios-Pelaez R, Lukiw WJ, Bazan NG (2010). Omega-3 essential fatty acids modulate initiation and progression of neurodegenerative disease.. Mol Neurobiol.

[85] Hellmich HL, Capra B, Eidson K, Garcia J, Kennedy D (2005). Dose-dependent neuronal injury after traumatic brain injury.. Brain Res.

[86] SAS Institute, Inc. (2004). SAS/STAT User's Guide. Version 9.1.

