# Supplementary figures and images for: Influence of Stochastic Gene Expression on the Cell Survival Rheostat after Traumatic Brain Injury
Source: PLoS One. 2011 Aug 11;6(8):e23111. doi: 10.1371/journal.pone.0023111 (PMC3154935; doi:10.1371/journal.pone.0023111)

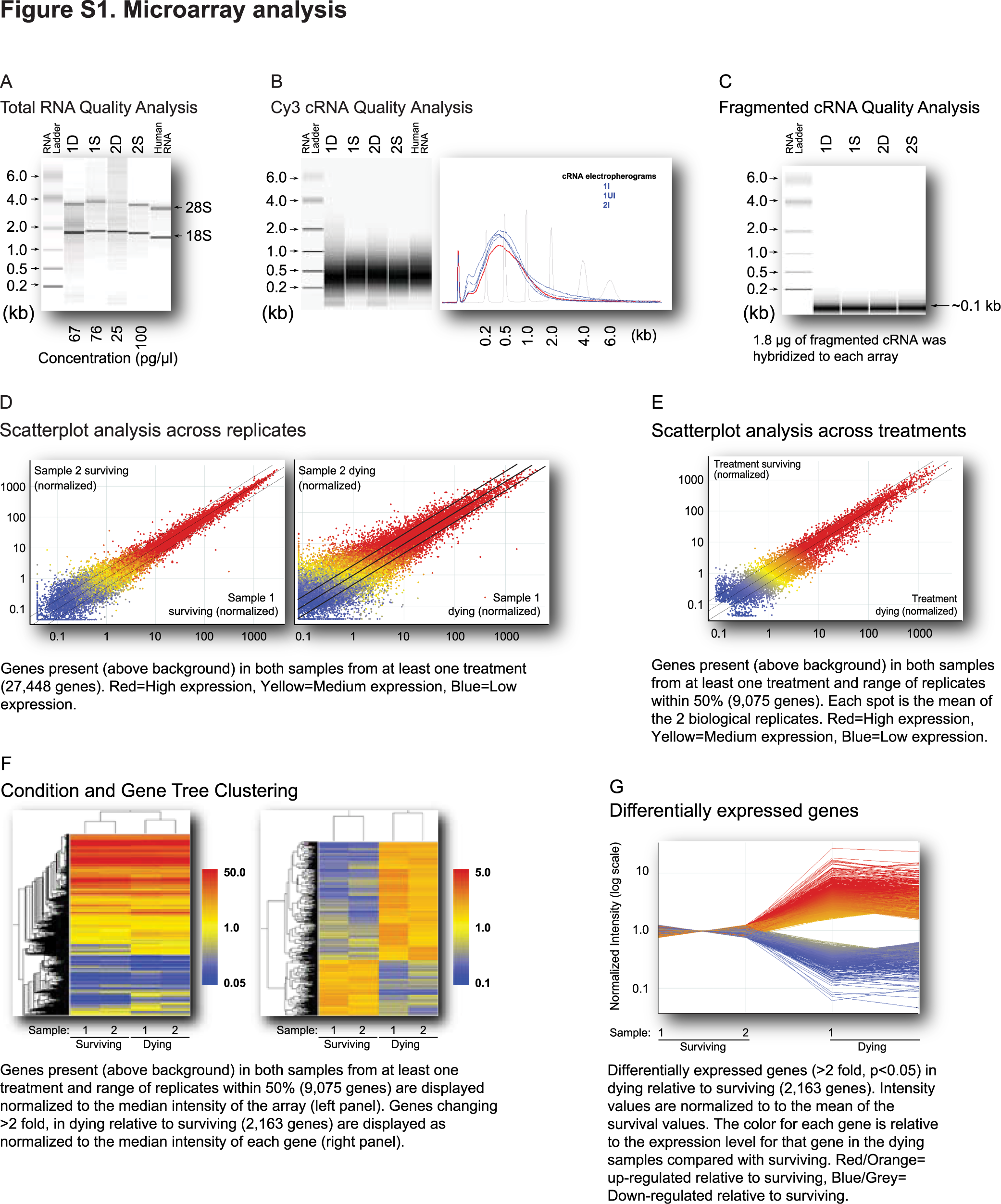

Supplement: Figure S1 — Microarray analysis. (TIF) [file pone.0023111.s001.tif]

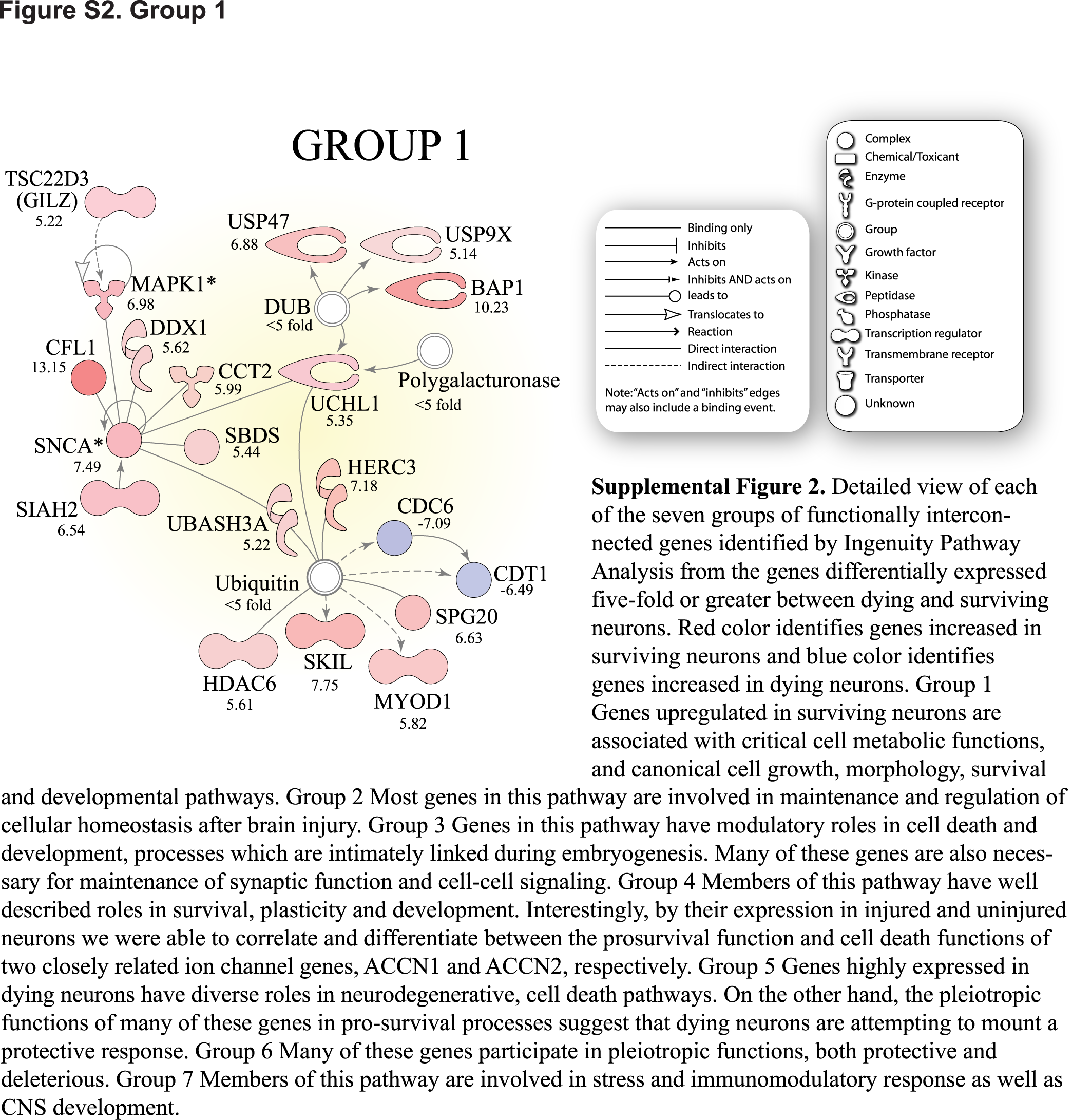

Supplement: Figure S2 — Group 1. (TIF) [file pone.0023111.s002.tif]

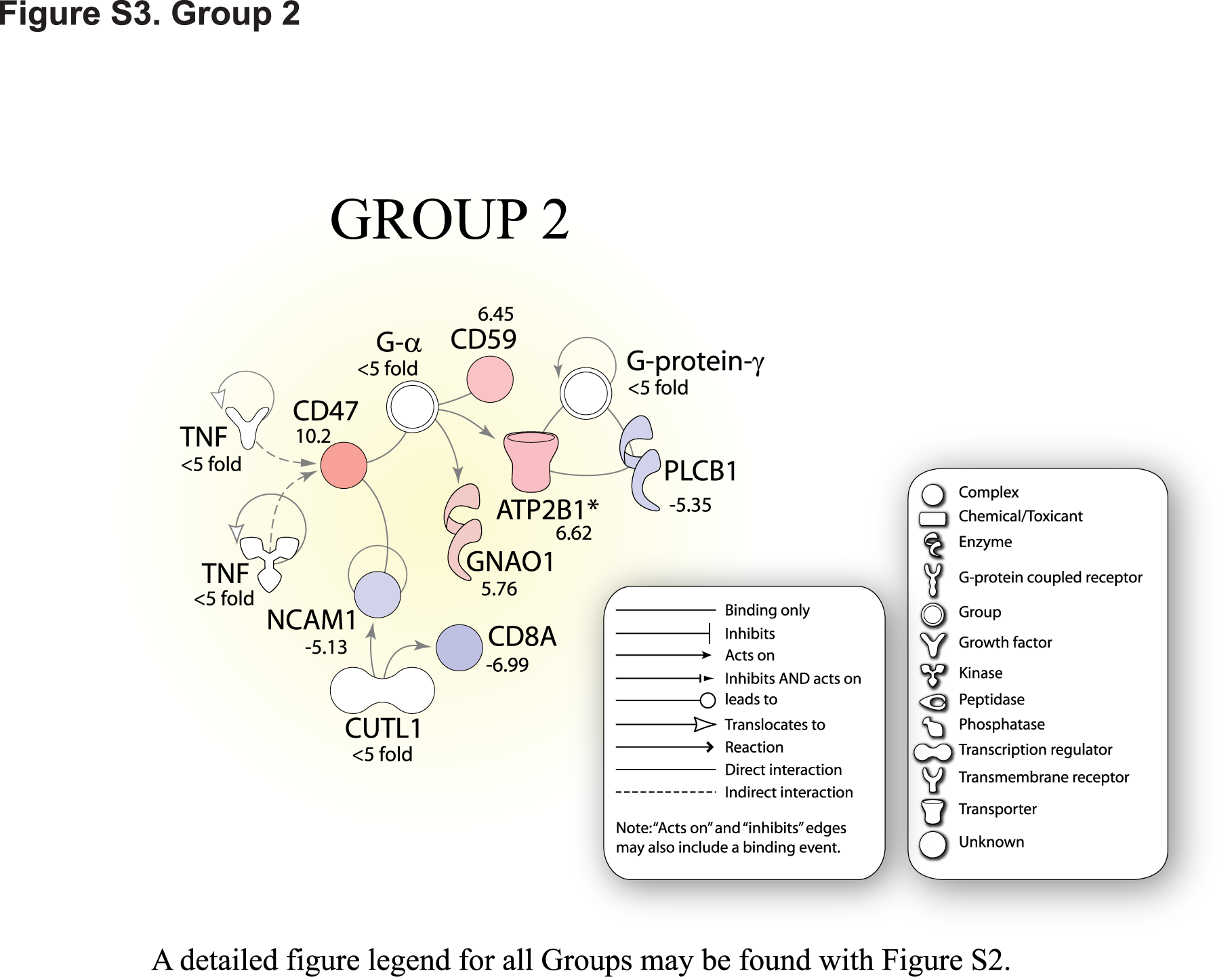

Supplement: Figure S3 — Group 2. (TIF) [file pone.0023111.s003.tif]

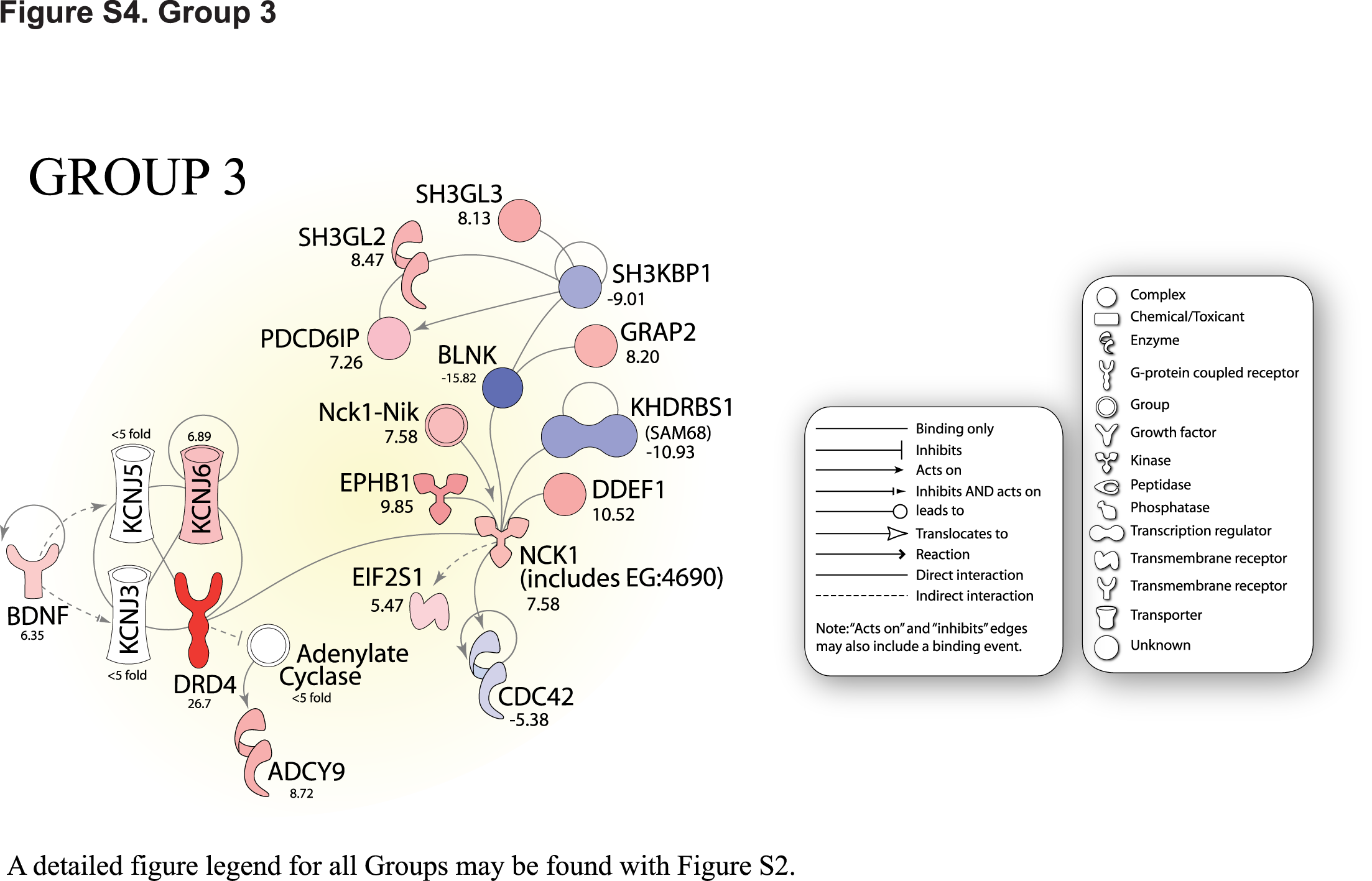

Supplement: Figure S4 — Group 3. (TIF) [file pone.0023111.s004.tif]

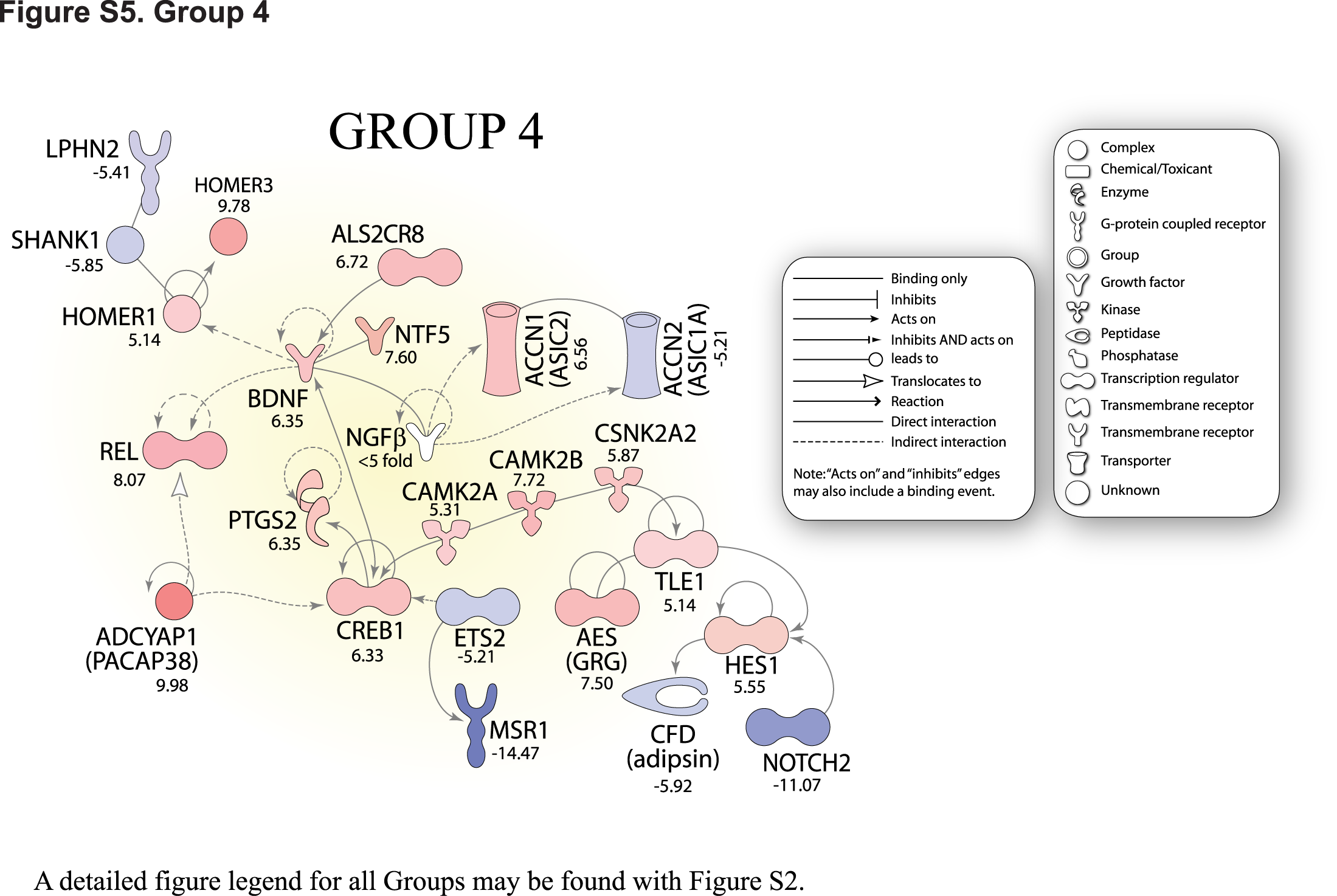

Supplement: Figure S5 — Group 4. (TIF) [file pone.0023111.s005.tif]

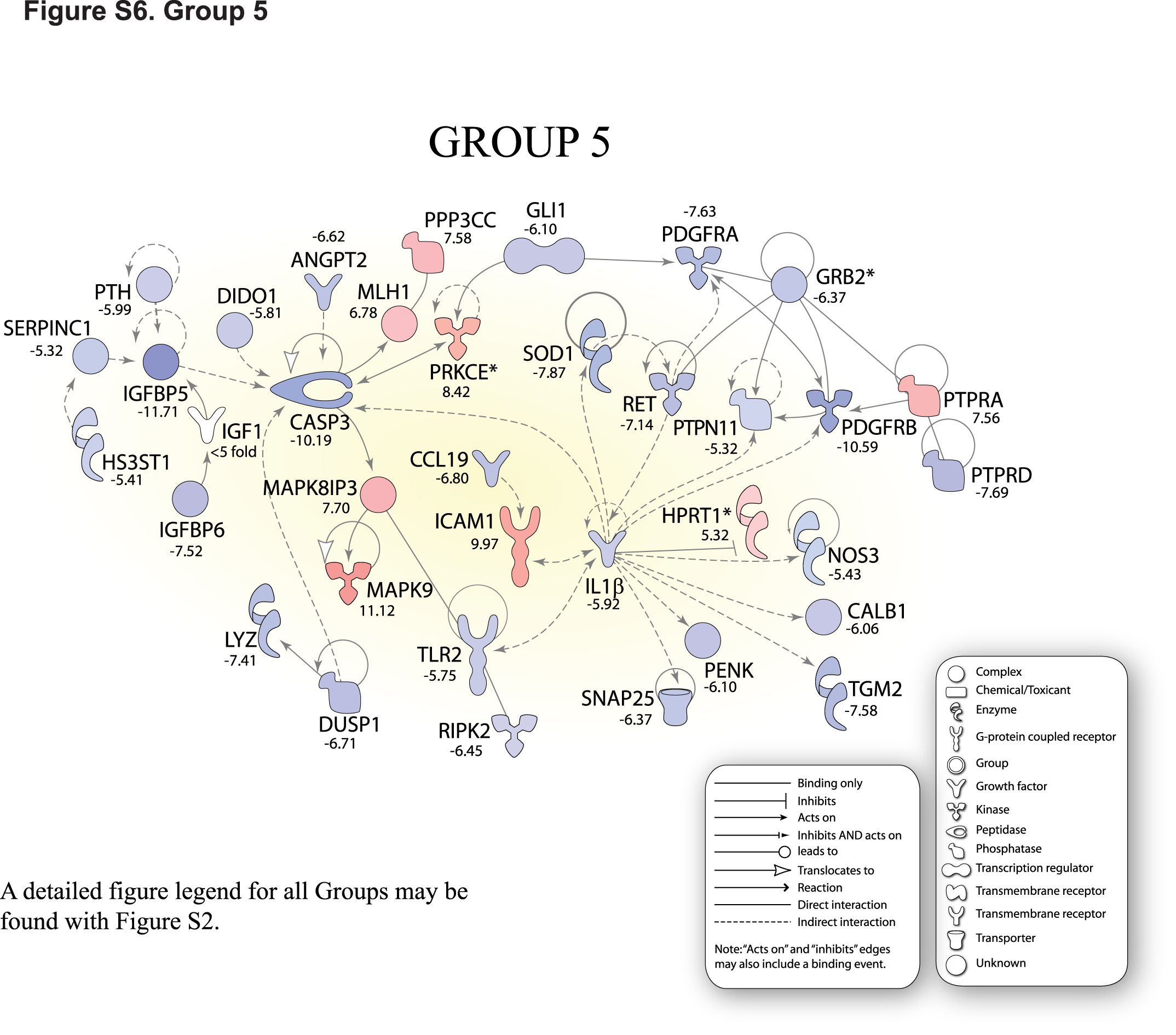

Supplement: Figure S6 — Group 5. (TIF) [file pone.0023111.s006.tif]

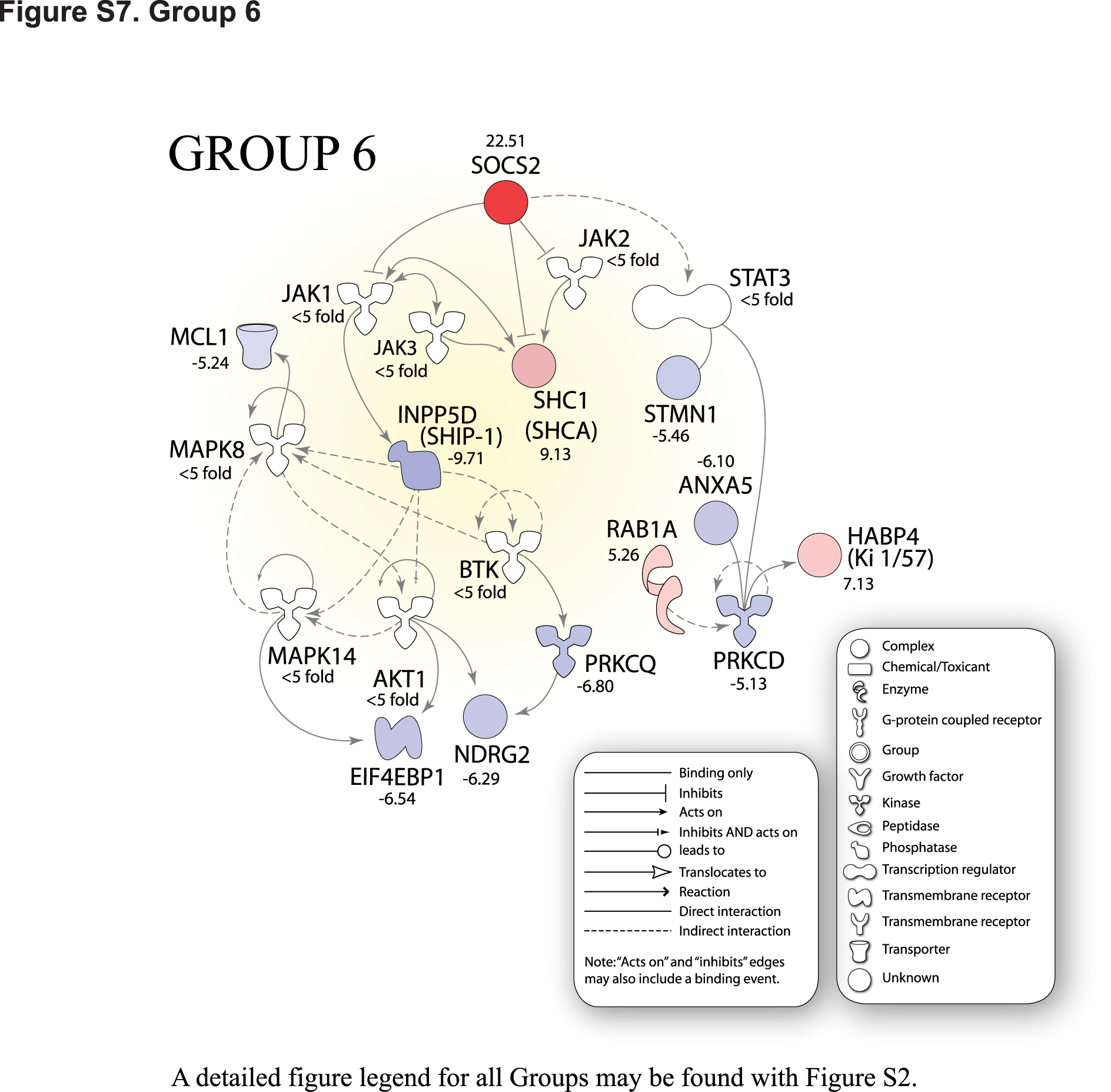

Supplement: Figure S7 — Group 6. (TIF) [file pone.0023111.s007.tif]

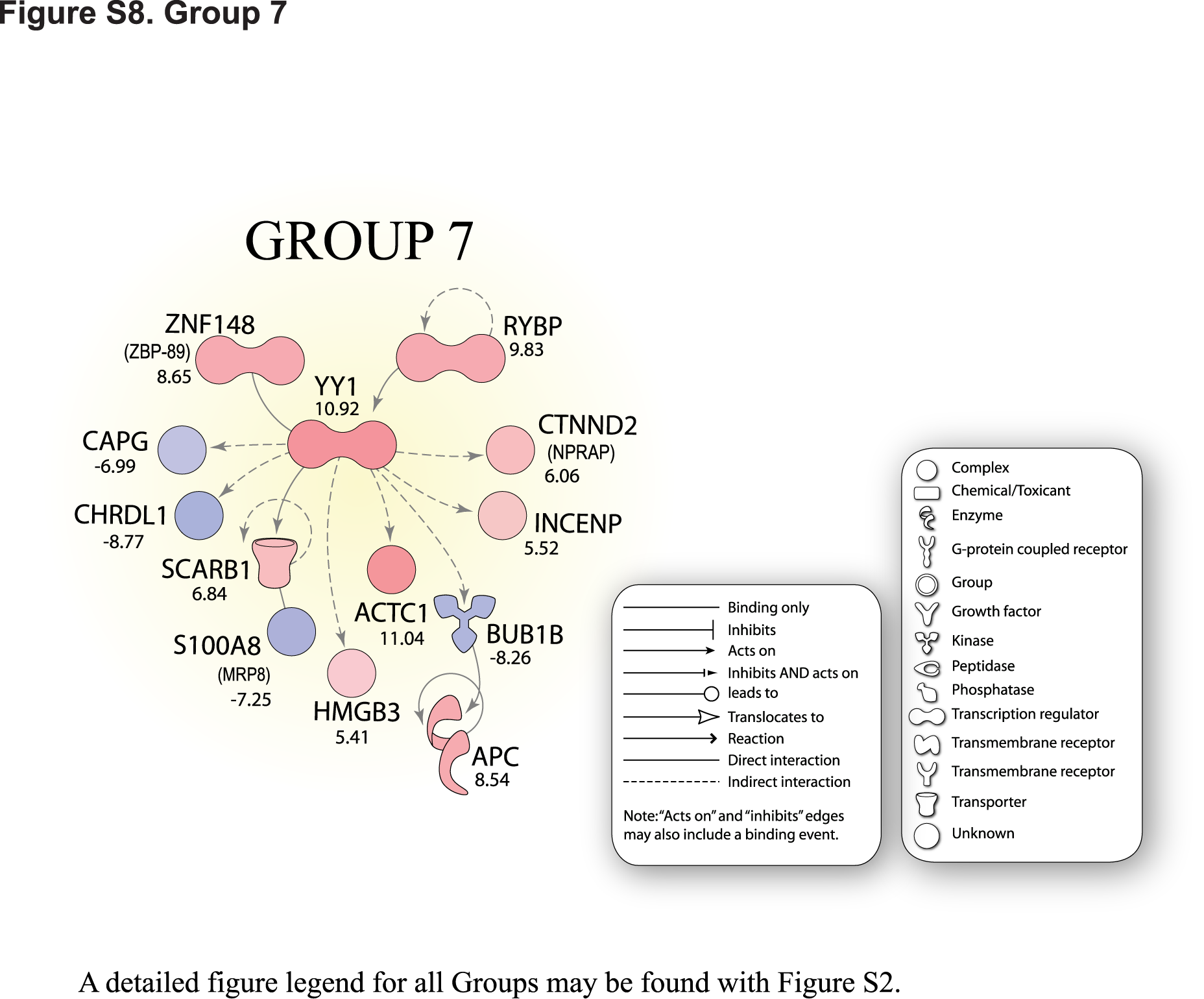

Supplement: Figure S8 — Group 7. (TIF) [file pone.0023111.s008.tif]
