# Supplementary material for: Influence of Stochastic Gene Expression on the Cell Survival Rheostat after Traumatic Brain Injury
Source: PLoS One. 2011 Aug 11;6(8):e23111. doi: 10.1371/journal.pone.0023111 (PMC3154935; doi:10.1371/journal.pone.0023111)
Supplement: Table S1 — Gene ontology annotations of genes differentially expressed 2-fold or greater between dying and surviving neurons. (DOC) [file pone.0023111.s009.doc]

**Table-S 1.** Gene ontology annotations of genes differentially expressed 2-fold or greater between dying and surviving neurons.

| **Category** |  | **Genes in Category** | **Genes in List**  **in Category** | **p-Value** |
| --- | --- | --- | --- | --- |
| **GO Nbr** | **Biological processes** |  |  |  |
| 6725 | Aromatic compound metabolism | 169 | 18 | 0.000107 |
| 6575 | Amino acid derivative metabolism | 144 | 15 | 0.000498 |
| 51234 | Establishment of localization | 3892 | 187 | 0.000511 |
| 51179 | Localization | 3938 | 188 | 0.000686 |
| 6220 | Pyrimidine nucleotide metabolism | 29 | 6 | 0.000752 |
| 6955 | Immune response | 897 | 54 | 0.000944 |
| 6600 | Creatine metabolism | 6 | 3 | 0.001080 |
| 9613 | Response to pest, pathogen or parasite | 502 | 34 | 0.001260 |
| 6958 | Complement activation, classical pathway | 32 | 6 | 0.001300 |
| 46498 | S-adenosylhomocysteine metabolism | 22 | 5 | 0.001340 |
| 45087 | Innate immune response | 45 | 7 | 0.001640 |
| 16064 | Humoral defense mechanism (sensu Vertebrata) | 58 | 8 | 0.001740 |
| 6810 | Transport | 3341 | 160 | 0.001810 |
| 45620 | Negative regulation of lymphocyte differentiation | 7 | 3 | 0.001830 |
| 6956 | Complement activation | 46 | 7 | 0.001870 |
| 46500 | S-adenosylmethionine metabolism | 24 | 5 | 0.002020 |
| 9607 | Response to biotic stimulus | 994 | 57 | 0.002100 |
| 6119 | Oxidative phosphorylation | 88 | 10 | 0.002190 |
| 6950 | Response to stress | 1398 | 75 | 0.002630 |
| 6164 | Purine nucleotide biosynthesis | 106 | 11 | 0.002780 |
| 43101 | Purine salvage | 8 | 3 | 0.002840 |
| 9259 | Ribonucleotide metabolism | 125 | 12 | 0.003500 |
| 9117 | Nucleotide metabolism | 282 | 21 | 0.003510 |
| 6952 | Defense response | 953 | 54 | 0.003510 |
| 6584 | Catecholamine metabolism | 39 | 6 | 0.003710 |
| 9152 | Purine ribonucleotide biosynthesis | 95 | 10 | 0.003850 |
| 6959 | Humoral immune response | 111 | 11 | 0.003980 |
| 30182 | Neuron differentiation | 480 | 31 | 0.004150 |
| 7409 | Axonogenesis | 232 | 18 | 0.004330 |
| 48676 | Axon extension during development | 3 | 2 | 0.004430 |
| 6601 | Creatine biosynthesis | 3 | 2 | 0.004430 |
| 6244 | Pyrimidine nucleotide catabolism | 3 | 2 | 0.004430 |
| 18958 | Phenol metabolism | 41 | 6 | 0.004790 |
| 51384 | Response to glucocorticoid stimulus | 41 | 6 | 0.004790 |
| 42417 | Dopamine metabolism | 29 | 5 | 0.004820 |
| 43207 | Response to external biotic stimulus | 546 | 34 | 0.004850 |
| 6163 | Purine nucleotide metabolism | 132 | 12 | 0.005430 |
| 8037 | Cell recognition | 19 | 4 | 0.005540 |
| 9260 | Ribonucleotide biosynthesis | 100 | 10 | 0.005550 |
| 9145 | Purine nucleoside triphosphate biosynthesis | 70 | 8 | 0.005700 |
| 9206 | Purine ribonucleoside triphosphate biosynthesis | 70 | 8 | 0.005700 |
| 9201 | Ribonucleoside triphosphate biosynthesis | 70 | 8 | 0.005700 |
| 43094 | Metabolic compound salvage | 10 | 3 | 0.005750 |
| 9142 | Nucleoside triphosphate biosynthesis | 71 | 8 | 0.006210 |
| 9150 | Purine ribonucleotide metabolism | 118 | 11 | 0.006320 |
| 45454 | Cell redox homeostasis | 58 | 7 | 0.007050 |
| 9605 | Response to external stimulus | 991 | 54 | 0.007640 |
| 42069 | Regulation of catecholamine metabolism | 11 | 3 | 0.007680 |
| 6599 | Phosphagen metabolism | 11 | 3 | 0.007680 |
| 8152 | Metabolism | 8550 | 362 | 0.008020 |
| 42775 | ATP synthesis coupled electron transport (sensu Eukaryota) | 33 | 5 | 0.008470 |
| 9116 | Nucleoside metabolism | 33 | 5 | 0.008470 |
| 7412 | Axon target recognition | 4 | 2 | 0.008620 |
| 45063 | T-helper 1 cell differentiation | 4 | 2 | 0.008620 |
| 7406 | Negative regulation of neuroblast proliferation | 4 | 2 | 0.008620 |
| 31571 | G1 DNA damage checkpoint | 4 | 2 | 0.008620 |
| 9205 | Purine ribonucleoside triphosphate metabolism | 91 | 9 | 0.008920 |
| 9199 | Ribonucleoside triphosphate metabolism | 91 | 9 | 0.008920 |
| 42773 | ATP synthesis coupled electron transport | 34 | 5 | 0.009620 |
| 6091 | Generation of precursor metabolites and energy | 786 | 44 | 0.009880 |
| 6118 | Electron transport | 470 | 29 | 0.010000 |
| 5602 | Complement component C1q complex | 3 | 3 | 0.000061 |
| 5737 | Cytoplasm | 4992 | 230 | 0.001740 |
| 30135 | Coated vesicle | 202 | 16 | 0.006260 |
| 30136 | Clathrin-coated vesicle | 173 | 14 | 0.008490 |
| 43020 | NADPH oxidase complex | 4 | 2 | 0.008800 |
| 4133 | Glycogen debranching enzyme activity | 2 | 2 | 0.001550 |
| 4134 | 4-alpha-glucanotransferase activity | 2 | 2 | 0.001550 |
| 4135 | Amylo-alpha-1,6-glucosidase activity | 2 | 2 | 0.001550 |
| 19153 | Protein-disulfide reductase (glutathione) activity | 2 | 2 | 0.001550 |
| 30731 | Guanidinoacetate N-methyltransferase activity | 2 | 2 | 0.001550 |
| 1847 | Opsonin receptor activity | 2 | 2 | 0.001550 |
| 5167 | Neurotrophin TRK receptor binding | 2 | 2 | 0.001550 |
| 5169 | Neurotrophin TRKB receptor binding | 2 | 2 | 0.001550 |
| 3824 | Catalytic activity | 5815 | 263 | 0.002660 |
| 16941 | Natriuretic peptide receptor activity | 3 | 2 | 0.004520 |
| 15271 | Outward rectifier potassium channel activity | 10 | 3 | 0.005920 |
| 43015 | Gamma-tubulin binding | 10 | 3 | 0.005920 |
| 8757 | S-adenosylmethionine-dependent methyltransferase activity | 70 | 8 | 0.006060 |
| 19001 | Guanyl nucleotide binding | 440 | 28 | 0.008740 |
| 4090 | Carbonyl reductase (NADPH) activity | 4 | 2 | 0.008800 |
| 16174 | NAD(P)H oxidase activity | 4 | 2 | 0.008800 |
| 16670 | Oxidoreductase activity, acting on sulfur group of donors, oxygen as acceptor | 4 | 2 | 0.008800 |
| 16167 | Glial cell line-derived neurotrophic factor receptor activity | 4 | 2 | 0.008800 |
| 46923 | ER retention sequence binding | 4 | 2 | 0.008800 |
| 16667 | Oxidoreductase activity, acting on sulfur group of donors | 22 | 4 | 0.009870 |
