# Supplementary material for: Influence of Stochastic Gene Expression on the Cell Survival Rheostat after Traumatic Brain Injury
Source: PLoS One. 2011 Aug 11;6(8):e23111. doi: 10.1371/journal.pone.0023111 (PMC3154935; doi:10.1371/journal.pone.0023111)
Supplement: Table S2 — Group 1: SNCA Homeostasis genes differentially expressed in dying and surviving neurons. (DOC) [file pone.0023111.s010.doc]

**Table S2, Group 1: SNCA Homeostasis genes differentially expressed in dying and surviving neurons.**

| **Accession Number** | **Gene** | **Description** | **Cellular Function** | **Ratio** | **References** |
| --- | --- | --- | --- | --- | --- |
| XM_224614 | BAP1 | BRCA1 associated protein-1 (ubiquitin carboxy-terminal hydrolase) | cytostasis, proliferation, development | 10.230 | [1-2] |
| NM_001005905 | CCT2 | chaperonin containing TCP1, subunit 2 (beta) | growth, protein folding, synaptic plasticity | 5.993 | [3-6] |
| XM_340896 | CDC6 | cell division cycle 6 homolog (S. cerevisiae) | apoptosis, negatively regulates cell proliferation | -7.092 | [7] |
| AI112987 | CDT1 | chromatin licensing and DNA replication factor 1 | cell cycle regulator | -6.494 | [8] |
| NM_017147 | CFL1 | cofilin 1 (non-muscle) | cytoskeletal organization and remodeling | 13.150 | [9-10] |
| NM_053414 | DDX1 | DEAD (Asp-Glu-Ala-Asp) box polypeptide 1 | cell growth, regulation of translation, development | 5.618 | [11-13] |
|  | DUB |  |  | <5 fold |  |
| XM_228753 | HDAC6 | histone deacetylase 6 | regulation of cell cycle and transcription, neuronal plasticity | 5.614 | [14-16] |
| XM_342701 | HERC3 | hect domain and RLD 3 | protein modification and membrane trafficking | 7.180 | [17-18] |
| NM_053842 | MAPK1 | mitogen-activated protein kinase 1 | cell growth, development, survival, signal transduction | 6.982 | [19-22] |
| NM_176079 | MYOD1 | myogenic differentiation 1 | growth, myogenesis, survival | 5.823 | [23] |
|  | Polygalacturonase | |  | <5 fold |  |
| NM_001008289 | SBDS | Shwachman-Bodian-Diamond syndrome | ribosome biogenesis, cell survival | 5.442 | [24-25] |
| NM_134457 | SIAH2 | seven in absentia homolog 2 (Drosophila) | protein catabolism, cell cycle, development | 6.540 | [26-27] |
| XM_001057072 | SKIL | SKI-like oncogene | axonal morphogenesis, proliferation | 7.748 | [28-29] |
| NM_019169 | SNCA | synuclein, alpha (nonA4 component of amyloid precursor) | molecular chaperone, membrane trafficking, cell viability, synaptogenesis, synaptic plasticity | 7.494 | [30-33] |
| XM_215564 | SPG20 | spastic paraplegia 20 (Troyer syndrome) | microtubule dynamics, endosomal trafficking, development | 6.632 | [34-36] |
| XM_228060 | UBASH3A | ubiquitin associated and SH3 domain containing, A | cell regulation, proliferation | 5.219 | [37] |
|  | Ubiquitin | Polyubiquitin, Ub |  | <5 fold |  |
| NM_017237 | UCHL1 | ubiquitin carboxyl-terminal esterase L1 (ubiquitin thiolesterase) | synaptic plasticity, cell homeostasis, development | 5.354 | [38-42] |
| XM_343766 | USP9X | ubiquitin specific peptidase 9, X-linked | synaptic development, self-renewal of neural progenitors | 5.142 | [43-44] |
| XM_218997 | USP47 | ubiquitin specific peptidase 47 | cell growth and survival | 6.876 | [45-46] |
| Ingenuity Pathway Analysis of genes with expression levels greater than five-fold between dying and surviving neurons highlighted seven prominent groups of functionally interconnected genes. Note the remarkable correlation of cell fate with cellular functions (blue color and negative fold changes indicate genes highly expressed in dying neurons, pink color and positive fold changes indicate genes highly expressed in surviving neurons). Ratio is uninjured to injured neurons. | | | | | |
