# Supplementary material for: Influence of Stochastic Gene Expression on the Cell Survival Rheostat after Traumatic Brain Injury
Source: PLoS One. 2011 Aug 11;6(8):e23111. doi: 10.1371/journal.pone.0023111 (PMC3154935; doi:10.1371/journal.pone.0023111)
Supplement: Table S4 — Group 3: BDNF, DRD4, PDCD6IP, cell death control genes differentially expressed in dying and surviving neurons. (DOC) [file pone.0023111.s012.doc]

**Table S4, Group 3: BDNF, DRD4, PDCD6IP, cell death control genes differentially expressed in dying and surviving neurons**.

| **Accession Number** | **Gene** | **Description** | **Cellular Function** | **Ratio** | **References** |
| --- | --- | --- | --- | --- | --- |
| XM_220178 | ADCY9 | adenylate cyclase 9 | cAMP signaling, growth | 8.724 | [61-62] |
|  | Adenylate Cyclase | |  | no change | |
| NM_012513 | BDNF | brain-derived neurotrophic factor | growth, development, survival, plasticity | 6.349 | [63-65] |
| AA925583 | BLNK | B-cell linker | apoptosis, inflammatory response | -15.823 | [66-67] |
| BF400818 | CDC42 | cell division cycle 42 (GTP binding protein, 25kDa) | apoptosis, cell cycle progression | -5.376 | [68] |
| AW915356 | DDEF1 | development and differentiation enhancing factor 1 | cell spreading, morphology | 10.520 | [69-70] |
| NM_012944 | DRD4 | dopamine receptor 4 | dopamine signaling, synaptic plasticity, CNS development | 26.700 | [71-75] |
| NM_019356 | EIF2S1 | eukaryotic translation initiation factor 2, subunit 1 alpha, 35kDa | protein synthesis, survival, glucose homeostasis | 5.469 | [76-78] |
| XM_217250 | EPHB1 | EPH receptor B1 | proliferation, morphogenesis, CNS development, synaptic plasticity | 9.849 | [79-82] |
| NM_001034944 | GRAP2 | GRB2-related adaptor protein 2 | signal transduction, cell-cell signaling | 8.198 | [83] |
| NM_031610 | KCNJ3 | potassium inwardly-rectifying channel, subfamily J, member 3 |  | <5 fold |  |
| NM_017297 | KCNJ5 | potassium inwardly-rectifying channel, subfamily J, member 5 |  | <5 fold |  |
| NM_013192 | KCNJ6 | potassium inwardly-rectifying channel, subfamily J, member 6 | synaptic plasticity, neuronal excitability, brain morphogenesis | 6.885 | [84-86] |
| AA818199 | KHDRBS1 (SAM68) | KH domain containing, RNA binding, signal transduction associated 1 | cell death, cell cycle arrest, RNA splicing, development | -10.929 | [87-91] |
| XM_217246 | NCK1 | NCK adaptor protein 1 | cytoskeletal regulation, cell migration, development | 7.581 | [92-94] |
| XM_001076624 | PDCD6IP (ALIX, AIP1) | programmed cell death 6 interacting protein | apoptosis, cell death regulator, cytokinesis | 7.256 | [95-99] |
| XM_342857 | SH3GL2 (Endophilin) | SH3-domain GRB2-like 2 | synaptic vesicle biogenesis | 8.128 | [100-101] |
| NM_031238 | SH3GL3 (SH3P13) | SH3-domain GRB2-like 3 | synaptic vesicle and membrane biogenesis, vascular development | 8.472 | [102-105] |
| AI179025 | SH3KBP1 (SETA) | SH3-domain kinase binding protein 1 | apoptosis | -9.009 | [106] |
| Ingenuity Pathway Analysis of genes with expression levels greater than five-fold between dying and surviving neurons highlighted seven prominent groups of functionally interconnected genes. Note the remarkable correlation of cell fate with cellular functions (blue color and negative fold changes indicate genes highly expressed in dying neurons, pink color and positive fold changes indicate genes highly expressed in surviving neurons). Ratio is uninjured to injured neurons. | | | | | |
