# Supplementary material for: Influence of Stochastic Gene Expression on the Cell Survival Rheostat after Traumatic Brain Injury
Source: PLoS One. 2011 Aug 11;6(8):e23111. doi: 10.1371/journal.pone.0023111 (PMC3154935; doi:10.1371/journal.pone.0023111)
Supplement: Table S5 — Group 4: BDNF and CREB genes differentially expressed in dying and surviving neurons. (DOC) [file pone.0023111.s013.doc]

**Table S5, Group 4: BDNF and CREB genes differentially expressed in dying and surviving neurons.**

| **Accession Number** | **Gene** | **Description** | | **Cellular Function** | **Ratio** | **References** |
| --- | --- | --- | --- | --- | --- | --- |
| NM_012892 | ACCN1 (ASIC2) | amiloride-sensitive cation channel 1, neuronal (degenerin) | migration, vascular remodeling, morphogenesis, neurotransmission | | 6.558 | [107-108] |
| NM_024154 | ACCN2 (ASIC1A) | amiloride-sensitive cation channel 2, neuronal | acidosis, cell damage | | -5.208 | [109-112] |
| NM_016989 | ADCYAP1 (PACAP38) | adenylate cyclase activating polypeptide 1 (pituitary) | cell-cell signaling, survival, synaptic plasticity, development | | 9.975 | [113-115] |
| NM_019220 | AES(GRG) | amino-terminal enhancer of split | regulation of transcription, development, growth | | 7.498 | [116-118] |
| XM_217411 | ALS2CR8 (CaRF) | amyotrophic lateral sclerosis 2 (juvenile) chromosome region, candidate 8 | transcription regulator | | 6.723 | [119] |
| NM_012513 | BDNF | brain-derived neurotrophic factor | survival, growth, synaptic plasticity, development | | 6.349 | [120-122] |
| NM_012920 | CAMK2A | calcium/calmodulin-dependent protein kinase (CaM kinase) II alpha | long-term potentiation, spatial learning, brain plasticity, development | | 5.311 | [123-125] |
| NM_021739 | CAMK2B | calcium/calmodulin-dependent protein kinase (CaM kinase) II beta | survival, plasticity, synaptic transmission | | 7.719 | [126] |
| XM_343169 | CFD (adipsin) | complement factor D (adipsin) | proteolysis, complement-driven inflammation | | -5.917 | [127-128] |
| NM_134443 | CREB 1 | cAMP responsive element binding protein 1 | survival, development, transcription regulation, synaptic plasticity | | 6.333 | [129-132] |
| XM_226237 | CSNK2A2 | casein kinase 2, alpha prime polypeptide | cell growth, viability, development, cell cycle progression, circadian rhythms | | 5.866 | [133-136] |
| BQ781257 | ETS2 | v-ets erythroblastosis virus E26 oncogene homolog 2 (avian) | apoptosis, regulation of transcription, development | | -5.208 | [137] |
| NM_024360 | HES1 | hairy and enhancer of split 1, (Drosophila) | neural development, fate determination | | 5.549 | [138-140] |
| U92079 | HOMER1 | homer homolog 1 (Drosophila) | glutamate receptor signaling, plasticity, development, sleep regulation | | 5.14 | [141-146] |
| NM_053310 | HOMER3 | homer homolog 3 (Drosophila) | postsynaptic scaffold protein, synaptic plasticity, calcium regulation, growth | | 9.78 | [147-149] |
| NM_134408 | LPHN2 | latrophilin 2 | exocytosis, latrotoxin receptor | | -5.405 | [150-151] |
| NM_053842 | MAPK1 | mitogen-activated protein kinase 1 | survival, transcription regulation, growth, proliferation | | 6.982 | [20] [152-154] |
| XM_573919 | MSR1 | macrophage scavenger receptor 1 | adhesion, apoptosis, phagocytosis | | -14.472 | [155-156] |
|  | NGFB | nerve growth factor, beta polypeptide |  | | <5 fold |  |
| BQ202892 | NOTCH2 | Notch homolog 2 (Drosophila) | apoptosis, cell fate determination, development | | -11.074 | [157] |
| NM_013184 | NTF5 | neurotrophin 5 (neurotrophin 4/5) | growth factor, survival, synaptic plasticity | | 7.601 | [158-159] |
| NM_017232 | PTGS2 (Cox‑2) | prostaglandin-endoperoxide synthase 2 (prostaglandin G/H synthase and cyclooxygenase) | regulation of inflammatory response, survival, mitogenesis, synaptic plasticity | | 6.346 | [160-162] |
| XM_223688 | REL | v-rel reticuloendotheliosis viral oncogene homolog (avian) | development, survival, immune modulation, synaptic plasticity | | 8.07 | [163-166] |
| BQ190001 | SHANK1 | SH3 and multiple ankyrin repeat domains 1 | scaffold protein, synaptic plasticity, development, calcium homeostasis | | -5.848 | [167-170] |
| XM_342851 | TLE1 | transducin-like enhancer of split 1 (E(sp1) homolog, Drosophila) | neuronal development and differentiation | | 5.142 | [171-172] |
| NM_031345 | TSC22D3 (GILZ) | TSC22 domain family, member 3 | anti-inflammatory, immunosuppressive | | 5.224 | [173-175] |
| Ingenuity Pathway Analysis of genes with expression levels greater than five-fold between dying and surviving neurons highlighted seven prominent groups of functionally interconnected genes. Note the remarkable correlation of cell fate with cellular functions (blue color and negative fold changes indicate genes highly expressed in dying neurons, pink color and positive fold changes indicate genes highly expressed in surviving neurons). Ratio is uninjured to injured neurons | | | | | | |
