# Supplementary material for: Influence of Stochastic Gene Expression on the Cell Survival Rheostat after Traumatic Brain Injury
Source: PLoS One. 2011 Aug 11;6(8):e23111. doi: 10.1371/journal.pone.0023111 (PMC3154935; doi:10.1371/journal.pone.0023111)
Supplement: Table S6 — Group 5: IL1β, CASP3 immune response genes differentially expressed in dying and surviving neurons. (DOC) [file pone.0023111.s014.doc]

**Table S6, Group 5: IL1β, CASP3 immune response genes differentially expressed in dying and surviving neurons.**

| **Accession Number** | **Gene** | **Description** | | **Cellular Function** | **Ratio** | **References** |
| --- | --- | --- | --- | --- | --- | --- |
| BI275292 | ANGPT2 | angiopoietin 2 | angiogenesis, apoptosis | | -6.623 | [176-179] |
| AI169021 | CALB1 | calbindin 1, 28kDa | regulation of calcium signaling, apoptosis | | -6.061 | [180-182] |
| AI059604 | CASP3 | caspase 3, apoptosis- related cysteine peptidase | apoptosis, synaptic plasticity | | -10.194 | [183-187] |
| XM_342824 | CCL19 | chemokine (C-C motif) ligand 19 | inflammatory and immune response, immune homeostasis | | -6.803 | [188-190] |
| AA998669 | DIDO1 | death inducer-obliterator 1 | apoptosis, developmental apoptosis | | -5.814 | [191-192] |
| AA817984 | DUSP1 (MKP-1) | dual specificity phosphatase 1 | stress response, apoptosis, synaptic plasticity, regulation of myogenesis | | -6.711 | [193-197] |
| XM_001056128 | GLI1 | glioma-associated oncogene homolog 1 (zinc finger protein) | tumorigenesis, transcription regulation, development | | -6.098 | [198-201] |
| BQ781673 | GRB2 | growth factor receptor-bound protein 2 | cell cycle progression, motility, cell death, development | | -6.369 | [202-206] |
| NM_012583 | HPRT1 | hypoxanthine phosphoribosyltransferase 1 (Lesch-Nyhan syndrome) | regulation of purine metabolism, neuronal development | | 5.324 | [207-208] |
| AI013394 | HS3ST1 | heparan sulfate (glucosamine) 3-O-sulfotransferase 1 | development, anticoagulant | | -5.405 | [209-210] |
| AA859605 | ICAM1 (CD54) | intercellular adhesion molecule 1 (CD54), human rhinovirus receptor | cell adhesion, gatekeeper trafficking molecule | | 9.97 | [211-213] |
|  | IGF1 | insulin-like growth factor 1 (somatomedin C) |  | | no change | |
| AW917764 | IGFBP5 | insulin-like growth factor binding protein 5 | growth, apoptosis, mitogenesis, myogenesis, development | | -11.710 | [214-218] |
| NM_013104 | IGFBP6 | insulin-like growth factor binding protein 6 | apoptosis, negative regulation of proliferation, development | | -7.519 | [219-220] |
| AI044707 | IL1B | interleukin 1, beta | inflammatory and immune response, apoptosis, embryonic development | | -5.917 | [221-225] |
| AA858732 | LYZ | lysozyme (renal amyloidosis) | cytolysis, inflammatory response | | -7.407 | [226-227] |
| XM_220232 | MAPK8IP3 (JSAP1) | mitogen-activated protein kinase 8 interacting protein 3 | proliferation, differentiation, transport, neuritogenesis, embryonic neurogenesis | | 7.699 | [228-231] |
| NM_017322 | MAPK9 (SAPK) | mitogen-activated protein kinase 9 | immune and stress response, growth | | 11.12 | [232-233] |
| NM_031053 | MLH1 | mutL homolog 1, colon cancer, nonpolyposis type 2 (E. coli) | DNA mismatch repair, cell viability | | 6.782 | [234-235] |
| NM_021838 | NOS3 | nitric oxide synthase 3 (endothelial cell) | nitric oxide signaling, hypertrophy | | -5.435 | [138] [236-238] |
| AA925099 | PDGFRA | platelet-derived growth factor receptor, alpha polypeptide | mitogenesis, proliferation, growth | | -7.634 | [239-240] |
| AI555498 | PDGFRB | platelet-derived growth factor receptor, beta polypeptide | mitogenesis, proliferation, growth, development | | -10.593 | [241-243] |
| NM_017139 | PENK | proenkephalin | stress response, pain perception, apoptosis | | -6.098 | [244-246] |
| NM_134367 | PPP3CC | protein phosphatase 3 (formerly 2B), catalytic subunit, gamma isoform | neurodevelopment, synaptic plasticity | | 7.575 | [247-249] |
| NM_017171 | PRKCE | protein kinase C, epsilon | survival, anti-apoptotic, cell-cell signaling | | 8.42 | [250-253] |
| NM_017044 | PTH | parathyroid hormone | apoptosis, calcium and bone homeostasis, fetal mineral homeostasis | | -5.988 | [254-257] |
| CA504956 | PTPN11 (SHP-2) | protein tyrosine phosphatase, non-receptor type 11 (Noonan syndrome 1) | differentiation, mitogenesis, development, apoptosis | | -5.319 | [258-261] |
| NM_012763 | PTPRA (RPTPA, LAR) | protein tyrosine phosphatase, receptor type, A | cell adhesion, proliferation, neuronal migration, neurotrophic signaling, synaptic plasticity, | | 7.563 | [262-264] |
| AA942731 | PTPRD | protein tyrosine phosphatase, receptor type, D | tumor suppressor, synaptic plasticity | | -7.692 | [265-266] |
| AI639318 | RET | ret proto-oncogene | survival, growth, differentiation, development, functional plasticity, apoptosis | | -7.143 | [267-273] |
| XM_342810 | RIPK2 (RIP2) | receptor-interacting serine-threonine kinase 2 | apoptosis, activation-induced cell death, adaptive immune response | | -6.452 | [274-275] |
| AI029930 | SERPINC1 (ANTITHROMBIN) | serpin peptidase inhibitor, clade C (antithrombin), member 1 | anticoagulant | | -5.319 | [276-278] |
| AA943153 | SNAP25 | synaptosomal-associated protein, 25kDa | exocytosis, synaptic transmission and plasticity | | -6.369 | [279-281] |
| AA925792 | SOD1 | superoxide dismutase 1, soluble (amyotrophic lateral sclerosis 1 (adult)) | oxidative stress response, antioxidant, survival, development, synaptic plasticity | | -7.874 | [282-285] |
| AA996732 | TGM2 | transglutaminase 2 (C polypeptide, protein-glutamine-gamma-glutamyltransferase) | stress response, apoptosis | | -7.576 | [286-288] |
| NM_198769 | TLR2 | toll-like receptor 2 | inflammatory immune response, apoptosis | | -5.747 | [289-292] |
| Ingenuity Pathway Analysis of genes with expression levels greater than five-fold between dying and surviving neurons highlighted seven prominent groups of functionally interconnected genes. Note the remarkable correlation of cell fate with cellular functions (blue color and negative fold changes indicate genes highly expressed in dying neurons, pink color and positive fold changes indicate genes highly expressed in surviving neurons). Ratio is uninjured to injured neurons | | | | | | |
