# Supplementary material for: Influence of Stochastic Gene Expression on the Cell Survival Rheostat after Traumatic Brain Injury
Source: PLoS One. 2011 Aug 11;6(8):e23111. doi: 10.1371/journal.pone.0023111 (PMC3154935; doi:10.1371/journal.pone.0023111)
Supplement: Table S7 — Group 6: SOCS Acute Phase genes differentially expressed in dying and surviving neurons. (DOC) [file pone.0023111.s015.doc]

**Table S7, Group 6: SOCS Acute Phase genes differentially expressed in dying and surviving neurons.**

| **Accession Number** | **Gene** | **Description** | | **Cellular Function** | **Ratio** | **References** |
| --- | --- | --- | --- | --- | --- | --- |
| NM_033230 | AKT1 | v-akt murine thymoma viral oncogene homolog 1 |  | | no change | |
| AA996957 | ANXA5 | annexin A5 | apoptosis, membrane trafficking | | -6.098 | [293-295] |
| NM_001007798 | BTK | Bruton agammaglobulinemia tyrosine kinase |  | | no change | |
| AI178828 | EIF4EBP1 | eukaryotic translation initiation factor 4E binding protein 1 | translational repressor, apoptosis, synaptic plasticity | | -6.536 | [296-299] |
| XM_341479 | HABP4 (Ki‑1/57) | hyaluronan binding protein 4 | regulation of transcription | | 7.125 | [300-302] |
| AI501398 | INPP5D (SHIP-1) | inositol polyphosphate-5-phosphatase | apoptosis, cell cycle progression | | -9.709 | [303-304] |
| NM_053466 | JAK1 | Janus kinase 1 (a protein tyrosine kinase) | | | <5 fold |  |
| NM_031514 | JAK2 | Janus kinase 2 (a protein tyrosine kinase) | | | <5 fold |  |
| NM_012855 | JAK3 | Janus kinase 3 (a protein tyrosine kinase, leukocyte) | | | <5 fold |  |
| NM_031020 | MAPK14 | mitogen-activated protein kinase 14 | | | <5 fold |  |
| NM_053847 | MAPK8 | mitogen-activated protein kinase 8 |  | | <5 fold |  |
| BF553322 | MCL1 | myeloid cell leukemia sequence 1 (BCL2-related) | anti-apoptosis, cell homeostasis, survival | | -5.236 | [305-309] |
| AA997941 | NDRG2 | NDRG family member 2 | cell cycle regulation, apoptosis, development | | -6.289 | [310-313] |
| NM_133307 | PRKCD | protein kinase C, delta | apoptosis, gatekeeper | | -5.128 | [314-317] |
| AI171093 | PRKCQ | protein kinase C, theta | apoptosis, immune response | | -6.803 | [318-320] |
| NM_031090 | RAB1A | RAB1A, member RAS oncogene family | membrane trafficking | | 5.275 | [321] |
| NM_053517 | SHC1 (SHCA) | SHC (Src homology 2 domain containing) transforming protein 1 | stress response, proliferation, survival, growth | | 9.129 | [322-323]] |
| NM_058208 | SOCS2 | supressor of cytokine signaling 2 | survival, growth, anti-inflammatory, development | | 22.51 | [324-328] |
| NM_012747 | STAT3 | signal transducer and activator of  transcription 3 (acute-phase response factor) | | | <5 fold |  |
| AI231821 | STMN1 | stathmin 1/oncoprotein 18 | microtubule depolymerization, apoptosis, neuronal plasticity, development | | -5.464 | [329-331] |
| Ingenuity Pathway Analysis of genes with expression levels greater than five-fold between dying and surviving neurons highlighted seven prominent groups of functionally interconnected genes. Note the remarkable correlation of cell fate with cellular functions (blue color and negative fold changes indicate genes highly expressed in dying neurons, pink color and positive fold changes indicate genes highly expressed in surviving neurons). Ratio is uninjured to injured neurons | | | | | | |
