# Supplementary material for: Influence of Stochastic Gene Expression on the Cell Survival Rheostat after Traumatic Brain Injury
Source: PLoS One. 2011 Aug 11;6(8):e23111. doi: 10.1371/journal.pone.0023111 (PMC3154935; doi:10.1371/journal.pone.0023111)
Supplement: Table S8 — Group 7: YY1 Oxidative Stress Response genes differentially expressed in dying and surviving neurons. (DOC) [file pone.0023111.s016.doc]

**Table S8, Group 7: YY1 Oxidative Stress Response genes differentially expressed in dying and surviving neurons.**

| **Accession Number** | **Gene** | **Description** | | **Cellular Function** | **Ratio** | | **References** | | |
| --- | --- | --- | --- | --- | --- | --- | --- | --- | --- |
| NM_019183 | ACTC1 | actin, alpha, cardiac muscle 1 | cardiac function, morphogenesis, cell motility | | 11.04 | [332-334] | | |  |
|  | APC | adenomatosis polyposis coli | proliferation, growth, migration, cell signaling, synaptic plasticity | | 8.536 | [335-337] | | |  |
| XM_342494 | BUB1B | BUB1 budding uninhibited by benzimidazoles 1 homolog beta (yeast) | spindle checkpoint function, cell cycle, oocyte maturation | | -8.264 | [338-341] | | |  |
| AA894004 | CAPG | capping protein (actin filament), gelsolin-like | actin capping, cell motility, phagocytosis | | -6.993 | [342-344] | | |  |
| NM_199502 | CHRDL1 | chordin-like 1 | angiogenesis, differentiation | | -8.772 | [345] | | |  |
| XM_001065606 | CTNND2 (NPRAP) | catenin (cadherin-associated protein), delta 2 (neural plakophilin-related arm-repeat protein) | cell adhesion, cell morphogenesis, cell polarity, cerebral development | | 6.059 | [346-349] | | |  |
| XM_223440 | HMGB3 | high-mobility group box 3 | self-renewal, differentiation | | 5.414 | [350-351] | | |  |
| XM_215184 | INCENP | inner centromere protein antigens 135/155kDa | mitosis, cytokinesis | | 5.516 | [352-353] | | |  |
| XM_232220 | RYBP | RING1 and YY1 binding protein | CNS development, transcriptional repressor, apoptosis | | 9.825 | [354-357] | | |  |
| NM_053822 | S100A8 (MRP8) | S100 calcium binding protein A8 | inflammatory response, apoptosis | | -7.246 | [358-362] | | |  |
| NM_031541 | SCARB1 | scanvenger receptor class b, member 1 | cholesterol metabolism, antiinflammatory, synaptic plasticity | | 6.836 | [156] [363-366] | | |  |
| NM_173290 | YY1 | YY1 transcription factor | transcription regulator, stress response, proliferation, development | | 10.92 | [367-371] | | |  |
| AW142556 | ZNF148 (ZBP-89) | zinc finger protein 148 | transcription regulator, growth, development | | 8.651 | [372-374] | | |  |
| Ingenuity Pathway Analysis of genes with expression levels greater than five-fold between dying and surviving neurons highlighted seven prominent groups of functionally interconnected genes. Note the remarkable correlation of cell fate with cellular functions (blue color and negative fold changes indicate genes highly expressed in dying neurons, pink color and positive fold changes indicate genes highly expressed in surviving neurons). Ratio is uninjured to injured neurons. | | | | | | | |  | |
