# Supplementary material for: Influence of Stochastic Gene Expression on the Cell Survival Rheostat after Traumatic Brain Injury
Source: PLoS One. 2011 Aug 11;6(8):e23111. doi: 10.1371/journal.pone.0023111 (PMC3154935; doi:10.1371/journal.pone.0023111)
Supplement: Table S9 — Probe and primer sequences for qPCR. (DOC) [file pone.0023111.s017.doc]

| **Table S9. Probe and primer sequences for qPCR.** | | | | | |
| --- | --- | --- | --- | --- | --- |
| **Gene** | **Accession #** | **Probe Sequence** | **Sense Primer** | **Anti-sense Primer** | **Dye** |
| Cebpa | NM_012524 | CCAACACCTTCTGCTGCGTCTCCA | GGATAAGAACAGCAACGAGTACC | CGGTCATTGTCACTGGTCAAC | FAM |
| IL16R | NM_017020 | CCAAGCAACCAGGCAAGCCAGGCA | GCTTCACTGCTGGAATTGGC | TAAAGAACTGCTAGGAAACTCTGC | ROX |
| Casp9 | NM_031632 | TTCCTCAGGGCTCAGCACACGCAT | GCCTCATCATCAACAACGTGAAC | TCTTAGCAGTCAGGTCGTTCTTC | FAM |
| Bid | NM_022684 | ACGTCTTCCGCACGACCGTGAACT | GGCCAGTCATGCACCATCTC | TTTCTAACCAAGTCCCTCACGTAG | CY5 |
| C-Jun | X17163 | TACGCTGCCCAGTGTCACCTCCGC | GCCCTAGCTGAACTGCATAGC | CCAGCTACTGAGGCCACAGC | ROX |
| HSP70 | Z27118 | TCCCCACCAAGCAGACGCAGACCT | CTCATCAAGCGCAACTCCAC | CCTCGTACACCTGGATCAGC | Cy5 |
| GPX | NM_030826 | ACCGAGCACCACCAGGCCACGA | ACTACACCGAAATGAATGATCTGC | TCCTGATGTCCGAACTGATTGC | ROX |
| BDNF | NM_12513 | ACTCCGACCCCGCCCGCCGT | TGCCGCAAACATGTCTATGAGG | CCCACTCGCTAATACTGTCACAC | FAM |
| TAC | NM_012666.1 | AGCCGCCTGCACTGCCTCACA | GTACAAGTTTGCCAGCGATGC | AGACACACAGGAGTTTCTCTGC | CY5 |
| PIAS2 | NM_053337.1 | ATGAGGCTGACAATCCCGTGCCGC | TGTCCTTGATGTGCCCTTTAGG | AAATCCAGGTGGGCTTCTTCTC | ROX |
| MTF1 | XM_342910 | ATGACGGCAGGCAGCATCACACCC | GCAGGGGTAGAGGAAAGAGC | TCTTCATCCTTCCAATTTCTGACC | FAM |
| JUNB | NM_021836 | CCGCCAGCCTGTTCCGCAGCC | GAGGAACCGCAGACCGTACC | GAGGAACCGCAGACCGTACC | ROX |
| nNOS | U67309 | CGGAGAAGGACCAGTCCACCACGG | GCCATCGTCTCCTACCACAC | CATCGTCAGCCTGTATTCTGTTG | FAM |
| BAX | NM_017059 | CATCCTGGGGCGGCTGCTCCAA | CCCACCAGCTCTGAACAGTTC | ACACTCGCTCAGCTTCTTGG | CY5 |
| iNOS | NM_012611 | TTGCGTGTGCCTGCTGCCTTCCTG | CTGGAGGTGCTGGAAGAGTTC | TGGGAGGAGCTGATGGAGTAG | ROX |
| HO | M12129 | ATGCCCCGCTCTACTTCCCTGAGG | GAGCGAAACAAGCAGAACCC | GGCTGGTGTGTAAGGGATGG | FAM |
| CD14 | NM_021744 | AGCCAAAGTTCCTGACAAGCCCGC | GAGGCGTATAACTCTGGCGTAG | CCTTAAACAAAGAGGCGATGTCC | CY5 |
| SOS2 | XM_001080400 | CCTCGCCACGGATTCCACGCAGAT | CCCTAATTCACCAAGCACTCCTC | GTGAGCAAGATTGTTGTGACTGG | ROX |
| NPY1r | NM_001013032 | TGTTCCTGCTCTGCCACCTCACGG | TGCCACCTGCAACCACAATC | AAGAAGAACTGCAAGTCTCTCTGG | FAM |
| IGFBP3 | NM_012588.1 | TTGTCCACGCACCAGCAGAAGCCC | AAGAAACAGTGTCGCCCTTCC | GGTGTCATAGCCTGGCAATGG | CY5 |
| MMP9 | NM_031055.1 | AGCAGAGCCTTCCCTCCACGACGC | TCAGAGGTAACCCTGGTCACC | AGTCGAATTTCCAGATACGTTCCC | ROX |
| NPY | NM_012614.1 | AGCGAGGGTCAGTCCACACAGCC | GCCATGATGCTAGGTAACAAACG | CAGAATGCCCAAACACACGAG | FAM |
| NFYB | NM_031553 | TCACCGCTCCTCCGCTCACTTGCT | GAAAGGTCAGGTCCCTGTCG | ATTCCAAGTGCTGGCTGCTC | CY5 |
| RUNX1 | NM_017325 | CTACCTGCCGCCGCCCTACCCA | GCTCGACCTCTCGCTACCAC | CCGAGGTGCCGTAGTACAGG | ROX |
| JAK3 | NM_012855.1 | AGCCAGGTCACGATGCACGCAGC | CTGCAAGGGCATGGAGTACC | CTCACTCTCCACCAGGATGTTG | FAM |
| NPR1 | NM_012613.1 | CGCTCCTTCCGCATCCGCCATAGG | ATGGCACTTGCACTACTGGATG | GCATCTTTAGCCCTACCACACC | CY5 |
| SOD1 | NM_012613.1 | CCACACCGTCCTTTCCAGCAGCCA | AAGAGAGGCATGTTGGAGACC | ATCACACGATCTTCAATGGACAC | ROX |
| GAL | NM_033237.1 | AGGCAGGGGCACAGCAACACTTCC | CCACAGATCATTTAGCGACAAGC | CCATTATAGTGCGGACGATATTGC | FAM |
| GAPDH | NM_017008 | ATCTTCCAGGAGCGCGATCCCG | AAGCTGGTCATCAATGGGAAAC | GAAGACGCCAGTAGACTCCACG | HEX |
| Cebpa, CCAAT/enhancer binding protein alpha; Casp9, Caspase 9; Bid, BH3 interacting domain death agonist; HSP70, Heat Shock Protein 70; GPX-1, Glutathione Peroxidase 1; BDNF, Brain Dervived Neutrophic Factor; TAC1, Tachykinin 1; PIAS2, Protein inhibitor of activated STAT, 2; MTF1, Metal response element binding transcription factor 1; nNos, Neuronal Nitric Oxide Synthase; BAX , Bcl2-associated X protein; iNOS, nitric oxide synthase 2; HO-1, Heme oxygenase-1; CD14, CD14 molecule; SOS2, Son of Sevenless homolog 2; NPY1r, Neuropeptide Y receptor Y1; IGFBP3, Insulin-like growth factor binding protein 3; MMP9, Matrix Metallopeptidase 9; NPY, Neuropeptide Y; NFYB, Nuclear Transcription Factor-Y beta; RUNX1, Runt Related transcription factor 1; JAK3, Janus kinase 3; NPR1, Atrionatriuretic Peptide Receptor A; SOD1, Superoxide Dismutase 1 | | | | | |
