# Supplementary material for: Influence of Stochastic Gene Expression on the Cell Survival Rheostat after Traumatic Brain Injury
Source: PLoS One. 2011 Aug 11;6(8):e23111. doi: 10.1371/journal.pone.0023111 (PMC3154935; doi:10.1371/journal.pone.0023111)
Supplement: References S1 — Supplementary References. (DOC) [file pone.0023111.s018.doc]

Supplemental Reference List

1. Misaghi S, Ottosen S, Izrael-Tomasevic A, Arnott D, Lamkanfi M et al. (2009) Association of C-terminal ubiquitin hydrolase BRCA1-associated protein 1 with cell cycle regulator host cell factor 1. Mol Cell Biol 29: 2181-2192.

2. Tse WK, Eisenhaber B, Ho SH, Ng Q, Eisenhaber F et al. (2009) Genome-wide loss-of-function analysis of deubiquitylating enzymes for zebrafish development. BMC Genomics 10: 637.

3. Dekker C, Stirling PC, McCormack EA, Filmore H, Paul A et al. (2008) The interaction network of the chaperonin CCT. EMBO J 27: 1827-1839.

4. Yu JH, Seo JY, Kim KH, Kim H (2008) Differentially expressed proteins in cerulein-stimulated pancreatic acinar cells: implication for acute pancreatitis. Int J Biochem Cell Biol 40: 503-516.

5. Ding Q, Vaynman S, Souda P, Whitelegge JP, Gomez-Pinilla F (2006) Exercise affects energy metabolism and neural plasticity-related proteins in the hippocampus as revealed by proteomic analysis. Eur J Neurosci 24: 1265-1276.

6. Schuller E, Gulesserian T, Seidl R, Cairns N, Lube G (2001) Brain t-complex polypeptide 1 (TCP- 1) related to its natural substrate beta1 tubulin is decreased in Alzheimer's disease. Life Sci 69: 263-270.

7. Takabatake N, Toriyama S, Igarashi A, Tokairin Y, Takeishi Y et al. (2009) A novel polymorphism in CDC6 is associated with the decline in lung function of ex-smokers in COPD. Biochem Biophys Res Commun 381: 554-559.

8. Tada S (2007) Cdt1 and geminin: role during cell cycle progression and DNA damage in higher eukaryotes. Front Biosci 12: 1629-1641.

9. Chae JI, Kim J, Woo SM, Han HW, Cho YK et al. (2009) Cytoskeleton-associated proteins are enriched in human embryonic-stem cell-derived neuroectodermal spheres. Proteomics 9: 1128-1141.

10. Willis D, Li KW, Zheng JQ, Chang JH, Smit A et al. (2005) Differential transport and local translation of cytoskeletal, injury-response, and neurodegeneration protein mRNAs in axons. J Neurosci 25: 778-791.

11. Godbout R, Packer M, Katyal S, Bleoo S (2002) Cloning and expression analysis of the chicken DEAD box gene DDX1. Biochim Biophys Acta 1574: 63-71.

12. Kircher SG, Kim SH, Fountoulakis M, Lubec G (2002) Reduced levels of DEAD-box proteins DBP-RB and p72 in fetal Down syndrome brains. Neurochem Res 27: 1141-1146.

13. Sato N, Sugimura Y, Hayashi Y, Murase T, Kanou Y et al. (2008) Identification of genes differentially expressed in mouse fetuses from streptozotocin-induced diabetic pregnancy by cDNA subtraction. Endocr J 55: 317-323.

14. Ajamian F, Suuronen T, Salminen A, Reeben M (2003) Upregulation of class II histone deacetylases mRNA during neural differentiation of cultured rat hippocampal progenitor cells. Neurosci Lett 346: 57-60.

15. Pandey UB, Nie Z, Batlevi Y, McCray BA, Ritson GP et al. (2007) HDAC6 rescues neurodegeneration and provides an essential link between autophagy and the UPS. Nature 447: 859-863.

16. Kim AH, Puram SV, Bilimoria PM, Ikeuchi Y, Keough S et al. (2009) A centrosomal Cdc20-APC pathway controls dendrite morphogenesis in postmitotic neurons. Cell 136: 322-336.

17. Garcia-Gonzalo FR, Rosa JL (2005) The HERC proteins: functional and evolutionary insights. Cell Mol Life Sci 62: 1826-1838.

18. Davies W, Smith RJ, Kelsey G, Wilkinson LS (2004) Expression patterns of the novel imprinted genes Nap1l5 and Peg13 and their non-imprinted host genes in the adult mouse brain. Gene Expr Patterns 4: 741-747.

19. Lu Z, Xu S (2006) ERK1/2 MAP kinases in cell survival and apoptosis. IUBMB Life 58: 621-631.

20. Jia J, Chen X, Zhu W, Luo Y, Hua Z et al. (2008) CART protects brain from damage through ERK activation in ischemic stroke. Neuropeptides 42: 653-661.

21. Kim SJ, Lee K (2008) Extracts of Liriopsis tuber protect AMPA induced brain damage and improve memory with the activation of insulin receptor and ERK I/II. Phytother Res 22: 1450-1457.

22. Tomaselli B, Nedden SZ, Podhraski V, Baier-Bitterlich G (2008) p42/44 MAPK is an essential effector for purine nucleoside-mediated neuroprotection of hypoxic PC12 cells and primary cerebellar granule neurons. Mol Cell Neurosci 38: 559-568.

23. Goetsch SC, Hawke TJ, Gallardo TD, Richardson JA, Garry DJ (2003) Transcriptional profiling and regulation of the extracellular matrix during muscle regeneration. Physiol Genomics 14: 261-271.

24. Rujkijyanont P, Watanabe K, Ambekar C, Wang H, Schimmer A et al. (2008) SBDS-deficient cells undergo accelerated apoptosis through the Fas-pathway. Haematologica 93: 363-371.

25. Hesling C, Oliveira CC, Castilho BA, Zanchin NI (2007) The Shwachman-Bodian-Diamond syndrome associated protein interacts with HsNip7 and its down-regulation affects gene expression at the transcriptional and translational levels. Exp Cell Res 313: 4180-4195.

26. Carlucci A, Adornetto A, Scorziello A, Viggiano D, Foca M et al. (2008) Proteolysis of AKAP121 regulates mitochondrial activity during cellular hypoxia and brain ischaemia. EMBO J 27: 1073-1084.

27. Hu G, Chung YL, Glover T, Valentine V, Look AT et al. (1997) Characterization of human homologs of the Drosophila seven in absentia (sina) gene. Genomics 46: 103-111.

28. Stegmuller J, Konishi Y, Huynh MA, Yuan Z, Dibacco S et al. (2006) Cell-intrinsic regulation of axonal morphogenesis by the Cdh1-APC target SnoN. Neuron 50: 389-400.

29. Pearson-White S, Crittenden R (1997) Proto-oncogene Sno expression, alternative isoforms and immediate early serum response. Nucleic Acids Res 25: 2930-2937.

30. Chandra S, Gallardo G, Fernandez-Chacon R, Schluter OM, Sudhof TC (2005) Alpha-synuclein cooperates with CSPalpha in preventing neurodegeneration. Cell 123: 383-396.

31. Zhong SC, Luo X, Chen XS, Cai QY, Liu J et al. (2009) Expression and Subcellular Location of Alpha-Synuclein During Mouse-Embryonic Development. Cell Mol Neurobiol .

32. Mladenovic DA, Perovic M, Tesic V, Tanic N, Rakic L et al. (2010) Long-term dietary restriction modulates the level of presynaptic proteins in the cortex and hippocampus of the aging rat. Neurochem Int 56: 250-255.

33. Hashimoto M, Hsu LJ, Rockenstein E, Takenouchi T, Mallory M et al. (2002) alpha-Synuclein protects against oxidative stress via inactivation of the c-Jun N-terminal kinase stress-signaling pathway in neuronal cells. J Biol Chem 277: 11465-11472.

34. Bakowska JC, Jenkins R, Pendleton J, Blackstone C (2005) The Troyer syndrome (SPG20) protein spartin interacts with Eps15. Biochem Biophys Res Commun 334: 1042-1048.

35. Burgunder JM, Hunziker W (2003) Hereditary spastic paraplegia: clues from a rare disorder for a common problem? IUBMB Life 55: 347-352.

36. Patel H, Cross H, Proukakis C, Hershberger R, Bork P et al. (2002) SPG20 is mutated in Troyer syndrome, an hereditary spastic paraplegia. Nat Genet 31: 347-348.

37. Tsygankov AY (2008) Multidomain STS/TULA proteins are novel cellular regulators. IUBMB Life 60: 224-231.

38. Sakurai M, Sekiguchi M, Zushida K, Yamada K, Nagamine S et al. (2008) Reduction in memory in passive avoidance learning, exploratory behaviour and synaptic plasticity in mice with a spontaneous deletion in the ubiquitin C-terminal hydrolase L1 gene. Eur J Neurosci 27: 691-701.

39. Dodge R, Loomans C, Sharma A, Bonner-Weir S (2009) Developmental pathways during in vitro progression of human islet neogenesis. Differentiation 77: 135-147.

40. Shen H, Sikorska M, Leblanc J, Walker PR, Liu QY (2006) Oxidative stress regulated expression of ubiquitin Carboxyl-terminal Hydrolase-L1: role in cell survival. Apoptosis 11: 1049-1059.

41. Bonin M, Poths S, Osaka H, Wang YL, Wada K et al. (2004) Microarray expression analysis of gad mice implicates involvement of Parkinson's disease associated UCH-L1 in multiple metabolic pathways. Brain Res Mol Brain Res 126: 88-97.

42. Walters BJ, Campbell SL, Chen PC, Taylor AP, Schroeder DG et al. (2008) Differential effects of Usp14 and Uch-L1 on the ubiquitin proteasome system and synaptic activity. Mol Cell Neurosci 39: 539-548.

43. Xu J, Taya S, Kaibuchi K, Arnold AP (2005) Spatially and temporally specific expression in mouse hippocampus of Usp9x, a ubiquitin-specific protease involved in synaptic development. J Neurosci Res 80: 47-55.

44. Jolly LA, Taylor V, Wood SA (2009) USP9X enhances the polarity and self-renewal of embryonic stem cell-derived neural progenitors. Mol Biol Cell 20: 2015-2029.

45. Xu J (2005) Age-related changes in Usp9x protein expression and DNA methylation in mouse brain. Brain Res Mol Brain Res 140: 17-24.

46. Peschiaroli A, Skaar JR, Pagano M, Melino G (2010) The ubiquitin-specific protease USP47 is a novel beta-TRCP interactor regulating cell survival. Oncogene 29: 1384-1393.

47. Bhargava A, Meijer OC, Dallman MF, Pearce D (2000) Plasma membrane calcium pump isoform 1 gene expression is repressed by corticosterone and stress in rat hippocampus. J Neurosci 20: 3129-3138.

48. Renteria RC, Strehler EE, Copenhagen DR, Krizaj D (2005) Ontogeny of plasma membrane Ca2+ ATPase isoforms in the neural retina of the postnatal rat. Vis Neurosci 22: 263-274.

49. Tempel BL, Shilling DJ (2007) The plasma membrane calcium ATPase and disease. Subcell Biochem 45: 365-383.

50. Matozaki T, Murata Y, Okazawa H, Ohnishi H (2009) Functions and molecular mechanisms of the CD47-SIRPalpha signalling pathway. Trends Cell Biol 19: 72-80.

51. Murata T, Ohnishi H, Okazawa H, Murata Y, Kusakari S et al. (2006) CD47 promotes neuronal development through Src- and FRG/Vav2-mediated activation of Rac and Cdc42. J Neurosci 26: 12397-12407.

52. Stahel PF, Flierl MA, Morgan BP, Persigehl I, Stoll C et al. (2009) Absence of the complement regulatory molecule CD59a leads to exacerbated neuropathology after traumatic brain injury in mice. J Neuroinflammation 6: 2.

53. Ramlawi B, Otu H, Rudolph JL, Mieno S, Kohane IS et al. (2007) Genomic expression pathways associated with brain injury after cardiopulmonary bypass. J Thorac Cardiovasc Surg 134: 996-1005.

54. Ke ZJ, Bowen WM, Gibson GE (2006) Peripheral inflammatory mechanisms modulate microglial activation in response to mild impairment of oxidative metabolism. Neurochem Int 49: 548-556.

55. Daniel C, Amann K, Hohenstein B, Bornstein P, Hugo C (2007) Thrombospondin 2 functions as an endogenous regulator of angiogenesis and inflammation in experimental glomerulonephritis in mice. J Am Soc Nephrol 18: 788-798.

56. Xue M, Del Bigio MR (2001) Acute tissue damage after injections of thrombin and plasmin into rat striatum. Stroke 32: 2164-2169.

57. Vawter MP, Ferran E, Galke B, Cooper K, Bunney WE et al. (2004) Microarray screening of lymphocyte gene expression differences in a multiplex schizophrenia pedigree. Schizophr Res 67: 41-52.

58. Murphy JA, Franklin TB, Rafuse VF, Clarke DB (2007) The neural cell adhesion molecule is necessary for normal adult retinal ganglion cell number and survival. Mol Cell Neurosci 36: 280-292.

59. Sieber FE, Traystman RJ, Martin LJ (1997) Delayed neuronal death after global incomplete ischemia in dogs is accompanied by changes in phospholipase C protein expression. J Cereb Blood Flow Metab 17: 527-533.

60. Choi SY, Chang J, Jiang B, Seol GH, Min SS et al. (2005) Multiple receptors coupled to phospholipase C gate long-term depression in visual cortex. J Neurosci 25: 11433-11443.

61. Toyota T, Hattori E, Meerabux J, Yamada K, Saito K et al. (2002) Molecular analysis, mutation screening, and association study of adenylate cyclase type 9 gene (ADCY9) in mood disorders. Am J Med Genet 114: 84-92.

62. Cumbay MG, Watts VJ (2004) Novel regulatory properties of human type 9 adenylate cyclase. J Pharmacol Exp Ther 310: 108-115.

63. Kharebava G, Makonchuk D, Kalita KB, Zheng JJ, Hetman M (2008) Requirement of 3-phosphoinositide-dependent protein kinase-1 for BDNF-mediated neuronal survival. J Neurosci 28: 11409-11420.

64. Rogoz Z, Skuza G, Legutko B (2008) Repeated co-treatment with fluoxetine and amantadine induces brain-derived neurotrophic factor gene expression in rats. Pharmacol Rep 60: 817-826.

65. Ploughman M, Windle V, Maclellan CL, White N, Dore JJ et al. (2009) Brain-derived neurotrophic factor contributes to recovery of skilled reaching after focal ischemia in rats. Stroke 40: 1490-1495.

66. Han W, Takano T, He J, Ding J, Gao S et al. (2001) Role of BLNK in oxidative stress signaling in B cells. Antioxid Redox Signal 3: 1065-1073.

67. Mizuno K, Tagawa Y, Mitomo K, Watanabe N, Katagiri T et al. (2002) Src homology region 2 domain-containing phosphatase 1 positively regulates B cell receptor-induced apoptosis by modulating association of B cell linker protein with Nck and activation of c-Jun NH2-terminal kinase. J Immunol 169: 778-786.

68. Zhao J, Pei DS, Zhang QG, Zhang GY (2007) Down-regulation Cdc42 attenuates neuronal apoptosis through inhibiting MLK3/JNK3 cascade during ischemic reperfusion in rat hippocampus. Cell Signal 19: 831-843.

69. Matsui C, Kaieda S, Ikegami T, Mimori-Kiyosue Y (2008) Identification of a link between the SAMP repeats of adenomatous polyposis coli tumor suppressor and the Src homology 3 domain of DDEF. J Biol Chem 283: 33006-33020.

70. Miyata M, Raven JF, Baltzis D, Koromilas AE, Sabe H (2008) IRES-mediated translational control of AMAP1 expression during differentiation of monocyte U937 cells. Cell Cycle 7: 3273-3281.

71. Wong AH, Van Tol HH (2003) The dopamine D4 receptors and mechanisms of antipsychotic atypicality. Prog Neuropsychopharmacol Biol Psychiatry 27: 1091-1099.

72. Yuen EY, Yan Z (2009) Dopamine D4 receptors regulate AMPA receptor trafficking and glutamatergic transmission in GABAergic interneurons of prefrontal cortex. J Neurosci 29: 550-562.

73. Oak JN, Oldenhof J, Van Tol HH (2000) The dopamine D(4) receptor: one decade of research. Eur J Pharmacol 405: 303-327.

74. Baimoukhametova DV, Hewitt SA, Sank CA, Bains JS (2004) Dopamine modulates use-dependent plasticity of inhibitory synapses. J Neurosci 24: 5162-5171.

75. Holmboe K, Nemoda Z, Fearon RM, Csibra G, Sasvari-Szekely M et al. (2010) Polymorphisms in dopamine system genes are associated with individual differences in attention in infancy. Dev Psychol 46: 404-416.

76. Petrov T, Underwood BD, Braun B, Alousi SS, Rafols JA (2001) Upregulation of iNOS expression and phosphorylation of eIF-2alpha are paralleled by suppression of protein synthesis in rat hypothalamus in a closed head trauma model. J Neurotrauma 18: 799-812.

77. Wyrwicz LS, Gaj P, Hoffmann M, Rychlewski L, Ostrowski J (2007) A common cis-element in promoters of protein synthesis and cell cycle genes. Acta Biochim Pol 54: 89-98.

78. Scheuner D, Vander MD, Song B, Flamez D, Creemers JW et al. (2005) Control of mRNA translation preserves endoplasmic reticulum function in beta cells and maintains glucose homeostasis. Nat Med 11: 757-764.

79. Biervert C, Horvath E, Fahrig T (2001) Semiquantitative expression analysis of ephrine-receptor tyrosine kinase mRNA's in a rat model of traumatic brain injury. Neurosci Lett 315: 25-28.

80. Chumley MJ, Catchpole T, Silvany RE, Kernie SG, Henkemeyer M (2007) EphB receptors regulate stem/progenitor cell proliferation, migration, and polarity during hippocampal neurogenesis. J Neurosci 27: 13481-13490.

81. Petros TJ, Shrestha BR, Mason C (2009) Specificity and sufficiency of EphB1 in driving the ipsilateral retinal projection. J Neurosci 29: 3463-3474.

82. Liu WT, Han Y, Li HC, Adams B, Zheng JH et al. (2009) An in vivo mouse model of long-term potentiation at synapses between primary afferent C-fibers and spinal dorsal horn neurons: essential role of EphB1 receptor. Mol Pain 5: 29.

83. Birge RB, Knudsen BS, Besser D, Hanafusa H (1996) SH2 and SH3-containing adaptor proteins: redundant or independent mediators of intracellular signal transduction. Genes Cells 1: 595-613.

84. Saenz del BL, Cortes R, Mengod G, Zarate J, Echevarria E et al. (2008) Distribution and neurochemical characterization of neurons expressing GIRK channels in the rat brain. J Comp Neurol 510: 581-606.

85. Chung HJ, Ge WP, Qian X, Wiser O, Jan YN et al. (2009) G protein-activated inwardly rectifying potassium channels mediate depotentiation of long-term potentiation. Proc Natl Acad Sci U S A 106: 635-640.

86. Yao G, Chen XN, Flores-Sarnat L, Barlow GM, Palka G et al. (2006) Deletion of chromosome 21 disturbs human brain morphogenesis. Genet Med 8: 1-7.

87. Babic I, Cherry E, Fujita DJ (2006) SUMO modification of Sam68 enhances its ability to repress cyclin D1 expression and inhibits its ability to induce apoptosis. Oncogene 25: 4955-4964.

88. Paronetto MP, Achsel T, Massiello A, Chalfant CE, Sette C (2007) The RNA-binding protein Sam68 modulates the alternative splicing of Bcl-x. J Cell Biol 176: 929-939.

89. Polotskaia A, Wang M, Patschan S, Addabbo F, Chen J et al. (2007) Regulation of arginine methylation in endothelial cells: role in premature senescence and apoptosis. Cell Cycle 6: 2524-2530.

90. Lim DA, Suarez-Farinas M, Naef F, Hacker CR, Menn B et al. (2006) In vivo transcriptional profile analysis reveals RNA splicing and chromatin remodeling as prominent processes for adult neurogenesis. Mol Cell Neurosci 31: 131-148.

91. Paronetto MP, Bianchi E, Geremia R, Sette C (2008) Dynamic expression of the RNA-binding protein Sam68 during mouse pre-implantation development. Gene Expr Patterns 8: 311-322.

92. Fawcett JP, Georgiou J, Ruston J, Bladt F, Sherman A et al. (2007) Nck adaptor proteins control the organization of neuronal circuits important for walking. Proc Natl Acad Sci U S A 104: 20973-20978.

93. Ruusala A, Pawson T, Heldin CH, Aspenstrom P (2008) Nck adapters are involved in the formation of dorsal ruffles, cell migration, and Rho signaling downstream of the platelet-derived growth factor beta receptor. J Biol Chem 283: 30034-30044.

94. Bladt F, Aippersbach E, Gelkop S, Strasser GA, Nash P et al. (2003) The murine Nck SH2/SH3 adaptors are important for the development of mesoderm-derived embryonic structures and for regulating the cellular actin network. Mol Cell Biol 23: 4586-4597.

95. Trioulier Y, Torch S, Blot B, Cristina N, Chatellard-Causse C et al. (2004) Alix, a protein regulating endosomal trafficking, is involved in neuronal death. J Biol Chem 279: 2046-2052.

96. Mahul-Mellier AL, Strappazzon F, Petiot A, Chatellard-Causse C, Torch S et al. (2008) Alix and ALG-2 are involved in tumor necrosis factor receptor 1-induced cell death. J Biol Chem 283: 34954-34965.

97. Zhan L, Liu B, Jose-Lafuente M, Chibalina MV, Grierson A et al. (2008) ALG-2 interacting protein AIP1: a novel link between D1 and D3 signalling. Eur J Neurosci 27: 1626-1633.

98. Pan S, Wang R, Zhou X, He G, Koomen J et al. (2006) Involvement of the conserved adaptor protein Alix in actin cytoskeleton assembly. J Biol Chem 281: 34640-34650.

99. Krebs J, Klemenz R (2000) The ALG-2/AIP-complex, a modulator at the interface between cell proliferation and cell death? A hypothesis. Biochim Biophys Acta 1498: 153-161.

100. Schuske KR, Richmond JE, Matthies DS, Davis WS, Runz S et al. (2003) Endophilin is required for synaptic vesicle endocytosis by localizing synaptojanin. Neuron 40: 749-762.

101. Reutens AT, Begley CG (2002) Endophilin-1: a multifunctional protein. Int J Biochem Cell Biol 34: 1173-1177.

102. Gould RM, Freund CM, Palmer F, Feinstein DL (2000) Messenger RNAs located in myelin sheath assembly sites. J Neurochem 75: 1834-1844.

103. Ringstad N, Nemoto Y, De CP (1997) The SH3p4/Sh3p8/SH3p13 protein family: binding partners for synaptojanin and dynamin via a Grb2-like Src homology 3 domain. Proc Natl Acad Sci U S A 94: 8569-8574.

104. Sittler A, Walter S, Wedemeyer N, Hasenbank R, Scherzinger E et al. (1998) SH3GL3 associates with the Huntingtin exon 1 protein and promotes the formation of polygln-containing protein aggregates. Mol Cell 2: 427-436.

105. Gomez GA, Veldman MB, Zhao Y, Burgess S, Lin S (2009) Discovery and characterization of novel vascular and hematopoietic genes downstream of etsrp in zebrafish. PLoS One 4: e4994.

106. Chen B, Borinstein SC, Gillis J, Sykes VW, Bogler O (2000) The glioma-associated protein SETA interacts with AIP1/Alix and ALG-2 and modulates apoptosis in astrocytes. J Biol Chem 275: 19275-19281.

107. Grifoni SC, Jernigan NL, Hamilton G, Drummond HA (2008) ASIC proteins regulate smooth muscle cell migration. Microvasc Res 75: 202-210.

108. Grifoni SC, McKey SE, Drummond HA (2008) Hsc70 regulates cell surface ASIC2 expression and vascular smooth muscle cell migration. Am J Physiol Heart Circ Physiol 294: H2022-H2030.

109. Arias RL, Sung ML, Vasylyev D, Zhang MY, Albinson K et al. (2008) Amiloride is neuroprotective in an MPTP model of Parkinson's disease. Neurobiol Dis 31: 334-341.

110. Xiong ZG, Zhu XM, Chu XP, Minami M, Hey J et al. (2004) Neuroprotection in ischemia: blocking calcium-permeable acid-sensing ion channels. Cell 118: 687-698.

111. Simon R, Xiong Z (2006) Acidotoxicity in brain ischaemia. Biochem Soc Trans 34: 1356-1361.

112. Pignataro G, Simon RP, Xiong ZG (2007) Prolonged activation of ASIC1a and the time window for neuroprotection in cerebral ischaemia. Brain 130: 151-158.

113. Suk K, Park JH, Lee WH (2004) Neuropeptide PACAP inhibits hypoxic activation of brain microglia: a protective mechanism against microglial neurotoxicity in ischemia. Brain Res 1026: 151-156.

114. Frechilla D, Garcia-Osta A, Palacios S, Cenarruzabeitia E, Del RJ (2001) BDNF mediates the neuroprotective effect of PACAP-38 on rat cortical neurons. Neuroreport 12: 919-923.

115. Li M, David C, Kikuta T, Somogyvari-Vigh A, Arimura A (2005) Signaling cascades involved in neuroprotection by subpicomolar pituitary adenylate cyclase-activating polypeptide 38. J Mol Neurosci 27: 91-105.

116. Jennings BH, Ish-Horowicz D (2008) The Groucho/TLE/Grg family of transcriptional co-repressors. Genome Biol 9: 205.

117. Yu X, Li P, Roeder RG, Wang Z (2001) Inhibition of androgen receptor-mediated transcription by amino-terminal enhancer of split. Mol Cell Biol 21: 4614-4625.

118. Tetsuka T, Uranishi H, Imai H, Ono T, Sonta S et al. (2000) Inhibition of nuclear factor-kappaB-mediated transcription by association with the amino-terminal enhancer of split, a Groucho-related protein lacking WD40 repeats. J Biol Chem 275: 4383-4390.

119. Tao X, West AE, Chen WG, Corfas G, Greenberg ME (2002) A calcium-responsive transcription factor, CaRF, that regulates neuronal activity-dependent expression of BDNF. Neuron 33: 383-395.

120. Wu H, Lu D, Jiang H, Xiong Y, Qu C et al. (2008) Simvastatin-Mediated Upregulation of VEGF and BDNF, Activation of the PI3K/Akt Pathway, and Increase of Neurogenesis Are Associated with Therapeutic Improvement after Traumatic Brain Injury. J Neurotrauma 25: 130-139.

121. Martinowich K, Manji H, Lu B (2007) New insights into BDNF function in depression and anxiety. Nat Neurosci 10: 1089-1093.

122. Griesbach GS, Hovda DA, Molteni R, Wu A, Gomez-Pinilla F (2004) Voluntary exercise following traumatic brain injury: brain-derived neurotrophic factor upregulation and recovery of function. Neuroscience 125: 129-139.

123. Babcock AM, Standing D, Bullshields K, Schwartz E, Paden CM et al. (2005) In vivo inhibition of hippocampal Ca2+/calmodulin-dependent protein kinase II by RNA interference. Mol Ther 11: 899-905.

124. Atkins CM, Chen S, Alonso OF, Dietrich WD, Hu BR (2006) Activation of calcium/calmodulin-dependent protein kinases after traumatic brain injury. J Cereb Blood Flow Metab 26: 1507-1518.

125. Haberman RP, Lee HJ, Colantuoni C, Koh MT, Gallagher M (2008) Rapid encoding of new information alters the profile of plasticity-related mRNA transcripts in the hippocampal CA3 region. Proc Natl Acad Sci U S A 105: 10601-10606.

126. Lund LM, McQuarrie IG (2002) Calcium/calmodulin-dependent protein kinase IIbeta isoform is expressed in motor neurons during axon outgrowth and is part of slow axonal transport. J Neurosci Res 67: 720-728.

127. Rohrer B, Guo Y, Kunchithapautham K, Gilkeson GS (2007) Eliminating complement factor D reduces photoreceptor susceptibility to light-induced damage. Invest Ophthalmol Vis Sci 48: 5282-5289.

128. Turnberg D, Lewis M, Moss J, Xu Y, Botto M et al. (2006) Complement activation contributes to both glomerular and tubulointerstitial damage in adriamycin nephropathy in mice. J Immunol 177: 4094-4102.

129. Mostert JP, Koch MW, Heerings M, Heersema DJ, De KJ (2008) Therapeutic potential of fluoxetine in neurological disorders. CNS Neurosci Ther 14: 153-164.

130. Tully T, Bourtchouladze R, Scott R, Tallman J (2003) Targeting the CREB pathway for memory enhancers. Nat Rev Drug Discov 2: 267-277.

131. Alberini CM (2009) Transcription factors in long-term memory and synaptic plasticity. Physiol Rev 89: 121-145.

132. Chang YC, Tzeng SF, Yu L, Huang AM, Lee HT et al. (2006) Early-life fluoxetine exposure reduced functional deficits after hypoxic-ischemia brain injury in rat pups. Neurobiol Dis 24: 101-113.

133. Brechet A, Fache MP, Brachet A, Ferracci G, Baude A et al. (2008) Protein kinase CK2 contributes to the organization of sodium channels in axonal membranes by regulating their interactions with ankyrin G. J Cell Biol 183: 1101-1114.

134. Gottlieb DJ, O'Connor GT, Wilk JB (2007) Genome-wide association of sleep and circadian phenotypes. BMC Med Genet 8 Suppl 1: S9.

135. Escalier D, Silvius D, Xu X (2003) Spermatogenesis of mice lacking CK2alpha': failure of germ cell survival and characteristic modifications of the spermatid nucleus. Mol Reprod Dev 66: 190-201.

136. Lee JH, Kim KY, Lee YK, Park SY, Kim CD et al. (2004) Cilostazol prevents focal cerebral ischemic injury by enhancing casein kinase 2 phosphorylation and suppression of phosphatase and tensin homolog deleted from chromosome 10 phosphorylation in rats. J Pharmacol Exp Ther 308: 896-903.

137. Wolvetang EJ, Bradfield OM, Hatzistavrou T, Crack PJ, Busciglio J et al. (2003) Overexpression of the chromosome 21 transcription factor Ets2 induces neuronal apoptosis. Neurobiol Dis 14: 349-356.

138. Bessho Y, Kageyama R (2003) Oscillations, clocks and segmentation. Curr Opin Genet Dev 13: 379-384.

139. Kageyama R, Ohtsuka T, Tomita K (2000) The bHLH gene Hes1 regulates differentiation of multiple cell types. Mol Cells 10: 1-7.

140. Kageyama R, Ohtsuka T, Kobayashi T (2008) Roles of Hes genes in neural development. Dev Growth Differ 50 Suppl 1: S97-103.

141. Tappe A, Klugmann M, Luo C, Hirlinger D, Agarwal N et al. (2006) Synaptic scaffolding protein Homer1a protects against chronic inflammatory pain. Nat Med 12: 677-681.

142. Ango F, Robbe D, Tu JC, Xiao B, Worley PF et al. (2002) Homer-dependent cell surface expression of metabotropic glutamate receptor type 5 in neurons. Mol Cell Neurosci 20: 323-329.

143. Rickhag M, Wieloch T, Gido G, Elmer E, Krogh M et al. (2006) Comprehensive regional and temporal gene expression profiling of the rat brain during the first 24 h after experimental stroke identifies dynamic ischemia-induced gene expression patterns, and reveals a biphasic activation of genes in surviving tissue. J Neurochem 96: 14-29.

144. Maret S, Dorsaz S, Gurcel L, Pradervand S, Petit B et al. (2007) Homer1a is a core brain molecular correlate of sleep loss. Proc Natl Acad Sci U S A 104: 20090-20095.

145. Sakamoto K, Yoshida S, Ikegami K, Minakami R, Kato A et al. (2007) Homer1c interacts with Hippi and protects striatal neurons from apoptosis. Biochem Biophys Res Commun 352: 1-5.

146. Jaubert PJ, Golub MS, Lo YY, Germann SL, Dehoff MH et al. (2007) Complex, multimodal behavioral profile of the Homer1 knockout mouse. Genes Brain Behav 6: 141-154.

147. Mizutani A, Kuroda Y, Futatsugi A, Furuichi T, Mikoshiba K (2008) Phosphorylation of Homer3 by calcium/calmodulin-dependent kinase II regulates a coupling state of its target molecules in Purkinje cells. J Neurosci 28: 5369-5382.

148. Parisiadou L, Bethani I, Michaki V, Krousti K, Rapti G et al. (2008) Homer2 and Homer3 interact with amyloid precursor protein and inhibit Abeta production. Neurobiol Dis 30: 353-364.

149. Huang GN, Huso DL, Bouyain S, Tu J, McCorkell KA et al. (2008) NFAT binding and regulation of T cell activation by the cytoplasmic scaffolding Homer proteins. Science 319: 476-481.

150. Bittner MA (2000) Alpha-latrotoxin and its receptors CIRL (latrophilin) and neurexin 1 alpha mediate effects on secretion through multiple mechanisms. Biochimie 82: 447-452.

151. Yoshida D, Nomura R, Teramoto A (2008) Regulation of cell invasion and signalling pathways in the pituitary adenoma cell line, HP-75, by reversion-inducing cysteine-rich protein with kazal motifs (RECK). J Neurooncol 89: 141-150.

152. Atkins CM, Oliva AA, Jr., Alonso OF, Chen S, Bramlett HM et al. (2007) Hypothermia treatment potentiates ERK1/2 activation after traumatic brain injury. Eur J Neurosci 26: 810-819.

153. Vauzour D, Vafeiadou K, Rice-Evans C, Williams RJ, Spencer JP (2007) Activation of pro-survival Akt and ERK1/2 signalling pathways underlie the anti-apoptotic effects of flavanones in cortical neurons. J Neurochem 103: 1355-1367.

154. Gomes E, Papa L, Hao T, Rockwell P (2007) The VEGFR2 and PKA pathways converge at MEK/ERK1/2 to promote survival in serum deprived neuronal cells. Mol Cell Biochem 305: 179-190.

155. Husemann J, Loike JD, Anankov R, Febbraio M, Silverstein SC (2002) Scavenger receptors in neurobiology and neuropathology: their role on microglia and other cells of the nervous system. GLIA 40: 195-205.

156. Bell MD, Lopez-Gonzalez R, Lawson L, Hughes D, Fraser I et al. (1994) Upregulation of the macrophage scavenger receptor in response to different forms of injury in the CNS. J Neurocytol 23: 605-613.

157. Ferrari Toninelli G., Bernardi C, Quarto M, Lozza G, Memo M et al. (2003) Long-lasting induction of Notch2 in the hippocampus of kainate-treated adult mice. Neuroreport 14: 917-921.

158. Royo NC, Conte V, Saatman KE, Shimizu S, Belfield CM et al. (2006) Hippocampal vulnerability following traumatic brain injury: a potential role for neurotrophin-4/5 in pyramidal cell neuroprotection. Eur J Neurosci 23: 1089-1102.

159. Royo NC, Lebold D, Magge SN, Chen I, Hauspurg A et al. (2007) Neurotrophin-mediated neuroprotection of hippocampal neurons following traumatic brain injury is not associated with acute recovery of hippocampal function. Neuroscience 148: 359-370.

160. Lin HW, Jain MR, Li H, Levison SW (2009) Ciliary neurotrophic factor (CNTF) plus soluble CNTF receptor alpha increases cyclooxygenase-2 expression, PGE2 release and interferon-gamma-induced CD40 in murine microglia. J Neuroinflammation 6: 7.

161. Hwang IK, Yi SS, Yoo KY, Park OK, Yan B et al. (2010) Effects of treadmill exercise on cyclooxygenase-2 in the hippocampus in type 2 diabetic rats: Correlation with the neuroblasts. Brain Res 1341: 84-92.

162. Strauss KI, Barbe MF, Marshall RM, Raghupathi R, Mehta S et al. (2000) Prolonged cyclooxygenase-2 induction in neurons and glia following traumatic brain injury in the rat. J Neurotrauma 17: 695-711.

163. O'Keeffe M, Grumont RJ, Hochrein H, Fuchsberger M, Gugasyan R et al. (2005) Distinct roles for the NF-kappaB1 and c-Rel transcription factors in the differentiation and survival of plasmacytoid and conventional dendritic cells activated by TLR-9 signals. Blood 106: 3457-3464.

164. Kuntzen C, Zazzeroni F, Pham CG, Papa S, Bubici C et al. (2007) A method for isolating prosurvival targets of NF-kappaB/Rel transcription factors. Methods Mol Biol 399: 99-124.

165. Vallabhapurapu S, Karin M (2009) Regulation and function of NF-kappaB transcription factors in the immune system. Annu Rev Immunol 27: 693-733.

166. Degnan BM, Vervoort M, Larroux C, Richards GS (2009) Early evolution of metazoan transcription factors. Curr Opin Genet Dev 19: 591-599.

167. Miletic G, Miyabe T, Gebhardt KJ, Miletic V (2005) Increased levels of Homer1b/c and Shank1a in the post-synaptic density of spinal dorsal horn neurons are associated with neuropathic pain in rats. Neurosci Lett 386: 189-193.

168. Sala C, Piech V, Wilson NR, Passafaro M, Liu G et al. (2001) Regulation of dendritic spine morphology and synaptic function by Shank and Homer. Neuron 31: 115-130.

169. Sala C, Roussignol G, Meldolesi J, Fagni L (2005) Key role of the postsynaptic density scaffold proteins Shank and Homer in the functional architecture of Ca2+ homeostasis at dendritic spines in hippocampal neurons. J Neurosci 25: 4587-4592.

170. Hung AY, Futai K, Sala C, Valtschanoff JG, Ryu J et al. (2008) Smaller dendritic spines, weaker synaptic transmission, but enhanced spatial learning in mice lacking Shank1. J Neurosci 28: 1697-1708.

171. Buscarlet M, Perin A, Laing A, Brickman JM, Stifani S (2008) Inhibition of cortical neuron differentiation by Groucho/TLE1 requires interaction with WRPW, but not Eh1, repressor peptides. J Biol Chem 283: 24881-24888.

172. Yao J, Liu Y, Husain J, Lo R, Palaparti A et al. (1998) Combinatorial expression patterns of individual TLE proteins during cell determination and differentiation suggest non-redundant functions for mammalian homologs of Drosophila Groucho. Dev Growth Differ 40: 133-146.

173. Berrebi D, Bruscoli S, Cohen N, Foussat A, Migliorati G et al. (2003) Synthesis of glucocorticoid-induced leucine zipper (GILZ) by macrophages: an anti-inflammatory and immunosuppressive mechanism shared by glucocorticoids and IL-10. Blood 101: 729-738.

174. Asselin-Labat ML, David M, Biola-Vidamment A, Lecoeuche D, Zennaro MC et al. (2004) GILZ, a new target for the transcription factor FoxO3, protects T lymphocytes from interleukin-2 withdrawal-induced apoptosis. Blood 104: 215-223.

175. Eddleston J, Herschbach J, Wagelie-Steffen AL, Christiansen SC, Zuraw BL (2007) The anti-inflammatory effect of glucocorticoids is mediated by glucocorticoid-induced leucine zipper in epithelial cells. J Allergy Clin Immunol 119: 115-122.

176. Nag S, Papneja T, Venugopalan R, Stewart DJ (2005) Increased angiopoietin2 expression is associated with endothelial apoptosis and blood-brain barrier breakdown. Lab Invest 85: 1189-1198.

177. Zhu Y, Lee C, Shen F, Du R, Young WL et al. (2005) Angiopoietin-2 facilitates vascular endothelial growth factor-induced angiogenesis in the mature mouse brain. Stroke 36: 1533-1537.

178. Calicchio ML, Collins T, Kozakewich HP (2009) Identification of Signaling Systems in Proliferating and Involuting Phase Infantile Hemangiomas by Genome-Wide Transcriptional Profiling. Am J Pathol 174: 1638-1649.

179. Lin TN, Wang CK, Cheung WM, Hsu CY (2000) Induction of angiopoietin and Tie receptor mRNA expression after cerebral ischemia-reperfusion. J Cereb Blood Flow Metab 20: 387-395.

180. Christakos S, Barletta F, Huening M, Dhawan P, Liu Y et al. (2003) Vitamin D target proteins: function and regulation. J Cell Biochem 88: 238-244.

181. Hilton GD, Ndubuizu A, Nunez JL, McCarthy MM (2005) Simultaneous glutamate and GABA(A) receptor agonist administration increases calbindin levels and prevents hippocampal damage induced by either agent alone in a model of perinatal brain injury. Brain Res Dev Brain Res 159: 99-111.

182. Fan Y, Shi L, Gu Y, Zhao Y, Xie J et al. (2007) Pretreatment with PTD-calbindin D 28k alleviates rat brain injury induced by ischemia and reperfusion. J Cereb Blood Flow Metab 27: 719-728.

183. Ringger NC, Tolentino PJ, McKinsey DM, Pike BR, Wang KK et al. (2004) Effects of injury severity on regional and temporal mRNA expression levels of calpains and caspases after traumatic brain injury in rats. J Neurotrauma 21: 829-841.

184. Zakeri Z, Lockshin RA (2008) Cell death: history and future. Adv Exp Med Biol 615: 1-11.

185. Di GS, Movsesyan V, Ahmed F, Cernak I, Schinelli S et al. (2005) Cell cycle inhibition provides neuroprotection and reduces glial proliferation and scar formation after traumatic brain injury. Proc Natl Acad Sci U S A 102: 8333-8338.

186. Keane RW, Kraydieh S, Lotocki G, Alonso OF, Aldana P et al. (2001) Apoptotic and antiapoptotic mechanisms after traumatic brain injury. J Cereb Blood Flow Metab 21: 1189-1198.

187. Kudryashov IE, Yakovlev AA, Kudryashova IV, Gulyaeva NV (2004) Inhibition of caspase-3 blocks long-term potentiation in hippocampal slices. Neurosci Behav Physiol 34: 877-880.

188. Forster R, valos-Misslitz AC, Rot A (2008) CCR7 and its ligands: balancing immunity and tolerance. Nat Rev Immunol 8: 362-371.

189. Khader SA, Rangel-Moreno J, Fountain JJ, Martino CA, Reiley WW et al. (2009) In a murine tuberculosis model, the absence of homeostatic chemokines delays granuloma formation and protective immunity. J Immunol 183: 8004-8014.

190. Robertson MJ (2002) Role of chemokines in the biology of natural killer cells. J Leukoc Biol 71: 173-183.

191. Garcia-Domingo D, Leonardo E, Grandien A, Martinez P, Albar JP et al. (1999) DIO-1 is a gene involved in onset of apoptosis in vitro, whose misexpression disrupts limb development. Proc Natl Acad Sci U S A 96: 7992-7997.

192. Garcia-Domingo D, Ramirez D, Gonzalez de BG, Martinez A (2003) Death inducer-obliterator 1 triggers apoptosis after nuclear translocation and caspase upregulation. Mol Cell Biol 23: 3216-3225.

193. Boutros T, Nantel A, Emadali A, Tzimas G, Conzen S et al. (2008) The MAP kinase phosphatase-1 MKP-1/DUSP1 is a regulator of human liver response to transplantation. Am J Transplant 8: 2558-2568.

194. Kuwano Y, Gorospe M (2008) Protecting the stress response, guarding the MKP-1 mRNA. Cell Cycle 7: 2640-2642.

195. Nagata T, Takahashi Y, Sugahara M, Murata A, Nishida Y et al. (2004) Profiling of genes associated with transcriptional responses in mouse hippocampus after transient forebrain ischemia using high-density oligonucleotide DNA array. Brain Res Mol Brain Res 121: 1-11.

196. Kondoh K, Sunadome K, Nishida E (2007) Notch signaling suppresses p38 MAPK activity via induction of MKP-1 in myogenesis. J Biol Chem 282: 3058-3065.

197. Sgambato V, Pages C, Rogard M, Besson MJ, Caboche J (1998) Extracellular signal-regulated kinase (ERK) controls immediate early gene induction on corticostriatal stimulation. J Neurosci 18: 8814-8825.

198. Galvin KE, Ye H, Erstad DJ, Feddersen R, Wetmore C (2008) Gli1 induces G2/M arrest and apoptosis in hippocampal but not tumor-derived neural stem cells. Stem cells 26: 1027-1036.

199. Bambakidis NC, Horn EM, Nakaji P, Theodore N, Bless E et al. (2009) Endogenous stem cell proliferation induced by intravenous hedgehog agonist administration after contusion in the adult rat spinal cord. J Neurosurg Spine 10: 171-176.

200. Matise MP, Joyner AL (1999) Gli genes in development and cancer. Oncogene 18: 7852-7859.

201. Dormoy V, Danilin S, Lindner V, Thomas L, Rothhut S et al. (2009) The sonic hedgehog signaling pathway is reactivated in human renal cell carcinoma and plays orchestral role in tumor growth. Mol Cancer 8: 123.

202. Giubellino A, Burke TR, Jr., Bottaro DP (2008) Grb2 signaling in cell motility and cancer. Expert Opin Ther Targets 12: 1021-1033.

203. Thornhill PB, Cohn JB, Stanford WL, Desbarats J (2008) The adaptor protein Grb2 regulates cell surface Fas ligand in Schwann cells. Biochem Biophys Res Commun 376: 341-346.

204. Russo C, Dolcini V, Salis S, Venezia V, Violani E et al. (2002) Signal transduction through tyrosine-phosphorylated carboxy-terminal fragments of APP via an enhanced interaction with Shc/Grb2 adaptor proteins in reactive astrocytes of Alzheimer's disease brain. Ann N Y Acad Sci 973: 323-333.

205. Venezia V, Russo C, Repetto E, Salis S, Dolcini V et al. (2004) Apoptotic cell death influences the signaling activity of the amyloid precursor protein through ShcA and Grb2 adaptor proteins in neuroblastoma SH-SY5Y cells. J Neurochem 90: 1359-1370.

206. Jang IK, Zhang J, Gu H (2009) Grb2, a simple adapter with complex roles in lymphocyte development, function, and signaling. Immunol Rev 232: 150-159.

207. Guibinga GH, Hsu S, Friedmann T (2010) Deficiency of the housekeeping gene hypoxanthine-guanine phosphoribosyltransferase (HPRT) dysregulates neurogenesis. Mol Ther 18: 54-62.

208. Torres RJ, Puig JG (2007) Hypoxanthine-guanine phosophoribosyltransferase (HPRT) deficiency: Lesch-Nyhan syndrome. Orphanet J Rare Dis 2: 48.

209. Shworak NW, HajMohammadi S, de Agostini AI, Rosenberg RD (2002) Mice deficient in heparan sulfate 3-O-sulfotransferase-1: normal hemostasis with unexpected perinatal phenotypes. Glycoconj J 19: 355-361.

210. Zhang L, Schwartz JJ, Miller J, Liu J, Fritze LM et al. (1998) The retinoic acid and cAMP-dependent up-regulation of 3-O-sulfotransferase-1 leads to a dramatic augmentation of anticoagulantly active heparan sulfate biosynthesis in F9 embryonal carcinoma cells. J Biol Chem 273: 27998-28003.

211. Cui J, Zhu N, Wang Q, Yu M, Feng J et al. (2009) p38 MAPK contributes to CD54 expression and the enhancement of phagocytic activity during macrophage development. Cell Immunol 256: 6-11.

212. Dietrich JB (2002) The adhesion molecule ICAM-1 and its regulation in relation with the blood-brain barrier. J Neuroimmunol 128: 58-68.

213. Chen Q, Appenheimer MM, Muhitch JB, Fisher DT, Clancy KA et al. (2009) Thermal facilitation of lymphocyte trafficking involves temporal induction of intravascular ICAM-1. Microcirculation 16: 143-158.

214. Beilharz EJ, Russo VC, Butler G, Baker NL, Connor B et al. (1998) Co-ordinated and cellular specific induction of the components of the IGF/IGFBP axis in the rat brain following hypoxic-ischemic injury. Brain Res Mol Brain Res 59: 119-134.

215. Ren H, Yin P, Duan C (2008) IGFBP-5 regulates muscle cell differentiation by binding to IGF-II and switching on the IGF-II auto-regulation loop. J Cell Biol 182: 979-991.

216. Beilharz EJ, Klempt ND, Klempt M, Sirimanne E, Dragunow M et al. (1993) Differential expression of insulin-like growth factor binding proteins (IGFBP) 4 and 5 mRNA in the rat brain after transient hypoxic-ischemic injury. Brain Res Mol Brain Res 18: 209-215.

217. Lee WH, Wang GM, Seaman LB, Vannucci SJ (1996) Coordinate IGF-I and IGFBP5 gene expression in perinatal rat brain after hypoxia-ischemia. J Cereb Blood Flow Metab 16: 227-236.

218. Wolf LV, Yang Y, Wang J, Xie Q, Braunger B et al. (2009) Identification of pax6-dependent gene regulatory networks in the mouse lens. PLoS One 4: e4159.

219. Cacalano NA, Le D, Paranjpe A, Wang MY, Fernandez A et al. (2008) Regulation of IGFBP6 gene and protein is mediated by the inverse expression and function of c-jun N-terminal kinase (JNK) and NFkappaB in a model of oral tumor cells. Apoptosis 13: 1439-1449.

220. Saito T, Akutsu S, Urushiyama T, Ishibashi K, Nakagawa Y et al. (2003) Changes in the mRNA expressions of insulin-like growth factors, their receptors, and binding proteins during the postnatal development of rat masseter muscle. Zoolog Sci 20: 441-447.

221. Fogal B, Hewett SJ (2008) Interleukin-1beta: a bridge between inflammation and excitotoxicity? J Neurochem 106: 1-23.

222. Bellehumeur C, Blanchet J, Fontaine JY, Bourcier N, Akoum A (2009) Interleukin 1 regulates its own receptors in human endometrial cells via distinct mechanisms. Hum Reprod 24: 2193-2204.

223. Franchi L, Eigenbrod T, Munoz-Planillo R, Nunez G (2009) The inflammasome: a caspase-1-activation platform that regulates immune responses and disease pathogenesis. Nat Immunol 10: 241-247.

224. Hutchinson PJ, O'Connell MT, Rothwell NJ, Hopkins SJ, Nortje J et al. (2007) Inflammation in human brain injury: intracerebral concentrations of IL-1alpha, IL-1beta, and their endogenous inhibitor IL-1ra. J Neurotrauma 24: 1545-1557.

225. Lu KT, Wu CY, Yen HH, Peng JH, Wang CL et al. (2007) Bumetanide administration attenuated traumatic brain injury through IL-1 overexpression. Neurol Res 29: 404-409.

226. Kim JV, Dustin ML (2006) Innate response to focal necrotic injury inside the blood-brain barrier. J Immunol 177: 5269-5277.

227. Gharibyan AL, Zamotin V, Yanamandra K, Moskaleva OS, Margulis BA et al. (2007) Lysozyme amyloid oligomers and fibrils induce cellular death via different apoptotic/necrotic pathways. J Mol Biol 365: 1337-1349.

228. Sato T, Torashima T, Sugihara K, Hirai H, Asano M et al. (2008) The scaffold protein JSAP1 regulates proliferation and differentiation of cerebellar granule cell precursors by modulating JNK signaling. Mol Cell Neurosci 39: 569-578.

229. Xu P, Yoshioka K, Yoshimura D, Tominaga Y, Nishioka T et al. (2003) In vitro development of mouse embryonic stem cells lacking JNK/stress-activated protein kinase-associated protein 1 (JSAP1) scaffold protein revealed its requirement during early embryonic neurogenesis. J Biol Chem 278: 48422-48433.

230. Iwanaga A, Sato T, Sugihara K, Hirao A, Takakura N et al. (2007) Neural-specific ablation of the scaffold protein JSAP1 in mice causes neonatal death. Neurosci Lett 429: 43-48.

231. Miura E, Fukaya M, Sato T, Sugihara K, Asano M et al. (2006) Expression and distribution of JNK/SAPK-associated scaffold protein JSAP1 in developing and adult mouse brain. J Neurochem 97: 1431-1446.

232. Rincon M, Davis RJ (2009) Regulation of the immune response by stress-activated protein kinases. Immunol Rev 228: 212-224.

233. Bourdi M, Korrapati MC, Chakraborty M, Yee SB, Pohl LR (2008) Protective role of c-Jun N-terminal kinase 2 in acetaminophen-induced liver injury. Biochem Biophys Res Commun 374: 6-10.

234. Gerykova-Bujalkova M, Krivulcik T, Bartosova Z (2008) Novel approaches in evaluation of pathogenicity of single-base exonic germline changes involving the mismatch repair genes MLH1 and MSH2 in diagnostics of Lynch syndrome. Neoplasma 55: 463-471.

235. Munday JS, French AF, Gibson IR, Gwynne K (2009) Widespread mismatch repair protein expression in canine cutaneous mast cell tumors. Vet Pathol 46: 227-232.

236. Loyer X, Heymes C, Samuel JL (2008) Constitutive nitric oxide synthases in the heart from hypertrophy to failure. Clin Exp Pharmacol Physiol 35: 483-488.

237. Mollsten A, Lajer M, Jorsal A, Tarnow L (2009) The endothelial nitric oxide synthase gene and risk of diabetic nephropathy and development of cardiovascular disease in type 1 diabetes. Mol Genet Metab 97: 80-84.

238. Zhang Y, Lu J, Shi J, Lin X, Dong J et al. (2008) Central administration of angiotensin-(1-7) stimulates nitric oxide release and upregulates the endothelial nitric oxide synthase expression following focal cerebral ischemia/reperfusion in rats. Neuropeptides 42: 593-600.

239. Morioka I, Tsuneishi S, Takada S, Matsuo M (2004) PDGF-alpha receptor expression following hypoxic-ischemic injury in the neonatal rat brain. Kobe J Med Sci 50: 21-30.

240. Perros F, Montani D, Dorfmuller P, Durand-Gasselin I, Tcherakian C et al. (2008) Platelet-derived growth factor expression and function in idiopathic pulmonary arterial hypertension. Am J Respir Crit Care Med 178: 81-88.

241. Ishii Y, Matsumoto Y, Watanabe R, Elmi M, Fujimori T et al. (2008) Characterization of neuroprogenitor cells expressing the PDGF beta-receptor within the subventricular zone of postnatal mice. Mol Cell Neurosci 37: 507-518.

242. Wu CY, Hsieh HL, Sun CC, Tseng CP, Yang CM (2008) IL-1 beta induces proMMP-9 expression via c-Src-dependent PDGFR/PI3K/Akt/p300 cascade in rat brain astrocytes. J Neurochem 105: 1499-1512.

243. Cohen ED, Ihida-Stansbury K, Lu MM, Panettieri RA, Jones PL et al. (2009) Wnt signaling regulates smooth muscle precursor development in the mouse lung via a tenascin C/PDGFR pathway. J Clin Invest 119: 2538-2549.

244. Schwartz JP, Nishiyama N (1994) Neurotrophic factor gene expression in astrocytes during development and following injury. Brain Res Bull 35: 403-407.

245. Rosenberger J, Petrovics G, Buzas B (2001) Oxidative stress induces proorphanin FQ and proenkephalin gene expression in astrocytes through p38- and ERK-MAP kinases and NF-kappaB. J Neurochem 79: 35-44.

246. McTavish N, Copeland LA, Saville MK, Perkins ND, Spruce BA (2007) Proenkephalin assists stress-activated apoptosis through transcriptional repression of NF-kappaB- and p53-regulated gene targets. Cell Death Differ 14: 1700-1710.

247. Eastwood SL, Salih T, Harrison PJ (2005) Differential expression of calcineurin A subunit mRNA isoforms during rat hippocampal and cerebellar development. Eur J Neurosci 22: 3017-3024.

248. Mathieu F, Miot S, Etain B, El Khoury MA, Chevalier F et al. (2008) Association between the PPP3CC gene, coding for the calcineurin gamma catalytic subunit, and bipolar disorder. Behav Brain Funct 4: 2.

249. Anantharam V, Lehrmann E, Kanthasamy A, Yang Y, Banerjee P et al. (2007) Microarray analysis of oxidative stress regulated genes in mesencephalic dopaminergic neuronal cells: relevance to oxidative damage in Parkinson's disease. Neurochem Int 50: 834-847.

250. Allen TR, Krueger KD, Hunter WJ, III, Agrawal DK (2005) Evidence that insulin-like growth factor-1 requires protein kinase C-epsilon, PI3-kinase and mitogen-activated protein kinase pathways to protect human vascular smooth muscle cells from apoptosis. Immunol Cell Biol 83: 651-667.

251. Uemura K, Aki T, Yamaguchi K, Yoshida K (2003) Protein kinase C-epsilon protects PC12 cells against methamphetamine-induced death: possible involvement of suppression of glutamate receptor. Life Sci 72: 1595-1607.

252. Malhotra A, Begley R, Kang BP, Rana I, Liu J et al. (2005) PKC-{epsilon}-dependent survival signals in diabetic hearts. Am J Physiol Heart Circ Physiol 289: H1343-H1350.

253. Shinohara H, Kayagaki N, Yagita H, Oyaizu N, Ohba M et al. (2001) A protective role of PKCepsilon against TNF-related apoptosis-inducing ligand (TRAIL)-induced apoptosis in glioma cells. Biochem Biophys Res Commun 284: 1162-1167.

254. Funk JL, Migliati E, Chen G, Wei H, Wilson J et al. (2003) Parathyroid hormone-related protein induction in focal stroke: a neuroprotective vascular peptide. Am J Physiol Regul Integr Comp Physiol 284: R1021-R1030.

255. Simmonds CS, Karsenty G, Karaplis AC, Kovacs CS (2009) Parathyroid Hormone Regulates Fetal-Placental Mineral Homeostasis. J Bone Miner Res .

256. Calvo NG, Gentili CR, de Boland AR (2008) The early phase of programmed cell death in Caco-2 intestinal cells exposed to PTH. J Cell Biochem 105: 989-997.

257. Calvo N, German O, Russo de BA, Gentili C (2009) Pro-apoptotic effects of parathyroid hormone in intestinal cells. Biochem Cell Biol 87: 389-400.

258. Langdon YG, Goetz SC, Berg AE, Swanik JT, Conlon FL (2007) SHP-2 is required for the maintenance of cardiac progenitors. Development 134: 4119-4130.

259. Grossmann KS, Wende H, Paul FE, Cheret C, Garratt AN et al. (2009) The tyrosine phosphatase Shp2 (PTPN11) directs Neuregulin-1/ErbB signaling throughout Schwann cell development. Proc Natl Acad Sci U S A 106: 16704-16709.

260. Yang Z, Li Y, Yin F, Chan RJ (2008) Activating PTPN11 mutants promote hematopoietic progenitor cell-cycle progression and survival. Exp Hematol 36: 1285-1296.

261. Servidei T, Bhide PG, Huang Z, Moskowitz MA, Harsh G et al. (1998) The protein tyrosine phosphatase SHP-2 is expressed in glial and neuronal progenitor cells, postmitotic neurons and reactive astrocytes. Neuroscience 82: 529-543.

262. Yang T, Massa SM, Longo FM (2006) LAR protein tyrosine phosphatase receptor associates with TrkB and modulates neurotrophic signaling pathways. J Neurobiol 66: 1420-1436.

263. Petrone A, Battaglia F, Wang C, Dusa A, Su J et al. (2003) Receptor protein tyrosine phosphatase alpha is essential for hippocampal neuronal migration and long-term potentiation. EMBO J 22: 4121-4131.

264. Kostic A, Sap J, Sheetz MP (2007) RPTPalpha is required for rigidity-dependent inhibition of extension and differentiation of hippocampal neurons. J Cell Sci 120: 3895-3904.

265. Solomon DA, Kim JS, Cronin JC, Sibenaller Z, Ryken T et al. (2008) Mutational inactivation of PTPRD in glioblastoma multiforme and malignant melanoma. Cancer Res 68: 10300-10306.

266. Uetani N, Kato K, Ogura H, Mizuno K, Kawano K et al. (2000) Impaired learning with enhanced hippocampal long-term potentiation in PTPdelta-deficient mice. EMBO J 19: 2775-2785.

267. Cheng Q, Di L, V, Caniglia G, Mudo G (2008) Time-course of GDNF and its receptor expression after brain injury in the rat. Neurosci Lett 439: 24-29.

268. Michos O, Cebrian C, Hyink D, Grieshammer U, Williams L et al. (2010) Kidney development in the absence of Gdnf and Spry1 requires Fgf10. PLoS Genet 6: e1000809.

269. Canibano C, Rodriguez NL, Saez C, Tovar S, Garcia-Lavandeira M et al. (2007) The dependence receptor Ret induces apoptosis in somatotrophs through a Pit-1/p53 pathway, preventing tumor growth. EMBO J 26: 2015-2028.

270. Doxakis E, Wyatt S, Davies AM (2000) Depolarisation causes reciprocal changes in GFR(alpha)-1 and GFR(alpha)-2 receptor expression and shifts responsiveness to GDNF and neurturin in developing neurons. Development 127: 1477-1487.

271. Tsujino H, Mansur K, Kiryu-Seo S, Namikawa K, Kitahara T et al. (1999) Discordant expression of c-Ret and glial cell line-derived neurotrophic factor receptor alpha-1 mRNAs in response to motor nerve injury in neonate rats. Brain Res Mol Brain Res 70: 298-303.

272. Miyazaki H, Nagashima K, Okuma Y, Nomura Y (2002) Expression of Ret receptor tyrosine kinase after transient forebrain ischemia is modulated by glial cell line-derived neurotrophic factor in rat hippocampus. Neurosci Lett 318: 1-4.

273. Burazin TC, Gundlach AL (1998) Up-regulation of GDNFR-alpha and c-ret mRNA in facial motor neurons following facial nerve injury in the rat. Brain Res Mol Brain Res 55: 331-336.

274. Zhang WH, Wang X, Narayanan M, Zhang Y, Huo C et al. (2003) Fundamental role of the Rip2/caspase-1 pathway in hypoxia and ischemia-induced neuronal cell death. Proc Natl Acad Sci U S A 100: 16012-16017.

275. Chin AI, Dempsey PW, Bruhn K, Miller JF, Xu Y et al. (2002) Involvement of receptor-interacting protein 2 in innate and adaptive immune responses. Nature 416: 190-194.

276. Stein SC, Chen XH, Sinson GP, Smith DH (2002) Intravascular coagulation: a major secondary insult in nonfatal traumatic brain injury. J Neurosurg 97: 1373-1377.

277. Grenander A, Bredbacka S, Rydvall A, Aroch R, Edner G et al. (2001) Antithrombin treatment in patients with traumatic brain injury: a pilot study. J Neurosurg Anesthesiol 13: 49-56.

278. Arai M, Goto T, Seichi A, Nakamura K (2004) Effects of antithrombin III on spinal cord-evoked potentials and functional recovery after spinal cord injury in rats. Spine 29: 405-412.

279. Smith R, Klein P, Koc-Schmitz Y, Waldvogel HJ, Faull RL et al. (2007) Loss of SNAP-25 and rabphilin 3a in sensory-motor cortex in Huntington's disease. J Neurochem 103: 115-123.

280. Forero DA, Arboleda GH, Vasquez R, Arboleda H (2009) Candidate genes involved in neural plasticity and the risk for attention-deficit hyperactivity disorder: a meta-analysis of 8 common variants. J Psychiatry Neurosci 34: 361-366.

281. Lema Tome CM, Nottingham CU, Smith CM, Beauchamp AS, Leung PW et al. (2006) Neonatal exposure to MK801 induces structural reorganization of the central nervous system. Neuroreport 17: 779-783.

282. Endo H, Nito C, Kamada H, Yu F, Chan PH (2006) Reduction in oxidative stress by superoxide dismutase overexpression attenuates acute brain injury after subarachnoid hemorrhage via activation of Akt/glycogen synthase kinase-3beta survival signaling. J Cereb Blood Flow Metab 27: 975-982.

283. Sugawara T, Noshita N, Lewen A, Gasche Y, Ferrand-Drake M et al. (2002) Overexpression of copper/zinc superoxide dismutase in transgenic rats protects vulnerable neurons against ischemic damage by blocking the mitochondrial pathway of caspase activation. J Neurosci 22: 209-217.

284. Cho JH, Hwang IK, Yoo KY, Kim SY, Kim DW et al. (2008) Effective delivery of Pep-1-cargo protein into ischemic neurons and long-term neuroprotection of Pep-1-SOD1 against ischemic injury in the gerbil hippocampus. Neurochem Int 52: 659-668.

285. Pun S, Santos AF, Saxena S, Xu L, Caroni P (2006) Selective vulnerability and pruning of phasic motoneuron axons in motoneuron disease alleviated by CNTF. Nat Neurosci 9: 408-419.

286. Bradford M, Law MH, Stewart AD, Shaw DJ, Megson IL et al. (2009) The TGM2 gene is associated with schizophrenia in a British population. Am J Med Genet B Neuropsychiatr Genet 150B: 335-340.

287. Tolentino PJ, DeFord SM, Notterpek L, Glenn CC, Pike BR et al. (2002) Up-regulation of tissue-type transglutaminase after traumatic brain injury. J Neurochem 80: 579-588.

288. Ientile R, Caccamo D, Marciano MC, Curro M, Mannucci C et al. (2004) Transglutaminase activity and transglutaminase mRNA transcripts in gerbil brain ischemia. Neurosci Lett 363: 173-177.

289. Tang SC, Arumugam TV, Xu X, Cheng A, Mughal MR et al. (2007) Pivotal role for neuronal Toll-like receptors in ischemic brain injury and functional deficits. Proc Natl Acad Sci U S A 104: 13798-13803.

290. Lehnardt S, Lehmann S, Kaul D, Tschimmel K, Hoffmann O et al. (2007) Toll-like receptor 2 mediates CNS injury in focal cerebral ischemia. J Neuroimmunol 190: 28-33.

291. Babcock AA, Wirenfeldt M, Holm T, Nielsen HH, ssing-Olesen L et al. (2006) Toll-like receptor 2 signaling in response to brain injury: an innate bridge to neuroinflammation. J Neurosci 26: 12826-12837.

292. Yao H, Felfly H, Wang J, Zhou D, Haddad GG (2009) DIDS protects against neuronal injury by blocking Toll-like receptor 2 activated-mechanisms. J Neurochem 108: 835-846.

293. Walton M, Sirimanne E, Reutelingsperger C, Williams C, Gluckman P et al. (1997) Annexin V labels apoptotic neurons following hypoxia-ischemia. Neuroreport 8: 3871-3875.

294. Matsuda R, Kaneko N, Kikuchi M, Chiwaki F, Toda M et al. (2003) Clinical significance of measurement of plasma annexin V concentration of patients in the emergency room. Resuscitation 57: 171-177.

295. Lorberboym M, Blankenberg FG, Sadeh M, Lampl Y (2006) In vivo imaging of apoptosis in patients with acute stroke: correlation with blood-brain barrier permeability. Brain Res 1103: 13-19.

296. Chen S, Atkins CM, Liu CL, Alonso OF, Dietrich WD et al. (2007) Alterations in mammalian target of rapamycin signaling pathways after traumatic brain injury. J Cereb Blood Flow Metab 27: 939-949.

297. Tang SJ, Reis G, Kang H, Gingras AC, Sonenberg N et al. (2002) A rapamycin-sensitive signaling pathway contributes to long-term synaptic plasticity in the hippocampus. Proc Natl Acad Sci U S A 99: 467-472.

298. Azar R, Najib S, Lahlou H, Susini C, Pyronnet S (2008) Phosphatidylinositol 3-kinase-dependent transcriptional silencing of the translational repressor 4E-BP1. Cell Mol Life Sci 65: 3110-3117.

299. Martin dl, V, Burda J, Nemethova M, Quevedo C, Alcazar A et al. (2001) Possible mechanisms involved in the down-regulation of translation during transient global ischaemia in the rat brain. Biochem J 357: 819-826.

300. Kobarg CB, Kobarg J, Crosara-Alberto DP, Theizen TH, Franchini KG (2005) MEF2C DNA-binding activity is inhibited through its interaction with the regulatory protein Ki-1/57. FEBS Lett 579: 2615-2622.

301. Nery FC, Rui E, Kuniyoshi TM, Kobarg J (2006) Evidence for the interaction of the regulatory protein Ki-1/57 with p53 and its interacting proteins. Biochem Biophys Res Commun 341: 847-855.

302. Nery FC, Passos DO, Garcia VS, Kobarg J (2004) Ki-1/57 interacts with RACK1 and is a substrate for the phosphorylation by phorbol 12-myristate 13-acetate-activated protein kinase C. J Biol Chem 279: 11444-11455.

303. Gloire G, Erneux C, Piette J (2007) The role of SHIP1 in T-lymphocyte life and death. Biochem Soc Trans 35: 277-280.

304. Kisseleva MV, Cao L, Majerus PW (2002) Phosphoinositide-specific inositol polyphosphate 5-phosphatase IV inhibits Akt/protein kinase B phosphorylation and leads to apoptotic cell death. J Biol Chem 277: 6266-6272.

305. Mori M, Burgess DL, Gefrides LA, Foreman PJ, Opferman JT et al. (2004) Expression of apoptosis inhibitor protein Mcl1 linked to neuroprotection in CNS neurons. Cell Death Differ 11: 1223-1233.

306. Zhan Q, Bieszczad CK, Bae I, Fornace AJ, Jr., Craig RW (1997) Induction of BCL2 family member MCL1 as an early response to DNA damage. Oncogene 14: 1031-1039.

307. Fujise K, Zhang D, Liu J, Yeh ET (2000) Regulation of apoptosis and cell cycle progression by MCL1. Differential role of proliferating cell nuclear antigen. J Biol Chem 275: 39458-39465.

308. Arbour N, Vanderluit JL, Le Grand JN, Jahani-Asl A, Ruzhynsky VA et al. (2008) Mcl-1 is a key regulator of apoptosis during CNS development and after DNA damage. J Neurosci 28: 6068-6078.

309. Malin S, McManus S, Busslinger M (2010) STAT5 in B cell development and leukemia. Curr Opin Immunol 22: 168-176.

310. Kim YJ, Yoon SY, Kim JT, Choi SC, Lim JS et al. (2009) NDRG2 suppresses cell proliferation through down-regulation of AP-1 activity in human colon carcinoma cells. Int J Cancer 124: 7-15.

311. Hu XL, Liu XP, Deng YC, Lin SX, Wu L et al. (2006) Expression analysis of the NDRG2 gene in mouse embryonic and adult tissues. Cell Tissue Res 325: 67-76.

312. Takahashi K, Yamada M, Ohata H, Honda K, Yamada M (2005) Ndrg2 promotes neurite outgrowth of NGF-differentiated PC12 cells. Neurosci Lett 388: 157-162.

313. Liu N, Wang L, Li X, Yang Q, Liu X et al. (2008) N-Myc downstream-regulated gene 2 is involved in p53-mediated apoptosis. Nucleic Acids Res 36: 5335-5349.

314. Shimohata T, Zhao H, Sung JH, Sun G, Mochly-Rosen D et al. (2007) Suppression of deltaPKC activation after focal cerebral ischemia contributes to the protective effect of hypothermia. J Cereb Blood Flow Metab 27: 1463-1475.

315. Chou WH, Messing RO (2008) Hypertensive encephalopathy and the blood-brain barrier: is deltaPKC a gatekeeper? J Clin Invest 118: 17-20.

316. Basu A, Tu H (2005) Activation of ERK during DNA damage-induced apoptosis involves protein kinase Cdelta. Biochem Biophys Res Commun 334: 1068-1073.

317. Basu A (2003) Involvement of protein kinase C-delta in DNA damage-induced apoptosis. J Cell Mol Med 7: 341-350.

318. Ahmed S, Shibazaki M, Takeuchi T, Kikuchi H (2005) Protein kinase Ctheta activity is involved in the 2,3,7,8-tetrachlorodibenzo-p-dioxin-induced signal transduction pathway leading to apoptosis in L-MAT, a human lymphoblastic T-cell line. FEBS J 272: 903-915.

319. Manicassamy S, Sun Z (2007) The critical role of protein kinase C-theta in Fas/Fas ligand-mediated apoptosis. J Immunol 178: 312-319.

320. Manicassamy S, Gupta S, Sun Z (2006) Selective function of PKC-theta in T cells. Cell Mol Immunol 3: 263-270.

321. Ali BR, Wasmeier C, Lamoreux L, Strom M, Seabra MC (2004) Multiple regions contribute to membrane targeting of Rab GTPases. J Cell Sci 117: 6401-6412.

322. Neumann-Haefelin E, Qi W, Finkbeiner E, Walz G, Baumeister R et al. (2008) SHC-1/p52Shc targets the insulin/IGF-1 and JNK signaling pathways to modulate life span and stress response in C. elegans. Genes Dev 22: 2721-2735.

323. McFarland KN, Wilkes SR, Koss SE, Ravichandran KS, Mandell JW (2006) Neural-specific inactivation of ShcA results in increased embryonic neural progenitor apoptosis and microencephaly. J Neurosci 26: 7885-7897.

324. Rosell DR, Akama KT, Nacher J, McEwen BS (2003) Differential expression of suppressors of cytokine signaling-1, -2, and -3 in the rat hippocampus after seizure: implications for neuromodulation by gp130 cytokines. Neuroscience 122: 349-358.

325. Ransome MI, Turnley AM (2008) Growth hormone signaling and hippocampal neurogenesis: insights from genetic models. Hippocampus 18: 1034-1050.

326. Rico-Bautista E, Flores-Morales A, Fernandez-Perez L (2006) Suppressor of cytokine signaling (SOCS) 2, a protein with multiple functions. Cytokine Growth Factor Rev 17: 431-439.

327. Polizzotto MN, Bartlett PF, Turnley AM (2000) Expression of "suppressor of cytokine signalling" (SOCS) genes in the developing and adult mouse nervous system. J Comp Neurol 423: 348-358.

328. Machado FS, Johndrow JE, Esper L, Dias A, Bafica A et al. (2006) Anti-inflammatory actions of lipoxin A4 and aspirin-triggered lipoxin are SOCS-2 dependent. Nat Med 12: 330-334.

329. Zhang Y, Xiong J, Wang J, Shi X, Bao G et al. (2008) Regulation of melanocyte apoptosis by Stathmin 1 expression. BMB Rep 41: 765-770.

330. Liu A, Stadelmann C, Moscarello M, Bruck W, Sobel A et al. (2005) Expression of stathmin, a developmentally controlled cytoskeleton-regulating molecule, in demyelinating disorders. J Neurosci 25: 737-747.

331. Mori N, Morii H (2002) SCG10-related neuronal growth-associated proteins in neural development, plasticity, degeneration, and aging. J Neurosci Res 70: 264-273.

332. Orlandi A, Hao H, Ferlosio A, Clement S, Hirota S et al. (2009) Alpha actin isoforms expression in human and rat adult cardiac conduction system. Differentiation 77: 360-368.

333. Matsson H, Eason J, Bookwalter CS, Klar j, Gustavsson P et al. (2008) Alpha-cardiac actin mutations produce atrial septal defects. Hum Mol Genet 17: 256-265.

334. Kaski JP, Syrris P, Burch M, Tome-Esteban MT, Fenton M et al. (2008) Idiopathic restrictive cardiomyopathy in children is caused by mutations in cardiac sarcomere protein genes. Heart 94: 1478-1484.

335. Rezvani M, Liew CC (2000) Role of the adenomatous polyposis coli gene product in human cardiac development and disease. J Biol Chem 275: 18470-18475.

336. Yanai H, Satoh K, Matsumine A, Akiyama T (2000) The colorectal tumour suppressor APC is present in the NMDA-receptor-PSD-95 complex in the brain. Genes Cells 5: 815-822.

337. Cadigan KM, Liu YI (2006) Wnt signaling: complexity at the surface. J Cell Sci 119: 395-402.

338. Davenport JW, Fernandes ER, Harris LD, Neale GA, Goorha R (1999) The mouse mitotic checkpoint gene bub1b, a novel bub1 family member, is expressed in a cell cycle-dependent manner. Genomics 55: 113-117.

339. Gasca S, Pellestor F, Assou S, Loup V, Anahory T et al. (2007) Identifying new human oocyte marker genes: a microarray approach. Reprod Biomed Online 14: 175-183.

340. Burum-Auensen E, DeAngelis PM, Schjolberg AR, Roislien J, Andersen SN et al. (2007) Spindle proteins Aurora A and BUB1B, but not Mad2, are aberrantly expressed in dysplastic mucosa of patients with longstanding ulcerative colitis. J Clin Pathol 60: 1403-1408.

341. Abal M, Obrador-Hevia A, Janssen KP, Casadome L, Menendez M et al. (2007) APC inactivation associates with abnormal mitosis completion and concomitant BUB1B/MAD2L1 up-regulation. Gastroenterology 132: 2448-2458.

342. Renz M, Langowski J (2008) Dynamics of the CapG actin-binding protein in the cell nucleus studied by FRAP and FCS. Chromosome Res 16: 427-437.

343. Hansson O, Strom K, Guner N, Wierup N, Sundler F et al. (2006) Inflammatory response in white adipose tissue in the non-obese hormone-sensitive lipase null mouse model. J Proteome Res 5: 1701-1710.

344. Silacci P, Mazzolai L, Gauci C, Stergiopulos N, Yin HL et al. (2004) Gelsolin superfamily proteins: key regulators of cellular functions. Cell Mol Life Sci 61: 2614-2623.

345. Kane R, Godson C, O'Brien C (2008) Chordin-like 1, a bone morphogenetic protein-4 antagonist, is upregulated by hypoxia in human retinal pericytes and plays a role in regulating angiogenesis. Mol Vis 14: 1138-1148.

346. Fujita T, Okada T, Hayashi S, Jahangeer S, Miwa N et al. (2004) Delta-catenin/NPRAP (neural plakophilin-related armadillo repeat protein) interacts with and activates sphingosine kinase 1. Biochem J 382: 717-723.

347. Cerruti MP (2006) Cri du Chat syndrome. Orphanet J Rare Dis 1: 33.

348. Kim K, Sirota A, Chen Yh YH, Jones SB, Dudek R et al. (2002) Dendrite-like process formation and cytoskeletal remodeling regulated by delta-catenin expression. Exp Cell Res 275: 171-184.

349. Hatzfeld M (2005) The p120 family of cell adhesion molecules. Eur J Cell Biol 84: 205-214.

350. Nemeth MJ, Kirby MR, Bodine DM (2006) Hmgb3 regulates the balance between hematopoietic stem cell self-renewal and differentiation. Proc Natl Acad Sci U S A 103: 13783-13788.

351. Somervaille TC, Matheny CJ, Spencer GJ, Iwasaki M, Rinn JL et al. (2009) Hierarchical maintenance of MLL myeloid leukemia stem cells employs a transcriptional program shared with embryonic rather than adult stem cells. Cell Stem Cell 4: 129-140.

352. Ahonen LJ, Kukkonen AM, Pouwels J, Bolton MA, Jingle CD et al. (2009) Perturbation of Incenp function impedes anaphase chromatid movements and chromosomal passenger protein flux at centromeres. Chromosoma 118: 71-84.

353. Hummer S, Mayer TU (2009) Cdk1 negatively regulates midzone localization of the mitotic kinesin mklp2 and the chromosomal passenger complex. Curr Biol 19: 607-612.

354. Pirity MK, Locker J, Schreiber-Agus N (2005) Rybp/DEDAF is required for early postimplantation and for central nervous system development. Mol Cell Biol 25: 7193-7202.

355. Stanton SE, Blanck JK, Locker J, Schreiber-Agus N (2007) Rybp interacts with Hippi and enhances Hippi-mediated apoptosis. Apoptosis 12: 2197-2206.

356. Pirity MK, Wang WL, Wolf LV, Tamm ER, Schreiber-Agus N et al. (2007) Rybp, a polycomb complex-associated protein, is required for mouse eye development. BMC Dev Biol 7: 39.

357. Garcia E, Marcos-Gutierrez C, del Mar LM, Moreno JC, Vidal M (1999) RYBP, a new repressor protein that interacts with components of the mammalian Polycomb complex, and with the transcription factor YY1. EMBO J 18: 3404-3418.

358. Viemann D, Barczyk K, Vogl T, Fischer U, Sunderkotter C et al. (2007) MRP8/MRP14 impairs endothelial integrity and induces a caspase-dependent and -independent cell death program. Blood 109: 2453-2460.

359. Nakatani Y, Yamazaki M, Chazin WJ, Yui S (2005) Regulation of S100A8/A9 (calprotectin) binding to tumor cells by zinc ion and its implication for apoptosis-inducing activity. Mediators Inflamm 2005: 280-292.

360. Alaedini A, Xiang Z, Kim H, Sung YJ, Latov N (2008) Up-regulation of apoptosis and regeneration genes in the dorsal root ganglia during cisplatin treatment. Exp Neurol 210: 368-374.

361. Engel S, Schluesener H, Mittelbronn M, Seid K, Adjodah D et al. (2000) Dynamics of microglial activation after human traumatic brain injury are revealed by delayed expression of macrophage-related proteins MRP8 and MRP14. Acta Neuropathol 100: 313-322.

362. Beschorner R, Engel S, Mittelbronn M, Adjodah D, Dietz K et al. (2000) Differential regulation of the monocytic calcium-binding peptides macrophage-inhibiting factor related protein-8 (MRP8/S100A8) and allograft inflammatory factor-1 (AIF-1) following human traumatic brain injury. Acta Neuropathol 100: 627-634.

363. Kimura T, Mogi C, Tomura H, Kuwabara A, Im DS et al. (2008) Induction of scavenger receptor class B type I is critical for simvastatin enhancement of high-density lipoprotein-induced anti-inflammatory actions in endothelial cells. J Immunol 181: 7332-7340.

364. Chang EH, Rigotti A, Huerta PT (2009) Age-related influence of the HDL receptor SR-BI on synaptic plasticity and cognition. Neurobiol Aging 30: 407-419.

365. Minge CE, Bennett BD, Norman RJ, Robker RL (2008) Peroxisome proliferator-activated receptor-gamma agonist rosiglitazone reverses the adverse effects of diet-induced obesity on oocyte quality. Endocrinology 149: 2646-2656.

366. Stein O, Thiery J, Stein Y (2002) Is there a genetic basis for resistance to atherosclerosis? Atherosclerosis 160: 1-10.

367. Oei SL, Shi Y (2001) Transcription factor Yin Yang 1 stimulates poly(ADP-ribosyl)ation and DNA repair. Biochem Biophys Res Commun 284: 450-454.

368. Rylski M, Amborska R, Zybura K, Mioduszewska B, Michaluk P et al. (2008) Yin Yang 1 is a critical repressor of matrix metalloproteinase-9 expression in brain neurons. J Biol Chem 283: 35140-35153.

369. Kim J, Kim JD (2008) In vivo YY1 knockdown effects on genomic imprinting. Hum Mol Genet 17: 391-401.

370. Chen L, Shioda T, Coser KR, Lynch MC, Yang C et al. (2010) Genome-wide analysis of YY2 versus YY1 target genes. Nucleic Acids Res 38: 4011-4026.

371. Korhonen P, Huotari V, Soininen H, Salminen A (1997) Glutamate-induced changes in the DNA-binding complexes of transcription factor YY1 in cultured hippocampal and cerebellar granule cells. Brain Res Mol Brain Res 52: 330-333.

372. Salmon M, Owens GK, Zehner ZE (2009) Over-expression of the transcription factor, ZBP-89, leads to enhancement of the C2C12 myogenic program. Biochim Biophys Acta 1793: 1144-1155.

373. Woo AJ, Moran TB, Schindler YL, Choe SK, Langer NB et al. (2008) Identification of ZBP-89 as a novel GATA-1-associated transcription factor involved in megakaryocytic and erythroid development. Mol Cell Biol 28: 2675-2689.

374. Bai L, Merchant JL (2000) Transcription factor ZBP-89 cooperates with histone acetyltransferase p300 during butyrate activation of p21waf1 transcription in human cells. J Biol Chem 275: 30725-30733.
